# Burden of liver cancer due to hepatitis C from 1990 to 2019 at the global, regional, and national levels

**DOI:** 10.3389/fonc.2023.1218901

**Published:** 2023-12-19

**Authors:** Jie Wei, Guoqing Ouyang, Guozhen Huang, Yong Wang, Shuangjiang Li, Jiaping Liu, Yanhong Zhang, Guandou Yuan, Songqing He

**Affiliations:** ^1^ Division of Hepatobiliary Surgery, The First Affiliated Hospital of Guangxi Medical University, Nanning, Guangxi, China; ^2^ Key Laboratory of Early Prevention and Treatment for Regional High Frequency Tumor (Guangxi Medical University), Ministry of Education, Nanning, Guangxi, China; ^3^ Guangxi Key Laboratory of Immunology and Metabolism for Liver Diseases, Guangxi Medical University, Nanning, Guangxi, China; ^4^ Comparative Oncology Laboratory, Schools of Veterinary Medicine and Medicine, University of California, Davis, Davis, CA, United States

**Keywords:** liver cancer due to hepatitis C, age-standardized incidence rate, age-standardized mortality rate, age-standardized incidence DALY rate, disease burden

## Abstract

**Background:**

Liver cancer due to hepatitis C (LCDHC) is one of the leading causes of cancer-related deaths worldwide, and the burden of LCDHC is increasing. We aimed to report the burden of LCDHC at the global, regional, and national levels in 204 countries from 1990 to 2019, stratified by etiology, sex, age, and Sociodemographic Index.

**Methods:**

Data on LCDHC were available from the Global Burden of Disease, Injuries, and Risk Factors (GBD) study 2019. Numbers and age-standardized mortality, incidence, and disability-adjusted life year (DALY) rates per 100,000 population were estimated through a systematic analysis of modeled data from the GBD 2019 study. The trends in the LCDHC burden were assessed using the annual percentage change.

**Results:**

Globally, in 2019, there were 152,225 new cases, 141,810 deaths, and 2,878,024 DALYs due to LCDHC. From 1990 to 2019, the number of incidences, mortality, and DALY cases increased by 80.68%, 67.50%, and 37.20%, respectively. However, the age-standardized incidence, mortality, and DALY rate had a decreasing trend during this period. In 2019, the highest age-standardized incidence rates (ASIRs) of LCDHC were found in high-income Asia Pacific, North Africa and the Middle East, and Central Asia. At the regional level, Mongolia, Egypt, and Japan had the three highest ASIRs in 2019. The incidence rates of LCDHC were higher in men and increased with age, with a peak incidence in the 95+ age group for women and the 85–89 age group for men in 2019. A nonlinear association was found between the age-standardized rates of LCDHC and sociodemographic index values at the regional and national levels.

**Conclusions:**

Although the age-standardized rates of LCDHC have decreased, the absolute numbers of incident cases, deaths, and DALYs have increased, indicating that LCDHC remains a significant global burden. In addition, the burden of LCDHC varies geographically. Male and older adult/s individuals have a higher burden of LCDHC. Our findings provide insight into the global burden trend of LCDHC. Policymakers should establish appropriate methods to achieve the HCV elimination target by 2030 and reducing the burden of LCDHC.

## Introduction

According to GLOBOCAN-2020 estimates, liver cancer is the sixth most diagnosed cancer and the third leading cause of cancer-related deaths worldwide, with 905,700 new cases and 830,200 deaths in 2020 (1). Primary liver cancer mainly includes three pathological types including hepatocellular carcinoma, intrahepatic cholangiocarcinoma, and mixed hepatocellular carcinoma and cholangiocarcinoma. Of total primary liver cancer, hepatocellular carcinoma accounts for 75%–85%, and intrahepatic cholangiocarcinoma accounts for 10%–15% (2). The main risk factors of liver cancer include prolonged infections caused by hepatitis A virus, hepatitis B virus (HBV), hepatitis C virus (HCV), hepatitis D virus, and hepatitis E virus, as well as alcoholic cirrhosis, obesity/type 2 diabetes, autoimmune hepatitis, nonalcoholic steatohepatitis, consumption of aflatoxin B1-contaminated food, and various dietary exposures (3–5). The probability of developing liver cancer during an individual’s lifespan can vary widely based on several factors. These factors include personal health, lifestyle choices, genetics, and exposure to risk factors. The treatment of liver cancer includes surgical resection, microwave or radiofrequency ablation, radiation therapy, transarterial embolization, immunotherapy, multi-targeted tyrosine kinase inhibitors, and liver transplantation. However, liver cancer is often detected at middle and advanced stages during the diagnosis, rendering surgical options unfeasible (6, 7). The efficacy of systemic therapies for advanced stages of liver cancer is still limited, with an overall 5-year survival rate of only 10% (2). Despite efforts to prevent liver cancer, its burden still exhibits an increasing trend (2, 6, 8).

HBV and HCV infections are known as the primary risk factors for liver cancer. However, most previous studies have mainly focused on HBV, with little epidemiological research conducted on HCV. HCV comprises six primary genotypes and has shown region variations: four of them are prevalent in low-income countries, whereas genotype-1 predominates in middle-income and high-income countries. HCV can lead to both acute and chronic hepatitis, liver cirrhosis, and liver cancer. The progression from HCV infection to the development of liver cancer is characterized by a gradual, protracted process that spans over 20–40 years in affected patients (9). Globally, in 2021, it was estimated that approximately 58 million people were living with chronic HCV infections (10). In the United States, the number of hepatocellular carcinoma cases due to HCV increased by 130% during 1990–1999 and 2000–2009 (11). Furthermore, it was reported that approximately 0.14 million deaths were attributable to liver cancer due to hepatitis C (LCDHC) in 2019 (4). Despite the burden of LCDHC based on regional and national factors being documented, comprehensive information on its epidemiology and burden, including the incidence, mortality, and disability-adjusted life years (DALYs), is lacking at the global, regional, and national levels. Therefore, there is an urgent need to gain a deeper understanding of LCDHC and allocate adequate resources for disease management and prevention.

In this study, we aimed to provide comprehensive and comparable information on the burden of LCDHC. To achieve this, we analyzed data from the Global Burden of Disease (GBD) 2019 study for global, regional, and national incidence, mortalities, and DALYs, which were presented as both numbers and age-standardized rates (ASRs), stratified by sex, age, and sociodemographic index (SDI). Our findings can be of valuable assistance to policymakers aiming to formulate strategies for addressing the challenges posed by LCDHC.

## Methods

### Overview and data source

The GBD 2019 study led by the Institute of Health Metrics and Evaluation was the largest and most comprehensive study of this type. The most recent update in 2019 analyzed epidemiological levels of 369 diseases and injuries, 282 causes of death, and 84 risk factors in 204 countries and territories, 21 regions, and 7 super-regions from 1990 to 2019 (12).

The GBD database is categorized by etiology, age, sex, country, and SDI. It classifies regions into five SDI categories: low, low-middle, middle, high-middle, and high. In addition, the 21 GBD regions are classified based on their geographical location. The SDI was calculated by synthesizing the gross domestic product, the educational level of people younger than 25 years, and the years of education of people older than 15 years. The index ranges from 0 (lowest) to 1 (highest) level (12). Data on incidence, deaths, DALYs, age-standardized incidence rate (ASIR), age-standardized mortality rate (ASMR), and age-standardized DALY rate (ASDR) were obtained from the Global Health Data Exchange query tool (http://gbdx.healthdata.org/gbd-results-tool) for global, regional, country, age, sex, and SDI levels, including information on LCDHC.

### Statistical analysis

To quantify trends in the global burden of LCDHC, the percentage change values of ASIR, ASMR, and ASDR were used. Positive or negative percentage change values were used to determine increasing or decreasing trends in the burden of LCDHC, respectively. The 95% uncertainty intervals (UIs) were determined as the 2.5th and 97.5th centiles of the ordered draws. All statistics were generated using R software version 3.6.3, and visualization was performed using the “ggplot2” package. Sex differences were analyzed using an unpaired *t* test, with statistical significance defined as a *P* value <0.05.

## Results

### Global burden of LCDHC

**Figure 1:**
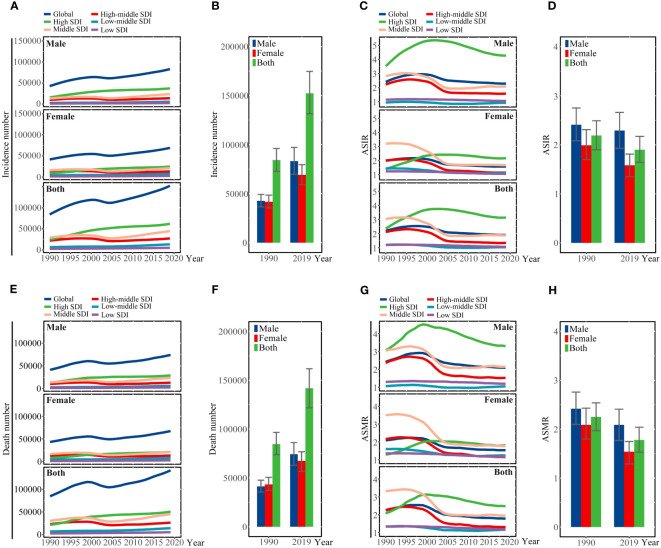
**(A)** Incidence number, **(C)** ASIR, **(E)** number of deaths, and **(G)** ASMR are illustrated for liver cancer due to hepatitis C at the global and regional levels from 1990 to 2019. **(B)** Incidence number, **(D)** ASIR, **(F)** number of deaths, and **(H)** ASMR of liver cancer due to hepatitis C at the global level in 1990 and 2019. SDI, sociodemographic index; ASIR, age-standardized incidence rate; ASMR, age-standardized mortality rate.

Globally, there were 152,225 (95%UI 131,581 to 174,627) new cases due to LCDHC in 2019, which represented an 80.68% increase from the 84,249 (95%UI 72,740 to 96,021) cases in 1990 (**Table 1**, **Figures 1A, B**). The global ASIR decreased from 2.18 (95%UI 1.90 to 2.49) per 100,000 populations in 1990 to 1.89 (95%UI 1.64 to 2.17) per 100,000 populations in 2019, with a percentage change of −0.13 (95%UI −0.22 to 0.02) (**Table 1**, **Figures 1C, D**). Moreover, in 2019, there were 141,810 (95%UI 121,787 to 161,828) death cases due to LCDHC, with an ASMR of 1.78 (95%UI 1.53 to 2.03) (**Table 1**, **Figures 1E–H**). In 1990, the number of deaths was 84,665 (95%UI 73,797 to 96,589), which increased by approximately 15.81% from 1990 to 2019. The estimated number of DALYs due to LCDHC in 1990 was 2,003,448 (95%UI 1,731,331 to 2,320,933) and in 2019 was 2,878,024 (95%UI 2,439,911 to 3,323, 494 (**Table 1**, **Additional file 2: Figure S1**). The ASDR of LCDHC in 1990 was 49.70 (95%UI 42.99 to 57.44) and in 2019 was 34.99 (95%UI 29.71 to 40.28) per 100,000 population, and this rate decreased by 29.59% (95%UI −36.94% to −20.47%) from 1990 to 2019 (**Table 1**, **Additional file 2: Figure S2).**

**Table 1:** Mortality, incident cases, and disability-adjusted life years (DALYs) for liver cancer due to hepatitis C in 2019 and percentage change in age-standardized rates (ASRs) per 100,000 population from 1990 to 2019 by Global Burden of Disease regions.

	Mortality (95% uncertainty interval)			Incidence (95% uncertainty interval)			DALYs (95% uncertainty interval)		
Location name	Number_2019	ASMR per 100,000 Population (95% UI) in 2019	Percentage change in ASMRs per 100,000 population (95% UI)	Number_2019	ASIR per 100,000 population(95% UI) in 2019	Percentage change in ASIRs per 100,000 population (95% UI)	Number_2019	ASDR per 100,000 population (95%UI) in 2019	Percentage change in ASDRs per 100,000 population(95% UI)
**Global**	141,811 (121,787 to 161,828)	1.78 (1.53 to 2.04)	-0.21 (-0.29 to 0.11)	152,225(131,581 to 174,627)	1.9 (1.64 to 2.17)	-0.13 (-0.22 to 0.02)	2,878,024 (2,439,911 to 3,323,494)	34.99 (29.71 to 40.28)	-0.3 (-0.37 to -0.2)
**Sex**									
**Male**	74,374 (62,929 to 86,178)	2.09(1.77 to 2.41)	-0.14 (-0.03 to -0.23)	83,116(69,769 to 97,165)	2.29(1.93 to 2.66)	-0.05(-0.16 to 0.08)	1,583,732 (1,304,982 to 1,873,404)	40.81 (33.63 to 48.27)	-0.25(-0.15 to 0.33)
**Female**	67,437 (56,530 to 76,812)	1.54(1.29 to 1.7)	-0.27 (-0.15 to-0.38)	69,109(59,012 to 79,581	1.58(1.35- 1.81)	-0.21 (-0.32 to 0.08)	1,294,292 (1,489,987 to 1,096,593)	33.56 (28.44 to 38.64)	-0.35(-0.23 to -0.45)
**High-middle SDI**	26,546 (22,661 to 30,483)	1.31 (1.12 to 1.5)	-0.42 (-0.49 to 0.34)	27,228 (23,250 to 31,618)	1.34 (1.14 to 1.55)	-0.37 (-0.45 to 0.27)	532,715 (452,992 to 616,546)	25.99 (22.19 to 30.01)	-0.47 (-0.54 to -0.39)
**High SDI**	50,524 (43,621 to 56,768)	2.48 (2.16 to 2.78)	0.19 (0.1 to 0.26)	61,953 (52,879 to 71,344)	3.13 (2.69 to 3.6)	0.33 (0.2 to 0.48)	877,177 (765,847 to 995,014)	47.19 (41.01 to 53.84)	-0.03 (-0.08 to -0.02)
**Low-middle SDI**	14,196 (11,418 to 16,829)	1.15 (0.93 to 1.35)	-0.14 (-0.27 to 0)	13,230 (10,652 to 15,820)	1.03 (0.84 to 1.23)	-0.15 (-0.26 to 0)	319,601 (252,731 to 387,260)	23.39 (18.64 to 28.23)	-0.16 (-0.28 to -0.02)
**Low SDI**	5,319 (4,145 to 6,466)	1.18 (0.94 to 1.41)	-0.11 (-0.22 to 0.02)	5,008 (3,879 to 6,120)	1.06 (0.83 to 1.28)	-0.16 (-0.27 to 0.03)	130,465 (99,685 to 160,687)	24.79 (19.14 to 30.46)	-0.11 (-0.23 to -0.02)
**Middle SDI**	45,168 (37,163 to 54,310)	1.95 (1.61 to 2.32)	-0.41 (-0.51 to 0.29)	44,749 (36,832 to 54,101)	1.87 (1.56 to 2.25)	-0.41 (-0.5 to 0.28)	1,016,857 (820,004 to 1,239,413)	40.13 (32.71 to 48.35)	-0.44 (-0.54 to -0.32)
**Australasia**	677 (512 to 863)	1.33 (1 to 1.69)	1.27 (1.07 to 1.49)	689 (489 to 947)	1.39 (0.99 to 1.91)	1.43 (0.96 to 1.98)	14,904 (10,198 to 20,036)	26.75 (18.78 to 35.32)	-0.13 (-0.33 to -0.15)
**Caribbean**	388 (263 to 537)	0.75 (0.51 to 1.04)	-0.52 (-0.59 to 0.44)	352 (239 to 498)	0.68 (0.46 to 0.96)	-0.51 (-0.59 to 0.43)	751,414 (609,844 to 900,127)	35.67 (29.24 to 42.58)	-0.66 (-0.73 to -0.58)
**Central Asia**	1,990 (1,422 to 2,599)	3.08 (2.25 to 3.95)	1.75 (1.34 to 2.24)	1,894 (1,332 to 2,494)	2.81 (2.04 to 3.63)	1.7 (1.28 to 2.18)	54,373 (44,568 to 65,512)	15.78 (13.01 to 18.96)	0.78 (0.58 to 0.99)
**Central Europe**	1,926 (1,408 to 2,566)	0.86 (0.63 to 1.15)	-0.45 (-0.53 to 0.36)	1,777 (1,296 to 2,390)	0.81 (0.59 to 1.08)	-0.42 (-0.51 to 0.33)	29,609 (21,757 to 38,928 )	18.7 (13.85 to 24.23)	0.04 (-0.19 to -0.31)
**Central Latin America**	3,385 (2,743 to 4,109)	1.49 (1.21 to 1.8)	-0.05 (-0.16 to 0.09)	3,097 (2,487 to 3,786)	1.35 (1.08 to 1.65)	-0.04 (-0.16 to 0.1)	2,735 (1,649 to 4,224)	4.98 (3 to 7.68)	-0.37 (-0.5 to -0.2)
**Central Sub-Saharan Africa**	565 (400 to 746)	1.24 (0.92 to 1.6)	-0.12 (-0.31 to 0.12)	542 (378 to 718)	1.12 (0.82 to 1.47)	-0.12 (-0.32 to 0.14)	438,136 (381,840 to 484,032)	98.19 (87.14 to 109.03)	-0.37 (-0.41 to -0.33)
**East Asia**	34,878 (28,702 to 41,388)	1.78 (1.49 to 2.1)	-0.64 (-0.71 to 0.55)	35,912 (29,625 to 42,897)	1.79 (1.48 to 2.12)	-0.6 (-0.68 to 0.5)	209,530 (177,240 to 243,188)	34.63 (29.28 to 40.27)	1.17 (0.95 to 1.37)
**Eastern Europe**	2,719 (2,283 to 3,211)	0.77 (0.65 to 0.91)	0.8 (0.62 to 1)	2,509 (2,067 to 3,006)	0.71 (0.59 to 0.85)	0.83 (0.65 to 1.04)	13,000 (9,662 to 16,800)	27.49 (20.36 to 35.58)	1.18 (0.99 to 1.4)
**Eastern Sub-Saharan Africa**	1,276 (952 to 1,637)	0.96 (0.73 to 1.21)	0.11 (-0.09 to 0.37)	1,166 (864 to 1,504)	0.83 (0.63 to 1.06)	0.08 (-0.12 to 0.33)	7,613 (4,943 to 11,100)	14.7 (9.58 to 21.4)	-0.52 (-0.6 to -0.43)
**Andean Latin America**	152 (94 to 232)	0.28 (0.18 to 0.43)	-0.31 (-0.45 to 0.14)	133 (81 to 204)	0.25 (0.15 to 0.38)	-0.32 (-0.46 to 0.14)	47,951 (32,811 to 64,423)	64.25 (45.62 to 83.96)	1.57 (1.17 to 2.05)
**High-income Asia Pacific**	27,963 (23,725 to 30,944)	5.42 (4.7 to 5.99)	-0.2 (-0.27 to 0.15)	37,118 (30,231 to 43,419)	7.54 (6.29 to 8.91)	-0.06 (-0.2 to 0.09)	36,480 (25,653 to 49,748)	17.19 (12.07 to 23.31)	-0.44 (-0.53 to -0.34)
**High-income North America**	9,754 (8,361 to 11,230)	1.53 (1.32 to 1.77)	1.13 (0.92 to 1.32)	10,988 (8,872 to 13,360)	1.77 (1.43 to 2.15)	1.37 (0.98 to 1.79)	68,790 (54,760 to 85,626)	29.32 (23.41 to 36.37)	-0.09 (-0.2 to -0.06)
**North Africa and Middle East**	12,740 (9,255 to 17,062)	3.11 (2.31 to 4.09)	-0.03 (-0.29 to 0.35)	12,952 (9,358 to 17,369)	3.08 (2.28 to 4.07)	0.03 (-0.24 to 0.44)	322,058 (224,228 to 441,692)	70.9 (50.55 to 96.51)	-0.03 (-0.3 to -0.38)
**Oceania**	48 (32 to 69)	0.9 (0.62 to 1.23)	-0.09 (-0.25 to 0.12)	46 (31 to 66)	0.8 (0.55 to 1.1)	-0.09 (-0.26 to 0.12)	1145 (741 to 1666)	17.32 (11.62 to 24.59)	-0.12 (-0.28 to -0.09)
**South Asia**	9,905 (8,086 to 11,943)	0.78 (0.64 to 0.94)	-0.04 (-0.24 to 0.16)	9,249 (7,525 to 11,101)	0.7 (0.58 to 0.84)	-0.03 (-0.22 to 0.18)	228,543 (185,704 to 274,694)	16.16 (13.17 to 19.45)	-0.03 (-0.22 to -0.17)
**Southeast Asia**	9,709 (7,328 to 12,702)	1.84 (1.39 to 2.39)	0.03 (-0.15 to 0.26)	9,145 (6,792 to 12,022)	1.67 (1.25 to 2.17)	0.04 (-0.15 to 0.27)	208,013 (153,539 to 277,671)	34.99 (26.18 to 45.54)	-0.05 (-0.22 to -0.16)
**Southern Latin America**	739 (544 to 935)	0.87 (0.64 to 1.1)	0.5 (0.34 to 0.69)	681 (459 to 934)	0.8 (0.55 to 1.1)	0.53 (0.2 to 0.94)	14,000 (10,127 to 18,143)	16.77 (12.09 to 21.72)	0.45 (0.29 to 0.64)
**Southern Sub-Saharan Africa**	1,103 (905 to 1,315)	2.16 (1.8 to 2.57)	0.05 (-0.32 to 0.52)	1,036 (839 to 1,246)	1.96 (1.62 to 2.35)	0.05 (-0.33 to 0.53)	26,002 (20,921 to 31,675)	45.28 (36.64 to 54.58)	0.05 (-0.33 to -0.51)
**Tropical Latin America**	2,419 (2,115 to 2,702)	1.03 (0.9 to 1.15)	0.2 (0.13 to 0.28)	2,218 (1,939 to 2,486)	0.94 (0.82 to 1.05)	0.2 (0.14 to 0.29)	50,058 (43,578 to 56,548)	20.6 (17.92 to 23.21)	0.16 (0.09 to 0.23)
**Western Europe**	17,568 (14,479 to 20,788)	1.81 (1.49 to 2.15)	0.25 (0.17 to 0.33)	18,947 (15,193 to 23,282)	2.05 (1.63 to 2.55)	0.43 (0.25 to 0.65)	308,839 (250,962 to 370,796)	36.01 (28.98 to 43.6)	0.19 (0.11 to 0.27)
**Western Sub-Saharan Africa**	1,906 (1,372-2,462)	1.2 (0.88-1.51)	-0.11 (-0.26-0.08)	1,774 (1,268-2,303)	1.07 (0.78-1.37)	-0.32 (-0.46 to 0.14)	44,831 (31,310 to 58,275)	24.11 (17.12 to 31.46)	-0.13 (-0.29 to -0.04)

ASIR, age-standardize incidence rate; ASMR, age-standardize mortality rate; ASDR, age-standardize DALYs rate.

### Regional burden of LCDHC

**Figure 2:**
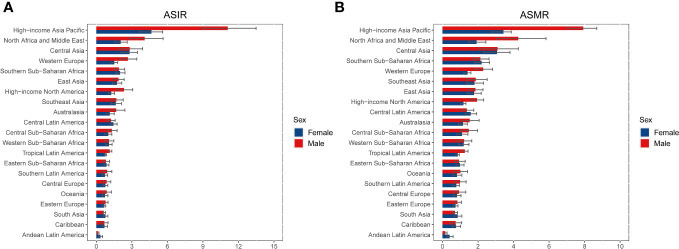
ASIR **(A)** and ASMR **(B)** of liver cancer due to hepatitis C in 2019 for 21 GBD regions, stratified by sex. ASIR, age-standardized incidence rate; ASMR, age-standardized mortality rate; GBD, Global Burden of Disease.

At the regional level, the highest number of new cases in 2019 was found in East Asia (34,877 [95%UI 28,701 to 41,388]), high-income Asia Pacific (27,961 [95%UI 30,944 to 23,725]), and Western Europe (17,568 [95%UI 14,479 to 20,788]) (**Table 1**, **Additional file 2: Figure S3**). In 2019, the ASIR of LCDHC per 100,000 population was found to be the highest in high-income Asia Pacific (7.54 [95%UI 6.29 to 8.91]), North Africa and the Middle East (3.08 [95%UI 2.28 to 4.07]), and Central Asia (2.81 [95%UI 2.04 to 3.63]). The percentage change in the ASIR varied across the 21 GBD regions from 1990 to 2019. Central Asia (169.88% [95%UI 128.49% to 218.50%]), Australasia (142.84% [95%UI 96.49% to 198.04%]), and high-income North America (137.35% [95%UI 98.03% to 179.23%]) exhibited the greatest increasing trends in ASIR of LCDHC, whereas East Asia (−60.17% [95%UI −68.27% to −50.38%]), the Caribbean (−51.36% [95%UI −58.69% to −43.00%]), and Central Europe (−42.39% [95%UI −50.50% to −33.50%]) exhibited the greatest decreasing trends in ASIR between 1990 and 2019 (**Table 1**, **Figure 2A**).

The highest number of death cases was observed in East Asia (34,878 [95%UI 28,702 to 41,388]), high-income Asia Pacific (27,963 [95%UI 23,725 to 30,944]), and Western Europe (17,568 [95%UI 14,479 to 20,788]) in 2019 (**Table 1**, **Additional file 2: Figure S4**). In 2019, the ASMR of LCDHC per 100,000 population was found to be the highest in high-income Asia Pacific (5.42 [95%UI 4.70 to 6.00]), North Africa and the Middle East (3.11 [95%UI 2.31 to 4.09]), and Central Asia (3.08 [95%UI 2.25 to 3.94]). From 1990 to 2019, Central Asia (174.59% [95%UI 133.51% to 224.48%]), Australasia (126.74% [95%UI 107.28% to 148.64%]), and high-income North America (113.39% [95%UI 91.98% to 131.77%]) exhibited the greatest increasing trends in ASMR, whereas East Asia (−63.77% [95%UI −70.79% to −54.85%]), the Caribbean (−51.93% [95%UI −59.26% to −43.81%]), and Central Europe (−44.84% [95%UI −53.03% to −36.18%]) exhibited the greatest decreasing trends in ASMR (**Table 1**, **Figure 2B**).

The highest number of DALY cases was observed in East Asia (751,414 [95%UI 609,844 to 900,126]), high-income Asia Pacific (438,136 [95%UI 381,839 to 484,032]), and North Africa and the Middle East (322,058 [95%UI 224,228 to 441,692]) in 2019 (**Table 1**, **Additional file 2: Figure S5**). In 2019, the ASDR of LCDHC per 100,000 population was found to be the highest in high-income Asia Pacific (98.19 [95%UI 109.03 to 87.14]), North Africa and the Middle East (70.9 [95%UI 50.55 to 96.51]), and Central Asia (64.25 [95%UI 45.62 to 83.96]). From 1990 to 2019, Central Asia (157.13% [95%UI 116.62% to 205.06%]), Australasia (117.86% [95%UI 98.76% to 140.19%]), and high-income North America (117.08% [95%UI 94.50% to 136.86%]) had the greatest increasing trends in ASDR, whereas East Asia (−65.96% [95%UI −72.87% to −57.65%]), the Caribbean (−52.06% [95%UI −59.85% to −43.35%]), and Central Europe (−43.84% [95%UI −52.51% to −34.23%]) had the greatest decreasing trends in ASDR (**Table 1**, **Additional file 2: Figure S6**).

### National burden of LCDHC

**Figure 3:**
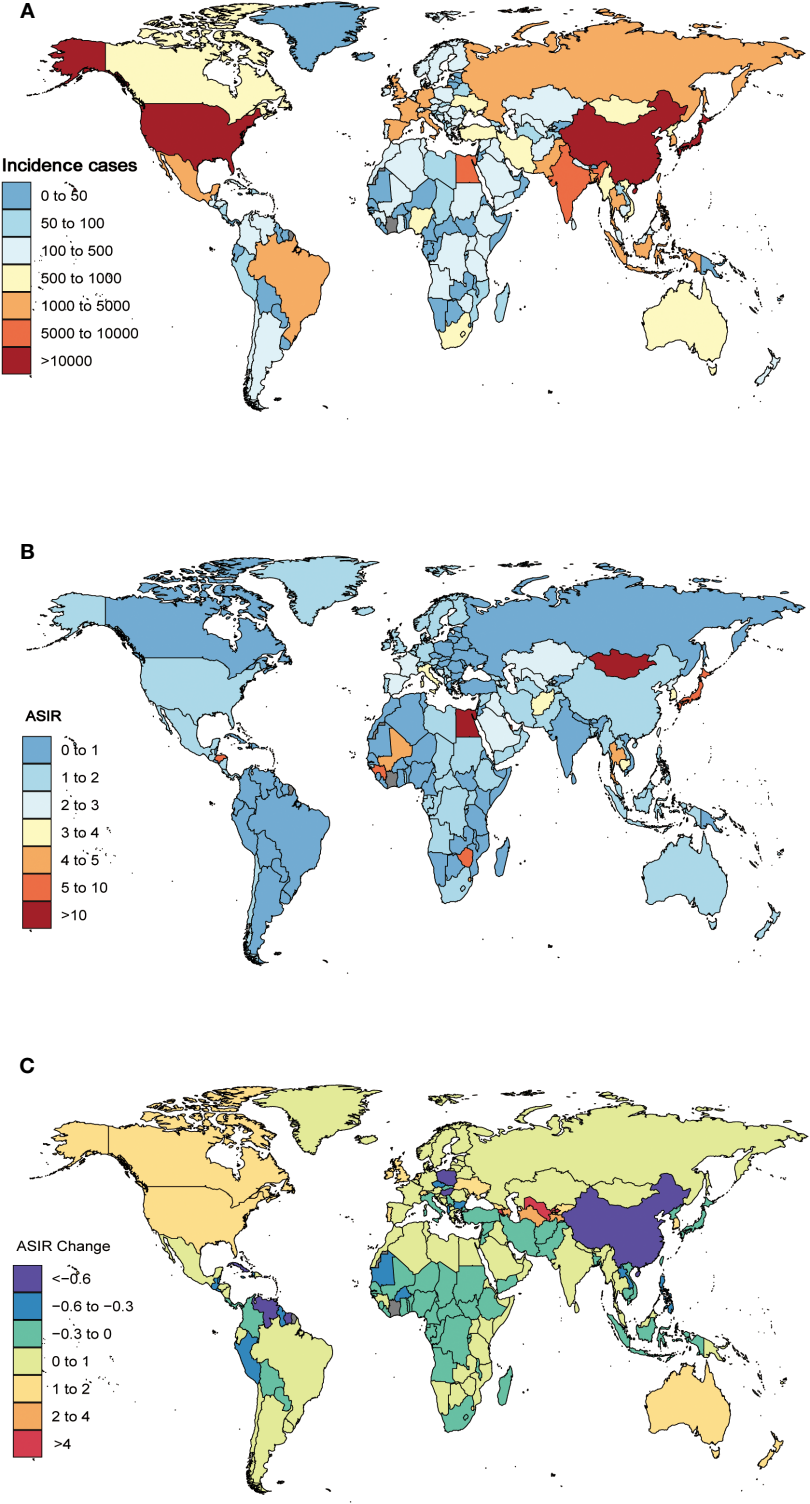
The global incidence cases **(A)** and ASIR **(B)** of liver cancer due to hepatitis C per 100,000 population in 2019, and the relative change in ASIR of liver cancer due to hepatitis C between 1990 and 2017 **(C)**, stratified by country and territory. ASIR, age-standardized incidence rate.

At the national level, China, Japan, and the United States had the highest incidence and deaths in 2019. China had 34,036 (95%UI 27,796 to 40,828) incident cases and 33,079 (95%UI 27,212 to 39,258) deaths, Japan had 33,311 (95%UI 26,824 to 39,444) incident cases and 25,051 (95%UI 21,085 to 27,539) deaths, and United States had 10,408 (95%UI 8,343 to 12,654) incident cases and 9,231 (95%UI 7,939 to 10,567) deaths (**Additional file 1: Table S1**, **Figures 3A**, **S7**). In 2019, China had the highest number of DALYs of 714,427 (95%UI 580,678 to 856,767), followed by Japan (383,625 [95%UI 337,521 to 418,060]) (**Additional file 1: Table S3**, **Additional file 2: Figure S8**).

At the national level, the ASIR of LCDHC varied from 0.11 to 35.02 per 100,000 population. Mongolia (35.02 [95%UI 24.73 to 46.77]), Egypt (13.64 [95%UI 9.44 to 19.19]), and Japan (8.60 [95%UI 7.06 to 10.25]) had the three highest ASIR in 2019, whereas Cameroon (0.11 [95%UI 0.07 to 0.16]), Niger (0.13 [95%UI 0.08 to 0.18]), and Peru (0.19 [95%UI 0.11 to 0.30]) exhibited the lowest ASIR (**Additional file 1: Table S1**, **Figure 3B**). The percentage changes in the ASIR from 1990 to 2019 differed substantially among 204 countries and territories. Cabo Verde (835.11% [95%UI 612.64% to 1,163.27%]), Uzbekistan (569.96% [95%UI 454.14% to 698.52%]), and Armenia (521.36% [95%UI 408.14% to 645.60%]) had the greatest increasing trends in ASIR over the 30 years. In contrast, Poland (−77.32% [95%UI −80.83% to −73.05%]), Saint Kitts and Nevis (−72.57% [95%UI −77.28% to −66.73%]), and Guatemala (−72.14% [95%UI −77.51% to −65.03%]) had the greatest decreasing trends in ASIR from 1990 to 2019 (**Additional file 1: Table S1**, **Figure 3C**).

The ASMR of LCDHC varied from 0.12 to 40.31 per 100,000 population. In 2019, Mongolia (40.31 [95%UI 28.58 to 53.28]), Egypt (14.05 [95%UI 9.83 to 19.71]), and Honduras (6.82 [95%UI 2.82 to 10.85]) had the highest ASMR. In contrast, Cameroon (0.12 [95%UI 0.08 to 0.18]), Niger (0.14 [95%UI 0.09 to 0.20]), and Peru (0.22 [95%UI 0.13 to 0.34]) had the lowest ASMR (Additional file 1: **Table S2**, **Additional file 2: Figure S9**). The highest increases in ASMR were observed in Cabo Verde (850.20% [95%UI 625.80% to 1,193.75%]), Uzbekistan (559.52% [95%UI 449.52% to 685.60%]), and Armenia (522.62% [95%UI 409.84% to 643.88%]). The highest decreases ASMR during this period were found in Poland (−77.89% [95%UI −81.36% to −74.15%]), Bermuda (−73.89% [95%UI −78.94% to −67.63%]), and Saint Kitts and Nevis (−73.01% [95%UI −77.49% to −67.48%]) (**Additional file 1: Table S2**, **Additional file 2: Figure S10**).

In addition, the ASDR of LCDHC in 2019 ranged from 2.45 to 752.55 per 100,000 population. The highest rates were found in Mongolia (752.55 [95%UI 511.89 to 1,044.07]), Egypt (333.10 [95%UI 224.95 to 473.10]), and Honduras (135.41 [95%UI 54.48 to 221.81]), and the lowest rates were found in Cameroon (2.45 [95%UI 1.49 to 3.67]), Niger (2.87 [95%UI 1.77 to 4.21]), and Peru (3.83 [95%UI 2.24 to 6.36]) (**Additional file 1: Table S3**, **Additional file 2: Figure 11**).

The highest increases in the ASDR of LCDHC between 1990 and 2019 were in Cabo Verde (803.22% [95%UI 587.82% to 1,112.24%]), Uzbekistan (595.69% [95%UI 471.46% to 736.68%]), and Armenia (498.69% [95%UI 391.95% to 624.60%]). In contrast, Poland (−77.73% [95%UI −81.44% to −73.69%]), Bermuda (−75.14% [95%UI −80.18% to −68.49%]), and Saint Kitts and Nevis (−73.37% [95%UI −78.26% to −67.41%]) exhibited the highest decreases in ASMR from 1990 to 2019 (**Additional file 1: Table S3**, **Additional file 2: Figure S12**).

### Burden of LCDHC by age and sex

**Figure 4:**
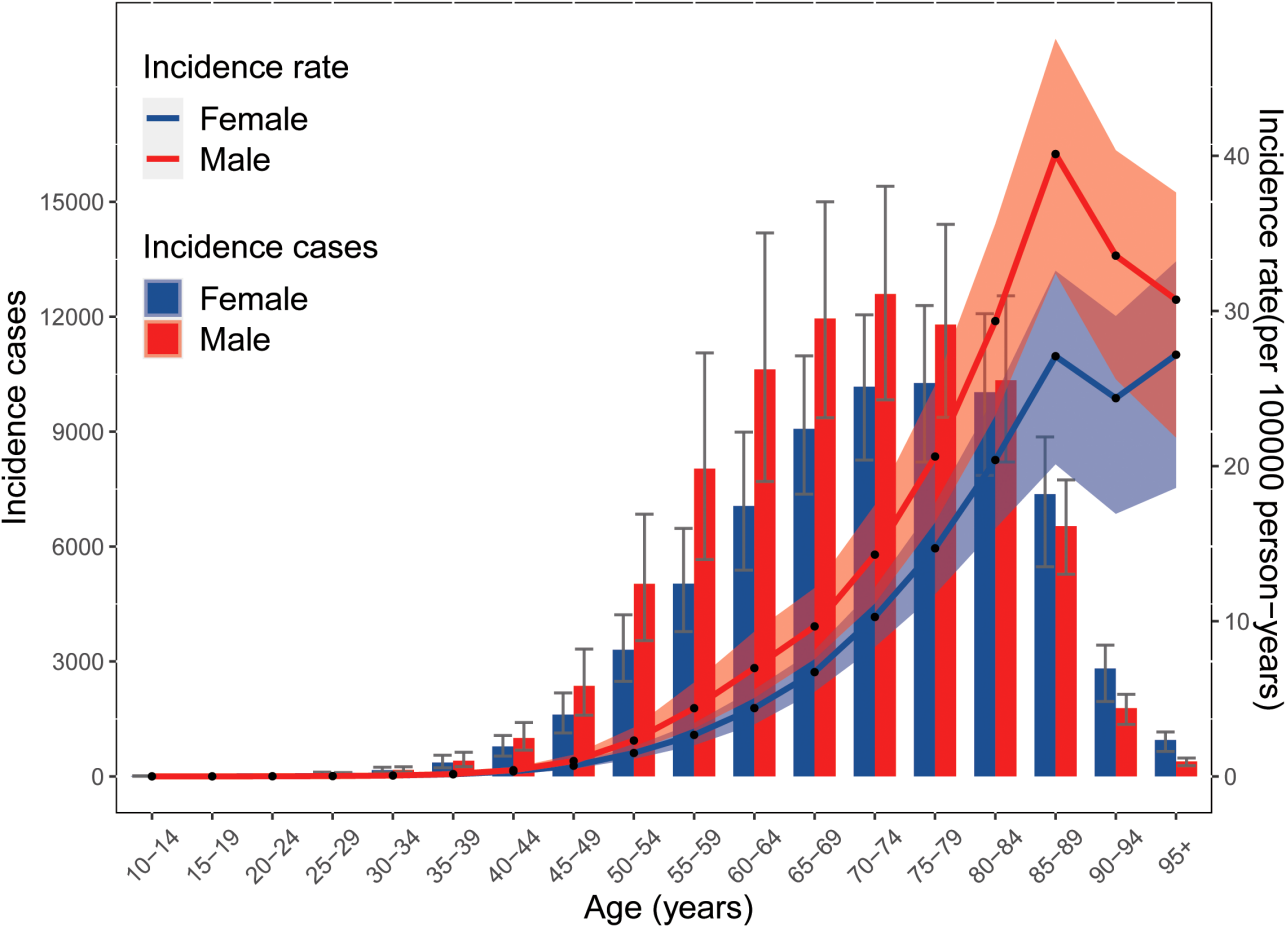
Global number and rates of incidence for liver cancer due to hepatitis C per 100,000 population, stratified by age and sex in 2019. Shading indicates the 95%UIs for the incidence rate. UIs, uncertainty intervals.

Globally, the incidence rates of LCDHC were higher in men and increased with age, with a peak incidence in the 95+ age group for women and the 85–89 age group for men in 2019. The number of incident cases increased with age and peaked in the 70–74 age group for men and the 75–79 age group for women in 2019, followed by a decreasing trend (**Figure 4**). Before the age of 70–74 years, incidence rates were higher in men than in women, but after this age, the incidence rate in women surpassed that of men. No statistically significant difference was found in the incidence rates between men and women in any age group (**Figure 4**). The pattern of DALYs due to LCDHC by sex and age group was similar to that of incidence (**Additional file 2: Figure S13**).

There was no statistically significant difference found in the mortality rates of LCDHC between men and women in any age group. In 2019, the global mortality rates of liver cancer were higher in men and increased with age for both women and men. The number of death cases increased with age and peaked in the 70–74 age group for men and the 80–84 age group for women in 2019, followed by a decrease in the mortality rates of LCDHC. Before the age of 80–84 years, the mortality rates of LCDHC were higher in men than in women, but after this age group, the mortality rate in women surpassed that of men (**Additional file 2: Figure S14**).

### Burden of LCDHC by sociodemographic index

**Figure 5:**
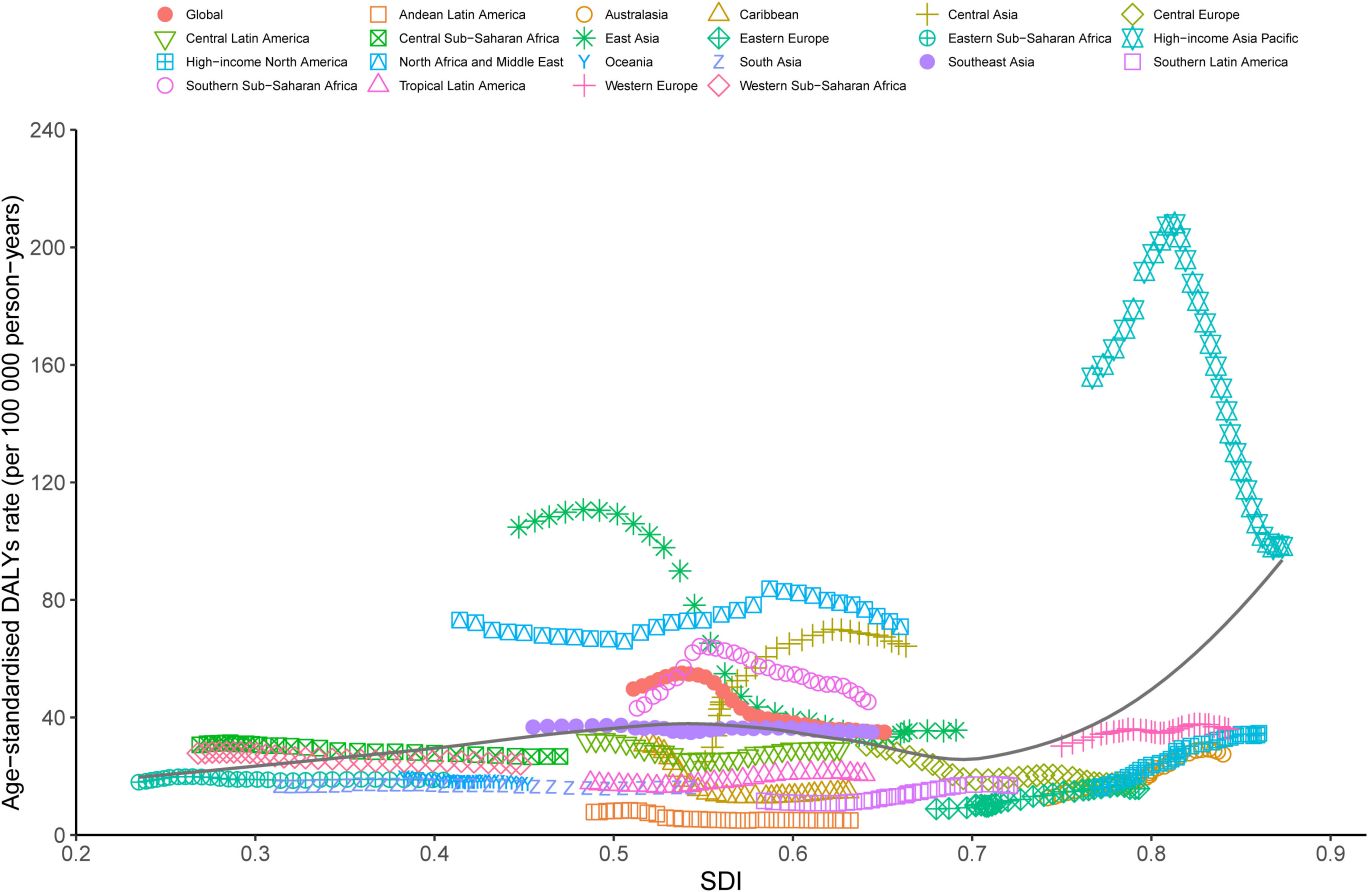
Trends in ASDR of liver cancer due to hepatitis C across 21 Global Burden of Disease study regions are illustrated by sociodemographic index (SDI) for both sexes combined from 1990 to 2019. The black line indicates expected values. DALYs, disability-adjusted life years; SDI, sociodemographic index; ASDR, age-standardized DALY rate.

At the regional level, a positive association was observed between the ASDR of LCDHC and the SDI from 1990 to 2019. The lowest ASDR was observed at an SDI of approximately 0.569, with an overall increasing trend observed with increasing SDI value. From 1990 to 2019, the observed burden was higher than the expected level based on SDI in high-income Asia Pacific, East Asia, North Africa and the Middle East, Global, Southern Sub-Saharan Africa, and Central Asia. By contrast, Andean Latin America, Tropical Latin America, Western Europe, South Asia, the Caribbean, Central Europe, Eastern Europe, high-income North America, Australasia, and Central Latin America were below the expected level based on the SDI in all years (**Figure 5**). The association between incidence, death, and SDI is described in the additional file (**Additional file 2: Figures S15**, **S16**).

**Figure 6:**
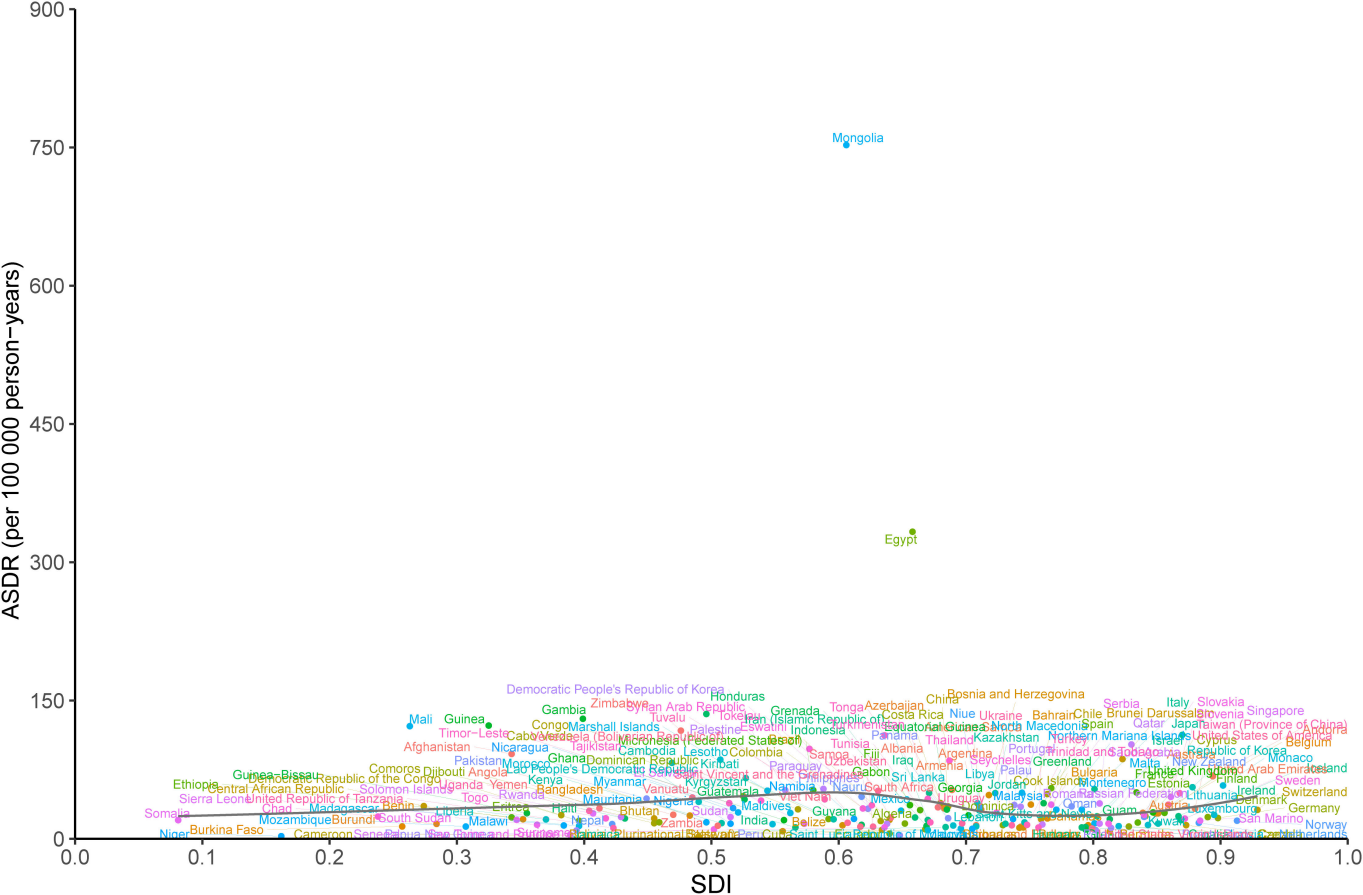
ASDR of liver cancer due to hepatitis C in 204 countries and territories and SDI in 2019. The black line indicates the expected values based on sociodemographic index and disease rates in all locations. DALYs, disability-adjusted life years; SDI, sociodemographic index; ASDR, age-standardized DALY rate.

At the national level, there was a nonlinear association between the ASDR of LCDHC and SDI in 2019. The expected values exhibited a slight increase and then decreasing and increasing trends in the SDI. The burden due to LCDHC was higher than the expected levels based on SDI in countries such as Mongolia, Egypt, and Honduras, whereas in countries such as Barbuda, Niger, and Cameroon, the burden was much lower than the expected levels (**Figure 6**). The ASIR and ASMR exhibited a similar pattern with ASDR (**Additional file 2: Figures S17**, **S18**).

## Discussion

In the present study, we reported the incidence, mortality, and DALY numbers and ASRs of LCDHC at the global, regional, and national levels over 30 years from 1990 to 2019 based on the GBD 2019 study. In 2019, there were 152,225 incident cases, 141,810 mortality cases, and 2,878,024 DALY cases, and these numbers increased from 1990 to 2019. The ASRs for incidences, mortalities, and DALYs exhibited a global upward trend during this period. To the best of our knowledge, this study presents the first assessment of the correlation between the ASR and the SDI in 21 GBD regions and 204 countries. We found that the ASDR of LCDHC increased with increasing SDI in terms of region, whereas there was no correlation between ASDR and SDI in terms of countries.

Previous studies reported that hepatitis B and hepatitis C are the leading causes of liver cancer (4), with hepatitis B responsible for approximately 41% of liver cancer deaths and hepatitis C for 28.5% in 2019 (4). However, the proportion of liver cancer deaths attributed to these viruses was only 18.7% in 2016 (13). The incidence of hepatitis B–related liver cancer has decreased somewhat in recent years because of the widespread use of hepatitis B vaccines worldwide (14, 15), but the incidence of hepatitis C–related liver cancer has been increasing because of a lack of effective vaccines (16, 17). The burden of acute hepatitis B has been decreasing, whereas that of acute hepatitis C has remained stable from 1990 to 2019 (17). As a result, hepatitis C has become one of the most significant causes of liver cancer (13, 18). Understanding the burden of liver cancer due to hepatitis C infection may help reduce the overall burden of the disease. Our study found that the number of incident cases due to LCDHC increased, whereas the ASIR of LCDHC decreased from 1990 to 2019, consistent with previous research (4). The highest age-standardized incidence rates of liver cancer due to chronic hepatitis C infection in 2019 were observed in high-income regions of the Asia Pacific, North Africa and the Middle East, and Central Asia, and these regions also exhibited the greatest increasing trends in the ASIR of LCDHC from 1990 to 2019. However, in 2017, Central Asia, high-income Asia Pacific, and East Asia had the highest ASIR (19). HCV is the leading cause of primary liver cancer in the high SDI regions, with liver cancer due to chronic hepatitis C infection accounting for 43% and 41.4% of the total liver cancer deaths and incident cases, respectively, in these regions (19). Our investigation reveals that certain high-income regions, such as Japan, had a higher ASIR of LCDHC than most countries. Despite having more resources, including financial resources, healthcare infrastructure, and medical technology, high-income countries are not immune to the burden of HCV infection and LCDHC. Effective screening and early detection programs for LCDHC can help reduce the incidence of LCDHC. However, only 9 of 45 high-income countries are expected to achieve the HCV elimination goal by 2030, with 30 of these countries projected to reach the goal after 2050 (20). Notably, the three countries with the highest ASIR of LCDHC in 2017 were Mongolia, Egypt, and Japan (19). In 2019, the same countries continued to have the highest ASIR of LCDHC, indicating that the burden of LCDHC is not limited to developed countries and that developing countries also have a high incidence of this disease. Consequently, it is imperative to allocate more resources and implement preventative measures aimed at reducing the burden of LCDHC in these regions.

Unsafe injections, shared injection apparatuses, blood transfusions, and mother-to-child transmission remain the primary modes of HCV transmission, whereas vertical transmission is the primary route of infection in children (21, 22). According to a previous study, approximately 5% of HCV-infected individuals (315,000 people) were infected through unsafe injections in 2015 (23). Preventing the reuse of syringes can significantly reduce the incidence of HCV infections caused by unsafe injections (24). In addition, dental procedures, tattoos, and manicure and pedicure services are high-risk factors for HCV infection (25, 26). Therefore, preventing the widespread transmission of HCV among high-risk individuals is an important strategy to reduce the burden of LCDHC. To date, no effective vaccine exists to prevent HCV infection. Although direct-acting antiviral agent regimens were introduced in 2014 and can cure more than 90% of HCV patients, high costs, drug resistance, and reinfection rates are still significant obstacles to achieving this goal (27, 28). Therefore, many HCV patients lack efficacy treatment and then progress to liver cancer. With the improvement in the world’s medical health standards and the popularization of HCV health knowledge, the detection rate of LCDHC has increased, which has led to a decrease in ASIR. Nonetheless, the development of an effective HCV vaccine is imperative to achieving the goal of HCV elimination by 2030 (29).

The burden of LCDHC exhibits variations across different age groups and sex. The incidence rates of LCDHC were generally higher in men than women, with this trend continuing until the age of 85–89 age group. The reason why the rates are higher in men than women may be as follows: men are more likely to engage in high-risk behaviors that can lead to HCV infection, such as injection drug use or unprotected sex with multiple partners. Men are also more likely to have jobs that expose them to blood and bodily fluids, such as healthcare workers or emergency responders (30). Hormonal differences between men and women may also play a role, as estrogen has been shown to have a protective effect against liver cancer, with women generally having higher estrogen levels than men (31). Moreover, studies have suggested that testosterone may promote the growth of hepatocellular carcinoma cells, which could also contribute to the sex disparity in HCV-related hepatocellular carcinoma incidence (32). In 2017, the highest burden of liver cancer was found in the above 50 age group, and liver cancer due to hepatitis C and alcohol use contributes to approximately 95% of the proportions (19). In our study, we found that the number of incident cases was high in those aged 50–89 years. The findings of our study indicate that screening for HCV is recommended for older individuals to facilitate early detection of the infection and prompt initiation of treatment. HCV-infected older individuals should receive timely and effective treatment to decrease the risk of developing liver cancer. Furthermore, routine monitoring of liver function and early detection of any signs of liver cancer should be conducted regularly in older people with HCV.

Previous studies have investigated the relationship between incidence, death, and DALYs with the SDI of countries and regions for liver cancer. However, this study is the first to examine the correlation between the burden of LCDHC and the SDI of regions and countries as regards incidence, death, and DALYs. Our findings reveal a positive association between the burden of LCDHC and SDI values from 1990 to 2019. Moreover, a nonlinear association was found between the burden of LCDHC and the SDI value of countries in 2019. Although there is a general association between the burden of LCDHC and SDI, it should not be considered in isolation. The burden of LCDHC is not limited to developed or less-developed regions or countries, and a relatively high burden of LCDHC was observed in regions or countries with a range of SDI values. Furthermore, to assess the effectiveness of prevention programs, the observed value and expected levels in each country and region should be compared. It is crucial to consider other factors such as the prevalence of hepatitis C infection, accessibility to healthcare and screening programs, the availability of effective treatment options, demographics, lifestyle factors, and socioeconomic and environmental factors.

In the present study, we analyzed data from 204 countries and territories from 1990 to 2019 to provide a high-quality assessment of global and regional burdens and trends for LCDHC. However, some limitations should be considered. First, this study was a secondary analysis of data from the GBD study, and as with many GBD studies, the accuracy and reliability of estimates largely depend on the quality and quantity of input data used for modeling. Second, some HCV infections may occur simultaneously with other liver diseases such as HBV infection (25, 33, 34), alcoholic liver disease (35), or diabetes (36), making it difficult to determine the true cause of liver cancer, consequently leading to potential data distortion. Third, the effects of prevention and management strategies in different countries were not taken into account, and significant variations may exist between low- to middle-income countries and high-income countries.

## Conclusion

Although the ASR of LCDHC has been decreasing, the absolute numbers of incident cases, mortality, and DALYs have increased, indicating that LCDHC remains a significant global burden. Geographically, the burden of LCDHC exhibits significant variations, with high-income Asia Pacific, North Africa and the Middle East, and Central Asia having the highest burden among the 21 GBD regions. Furthermore, male and older individuals have a higher burden of LCDHC. Our findings provide insight into the global burden trend of LCDHC and could assist policymakers in establishing appropriate methods for achieving the HCV elimination target by 2030 and reducing the burden of LCDHC. It is crucial to focus on these burden trends, particularly as the number of LCDHC cases and the aging population continue to rise.

## Data availability statement

The datasets presented in this study can be found in online repositories. The names of the repository/repositories and accession number(s) can be found in the article/**Supplementary Material**.

## Author contributions

GO and JW, YZ and YW analyzed the data and edited the manuscript. GH, JW, YW, SL, and JL collected and interpreted the data. JW, GY, and SH designed the study, interpreted the data, and revised the draft. GY and SH provided financial support. All authors contributed to the article and approved the submitted version.
