# Supplementary material for: Burden of liver cancer due to hepatitis C from 1990 to 2019 at the global, regional, and national levels
Source: Front Oncol. 2023 Dec 19;13:1218901. doi: 10.3389/fonc.2023.1218901 (PMC10760495; doi:10.3389/fonc.2023.1218901)
Supplement: Supplementary file 2 [file DataSheet_2.docx]

Additional File 2: Supplementary Figures

Global Burden of Liver cancer due to hepatitis C from 1990 to 2019 at the global, regional, and national levels

Jie Wei^1,2,3,#^, Guoqing Ouyang^1,2,3#^, Guozhen Huang^1,2,3^, Yong Wang^1,2,3^, Shuangjiang Li^1,2,3^, Jiaping Liu^1,2,3^, Yanhong Zhang^4^, Guandou Yuan^1,2,3,^*, Songqing He^1,2,3,^*

^1^Division of Hepatobiliary Surgery, The First Affiliated Hospital of Guangxi Medical University, Nanning, Guangxi, China;

Key Laboratory of Early Prevention and Treatment for Regional High Frequency Tumor (Guangxi Medical University), Ministry of Education, Nanning, Guangxi, China;

^3^Guangxi Key Laboratory of Immunology and Metabolism for Liver Diseases, Nanning (Guangxi Medical University), Nanning, Guangxi, China;

^4^Comparative Oncology Laboratory, Schools of Veterinary Medicine and Medicine, University of California at Davis, Davis, CA, 95616, USA.

**# Theses authors contributed equally as first author**

***Correspondence：**

Songqing He, Division of Hepatobiliary Surgery, The First Affiliated Hospital of Guangxi Medical University, NO 6 Shuangyong Road, Nanning, Guangxi 530021, China. Email: dr_hesongqing@163.com

Guandou Yuan, Division of Hepatobiliary Surgery, The First Affiliated Hospital of Guangxi Medical University, NO 6 Shuangyong Road, Nanning, Guangxi 530021, China. E-mail: dr_yuangd@gxmu.edu.cn

**Contents**

[Fig S1: The DAYLs number is illustrated for liver cancer due to hepatitis C at the global and regional levels from 1990 through 2019. 2](#_Toc14175)

[Fig S2: The DAYLs ASR is illustrated for liver cancer due to hepatitis C at the global and regional levels from 1990 through 2019. 3](#_Toc12064)

[Fig S3: The incidence cases of liver cancer due to hepatitis C in 2019 for 21 GBD regions, by sex. 4](#_Toc25167)

[Fig S4: The mortality cases of liver cancer due to hepatitis C in 2019 for 21 GBD regions, by sex 5](#_Toc8732)

[Fig S5: The DALYs cases of liver cancer due to hepatitis C in 2019 for 21 GBD regions, by sex 6](#_Toc7323)

[Fig S6: The age-standardized DALYs rate of liver cancer due to hepatitis C in 2019 for 21 GBD regions, by sex. 7](#_Toc28059)

[Fig S7: The global mortality cases of liver cancer due to hepatitis C per 100,000 population in 2019, by country and territory. 8](#_Toc11919)

[Fig S8: The global DALYs cases of liver cancer due to hepatitis C per 100,000 population in 2019, by country and territory. 9](#_Toc12168)

[Fig S9: The global ASMR of liver cancer due to hepatitis C per 100,000 population in 2019, by country and territory. ASMR indicates Age-standardized motality rate. 10](#_Toc531)

[Fig S10: The global mortality ASR change of liver cancer due to hepatitis C per 100,000 population in 2019, by country and territory. ASMR indicates Age-standardized mortality rate. 11](#_Toc26433)

[Fig S11: The global DALYs ASR of liver cancer due to hepatitis C per 100,000 population in 2019, by country and territory. ASDR indicates Age-standardized DALYs rate. 12](#_Toc18871)

[Fig S12: The global DALYs ASR chance of liver cancer due to hepatitis C per 100,000 population in 2019, by country and territory. ASDR indicates Age-standardized DALYs rate. 13](#_Toc13057)

[Fig S13: Global DALYs rate and DALYs cases of liver cancer due to hepatitis C per 100,000 population by age and sex, 2019. 14](#_Toc12563)

[Fig S14: Global mortality rate and mortality cases of liver cancer due to hepatitis C per 100,000 population by age and sex, 2019. 15](#_Toc1337)

[Fig S15: Age-standardized incidence rates of liver cancer due to hepatitis C in 21 GBD regions by SDI, 1990–2019. 16](#_Toc26917)

[Fig S16: Age-standardized mortality rates of liver cancer due to hepatitis C in 21 GBD regions by SDI, 1990–2019. 17](#_Toc31229)

[Fig S17: Age-standardized incidence rates for liver cancer due to hepatitis C by 204 countries and territories and SDI, 2019. 18](#_Toc14477)

[Fig S18: Age-standardized mortality rates for liver cancer due to hepatitis C by 204 countries and territories and SDI, 2019. 19](#_Toc29891)


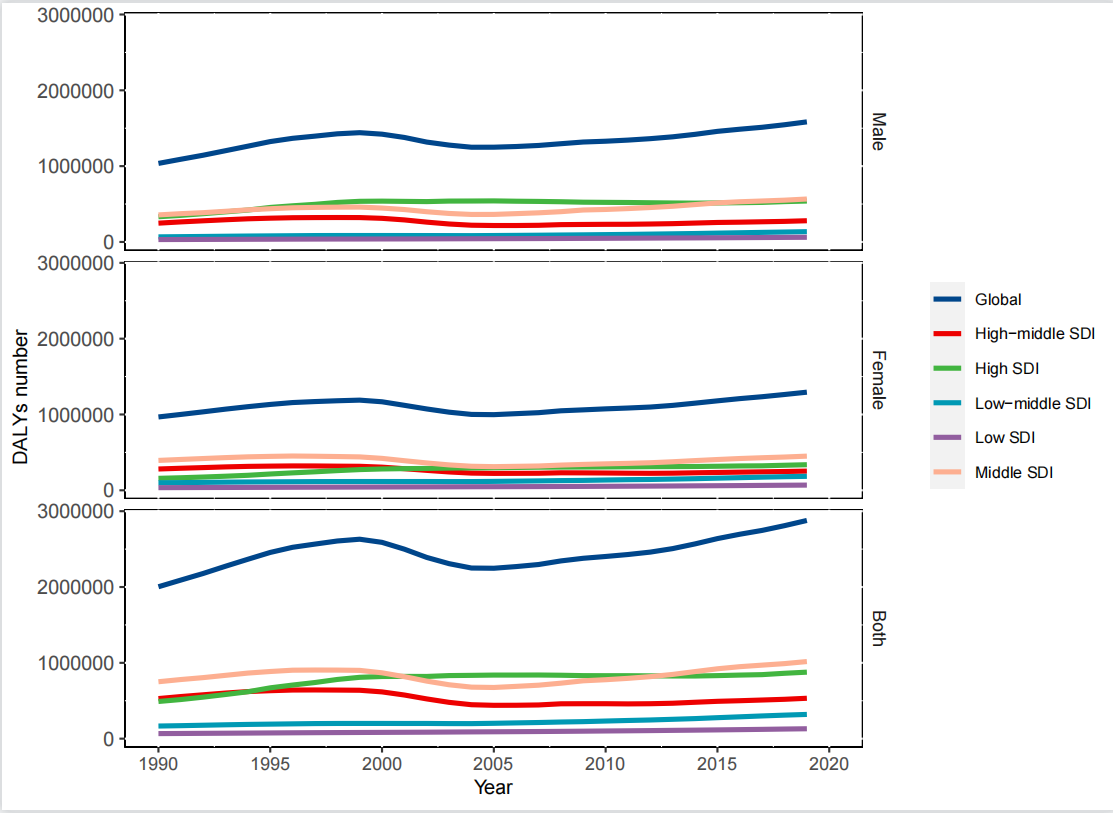


**Fig S1**: The DAYLs number is illustrated for liver cancer due to hepatitis C at the global and regional levels from 1990 through 2019.


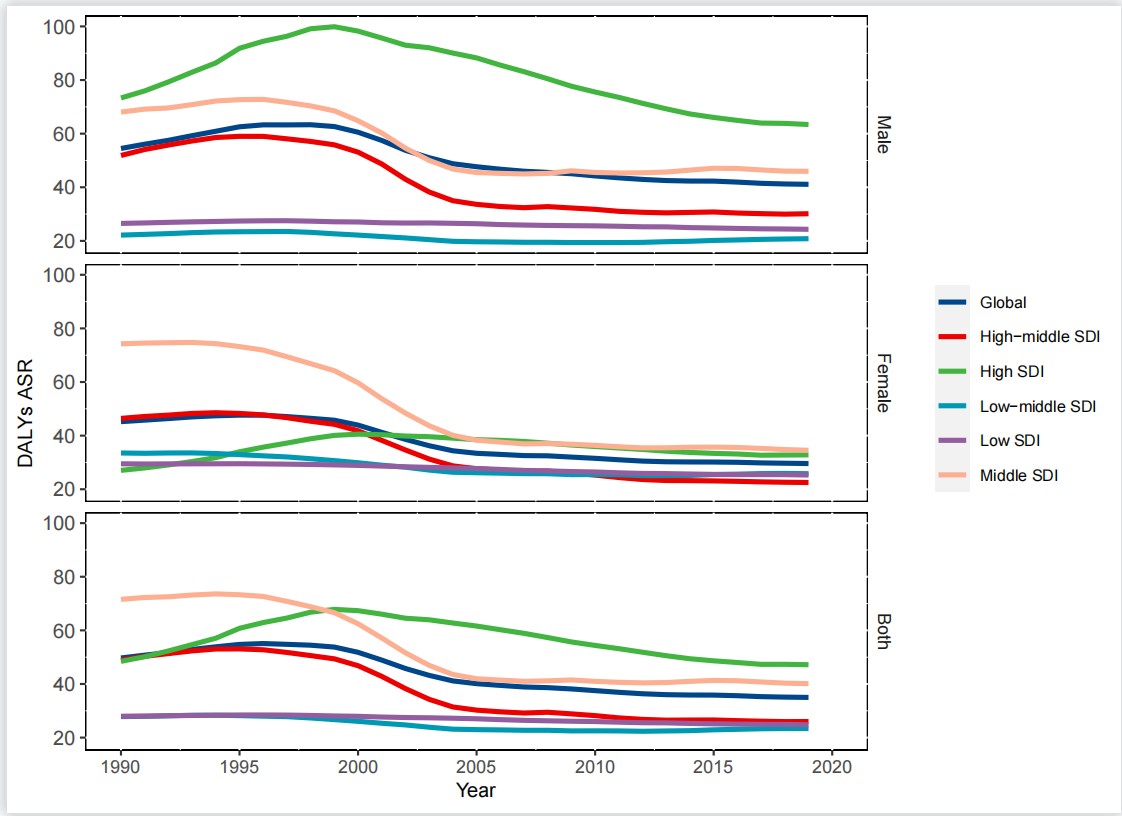


**Fig S2**: The DAYLs ASR is illustrated for liver cancer due to hepatitis C at the global and regional levels from 1990 through 2019.


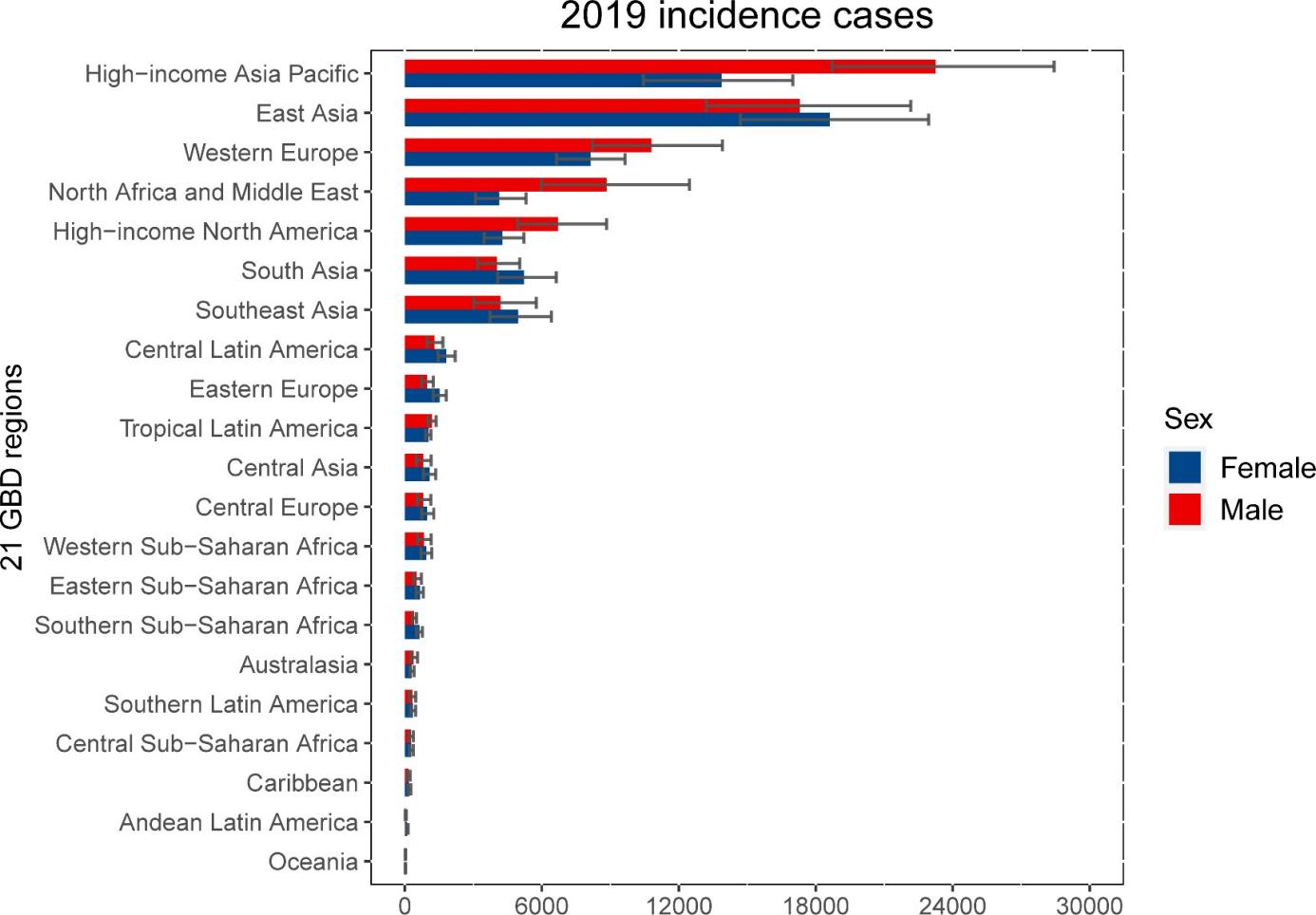


**Fig S3**: The incidence cases of liver cancer due to hepatitis C in 2019 for 21 GBD regions, by sex.


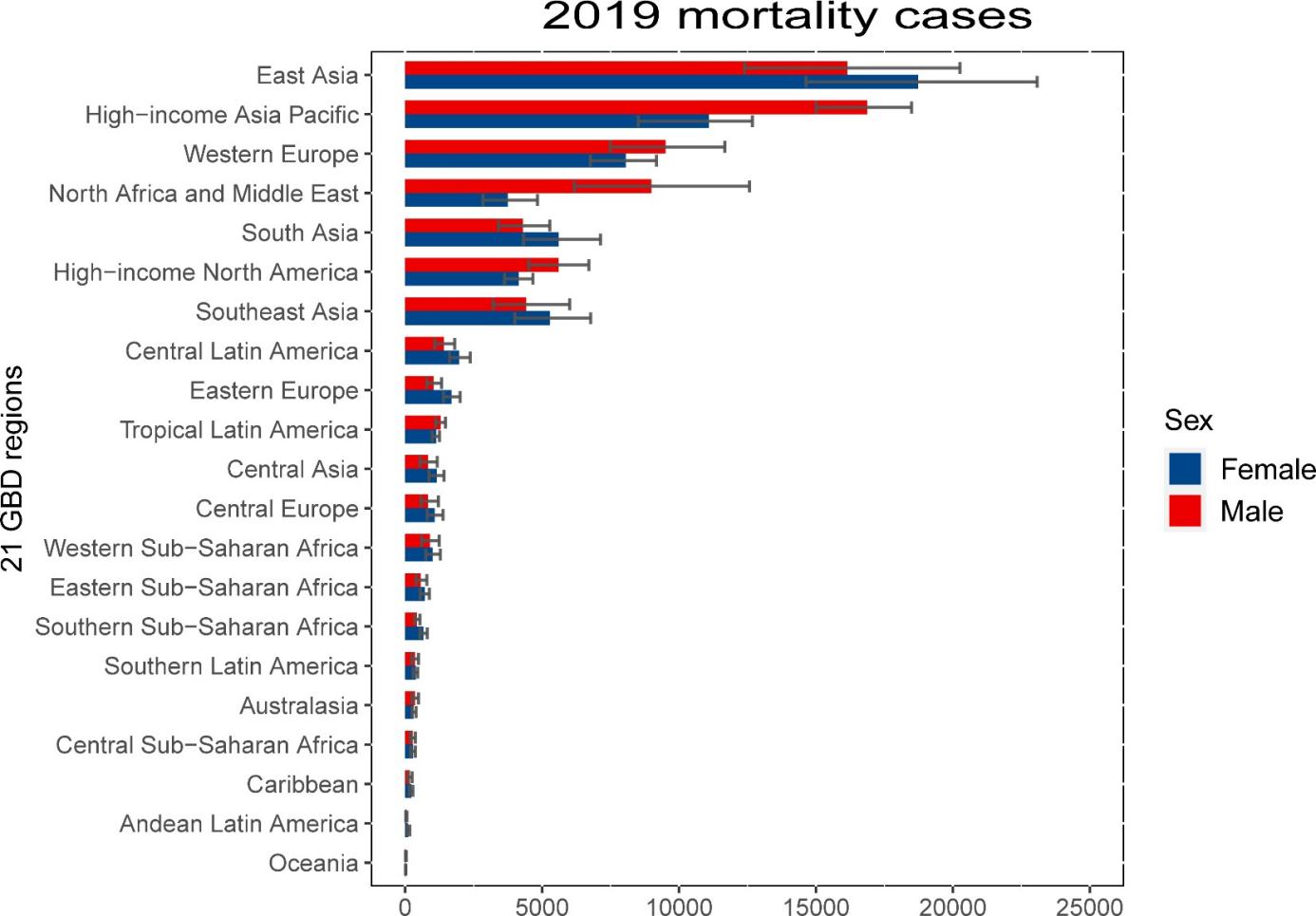


**Fig S4**: The mortality cases of liver cancer due to hepatitis C in 2019 for 21 GBD regions, by sex


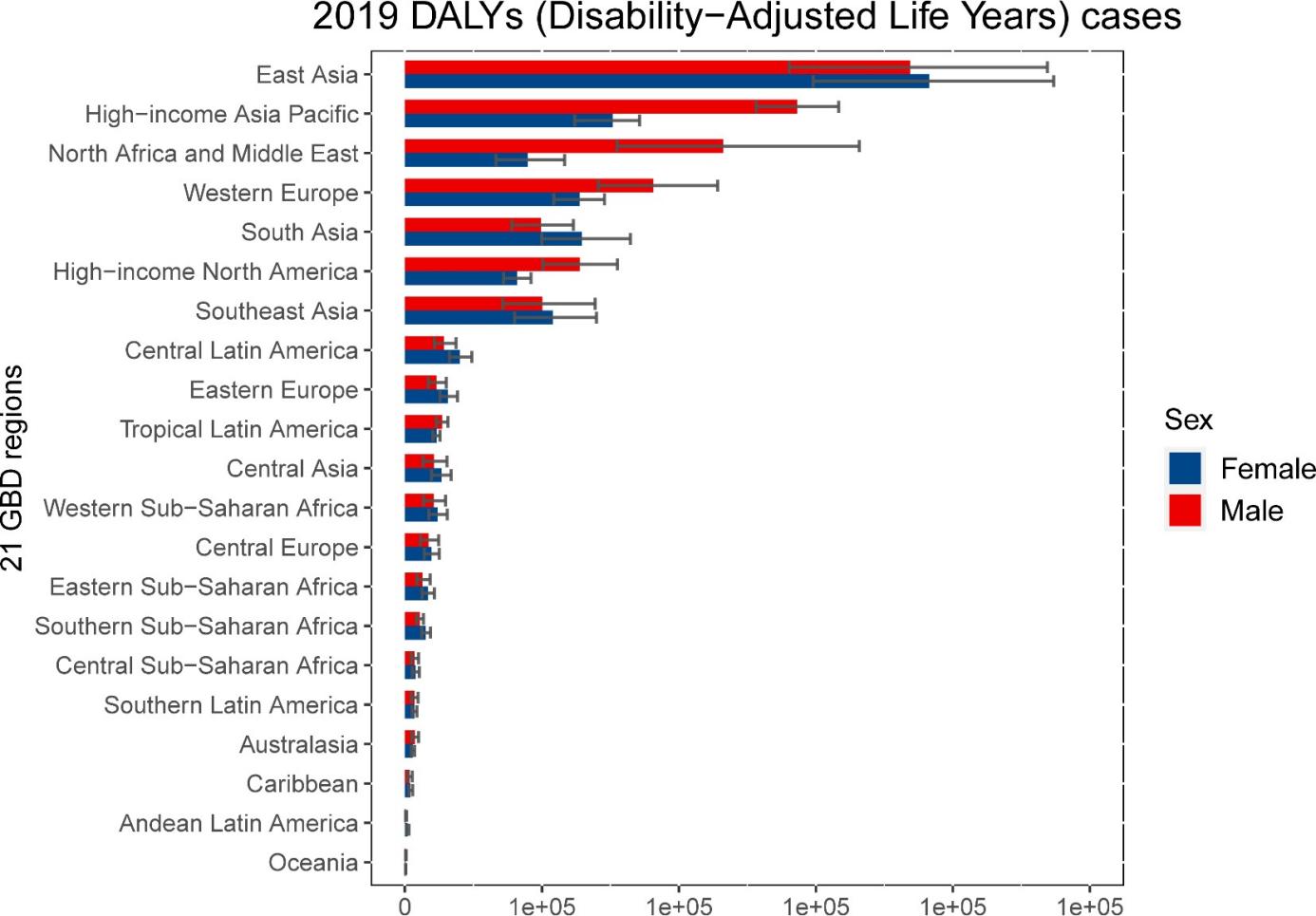


**Fig S5**: The DALYs cases of liver cancer due to hepatitis C in 2019 for 21 GBD regions, by sex


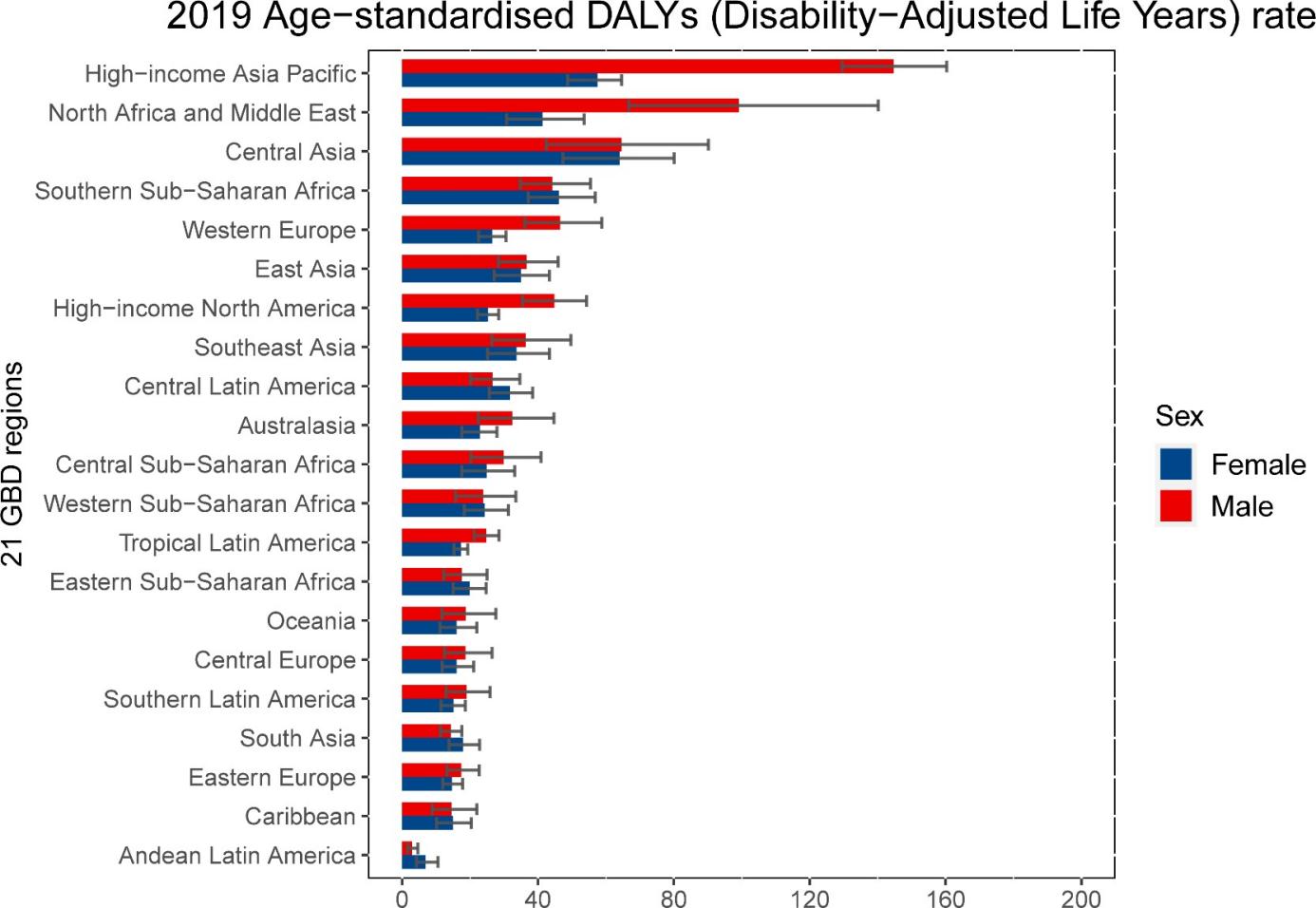


**Fig S6**: The age-standardized DALYs rate of liver cancer due to hepatitis C in 2019 for 21 GBD regions, by sex.


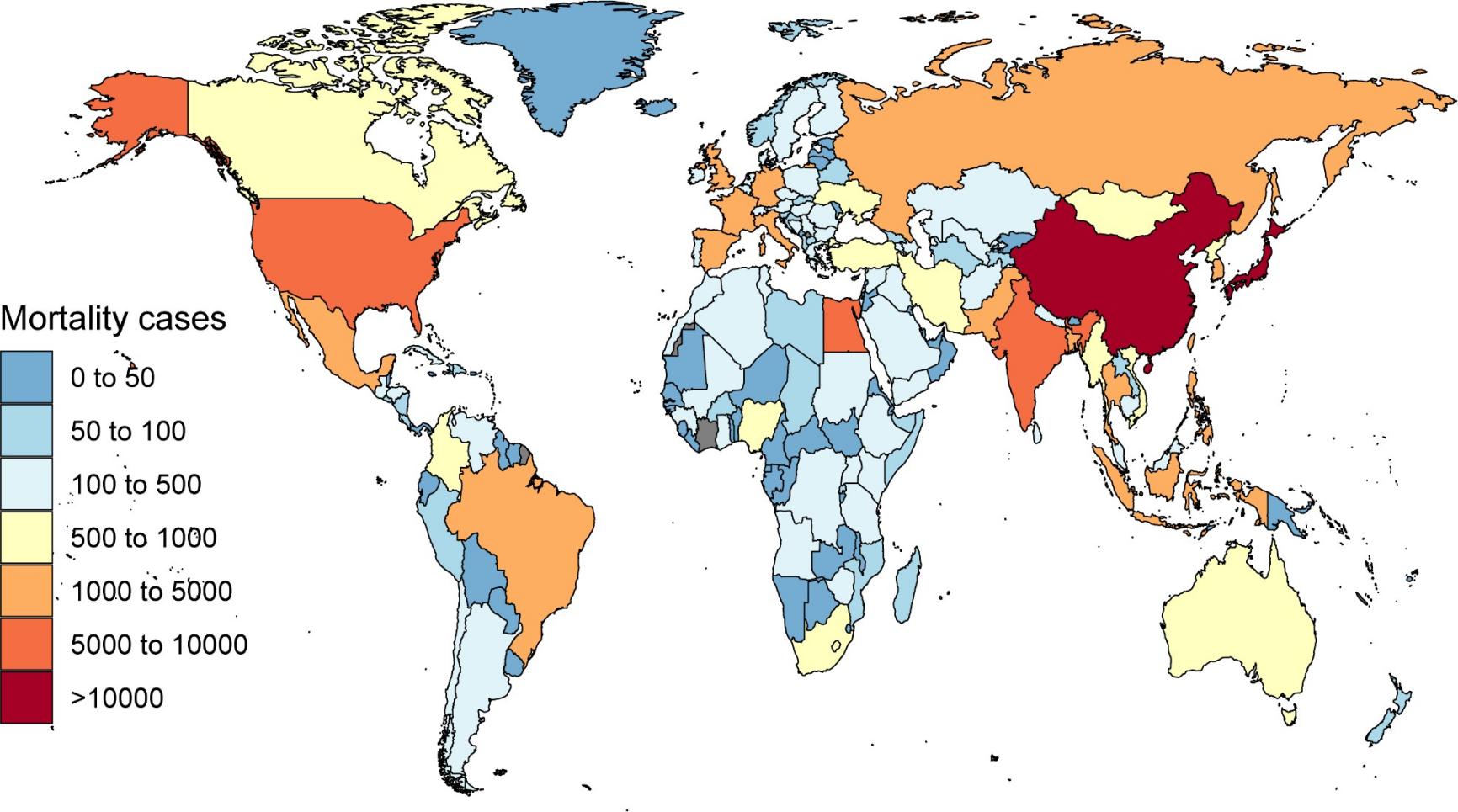


**Fig S7:** The global mortality cases of liver cancer due to hepatitis C per 100,000 population in 2019, by country and territory.


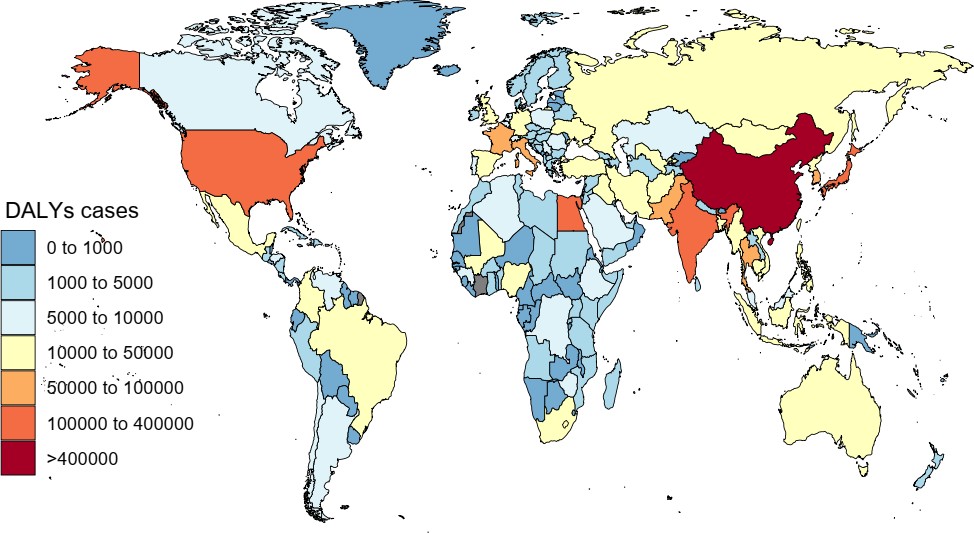


**Fig S8**: The global DALYs cases of liver cancer due to hepatitis C per 100,000 population in 2019, by country and territory.


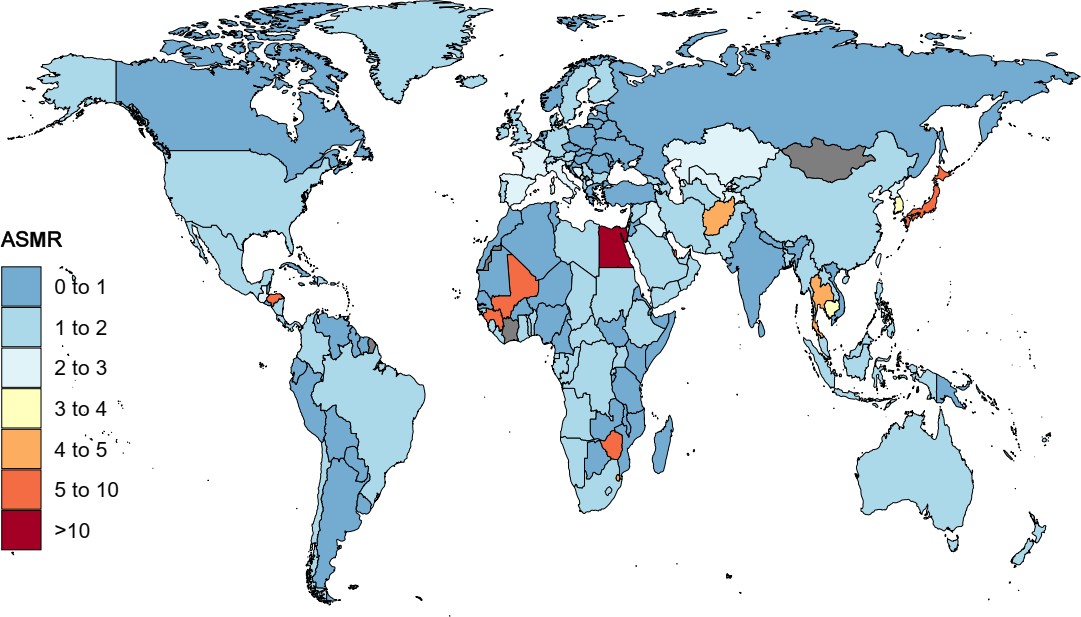


**Fig S9**: The global ASMR of liver cancer due to hepatitis C per 100,000 population in 2019, by country and territory. ASMR indicates Age-standardized motality rate.


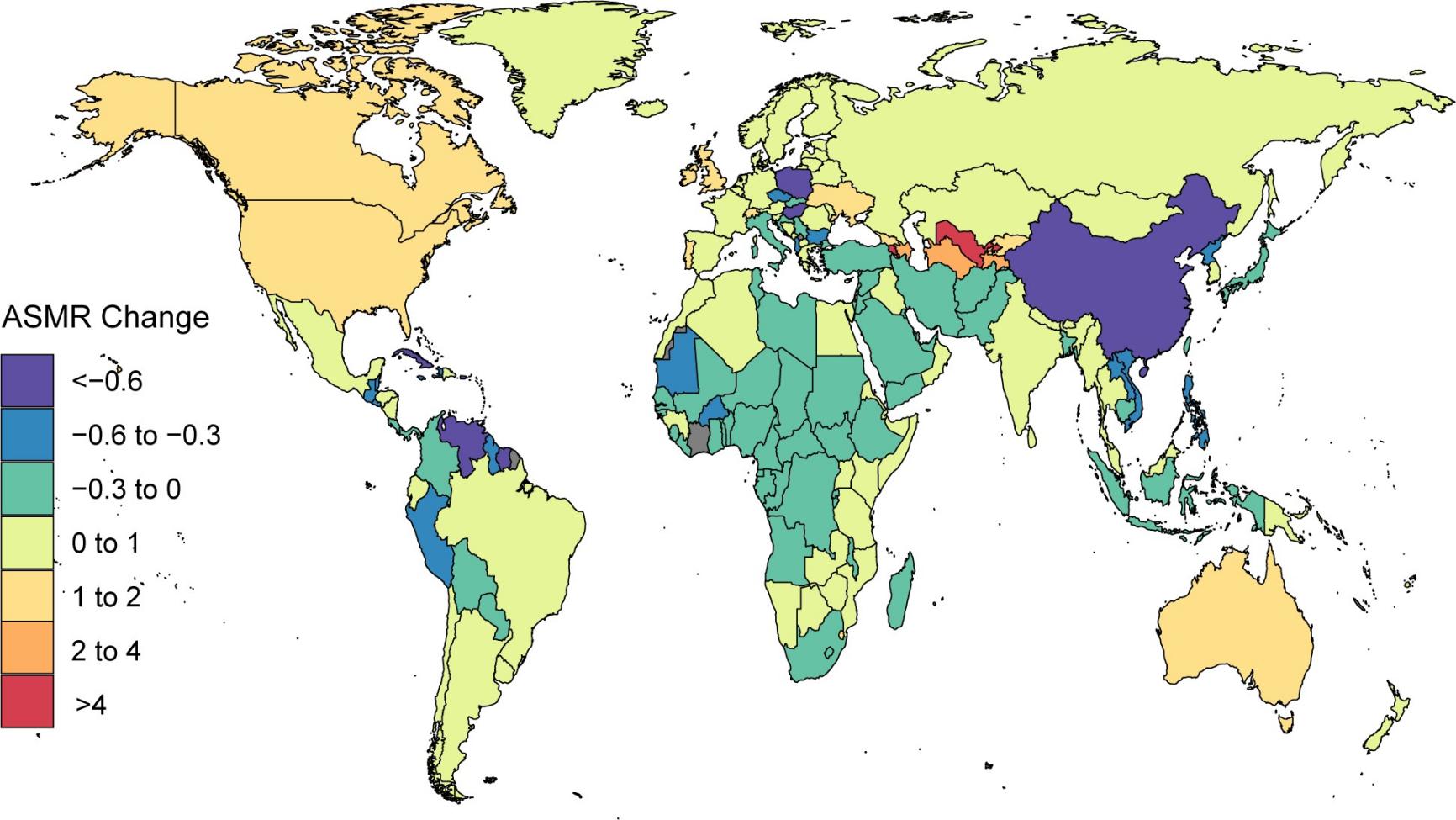


**Fig S10**: The global mortality ASR change of liver cancer due to hepatitis C per 100,000 population in 2019, by country and territory. ASMR indicates Age-standardized mortality rate.


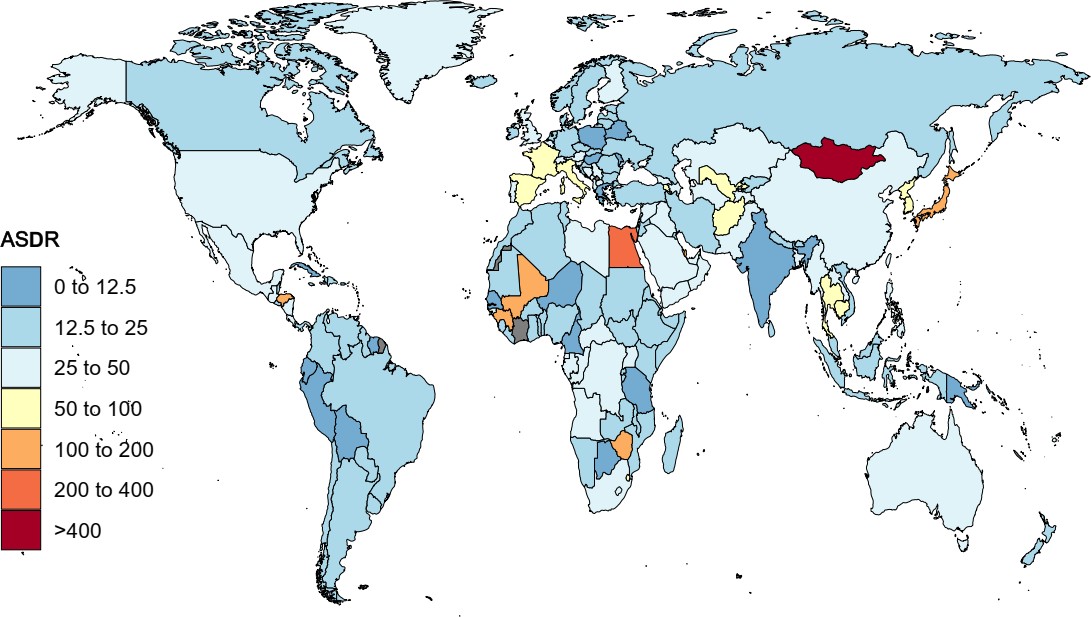


**Fig S11**: The global DALYs ASR of liver cancer due to hepatitis C per 100,000 population in 2019, by country and territory. ASDR indicates Age-standardized DALYs rate.


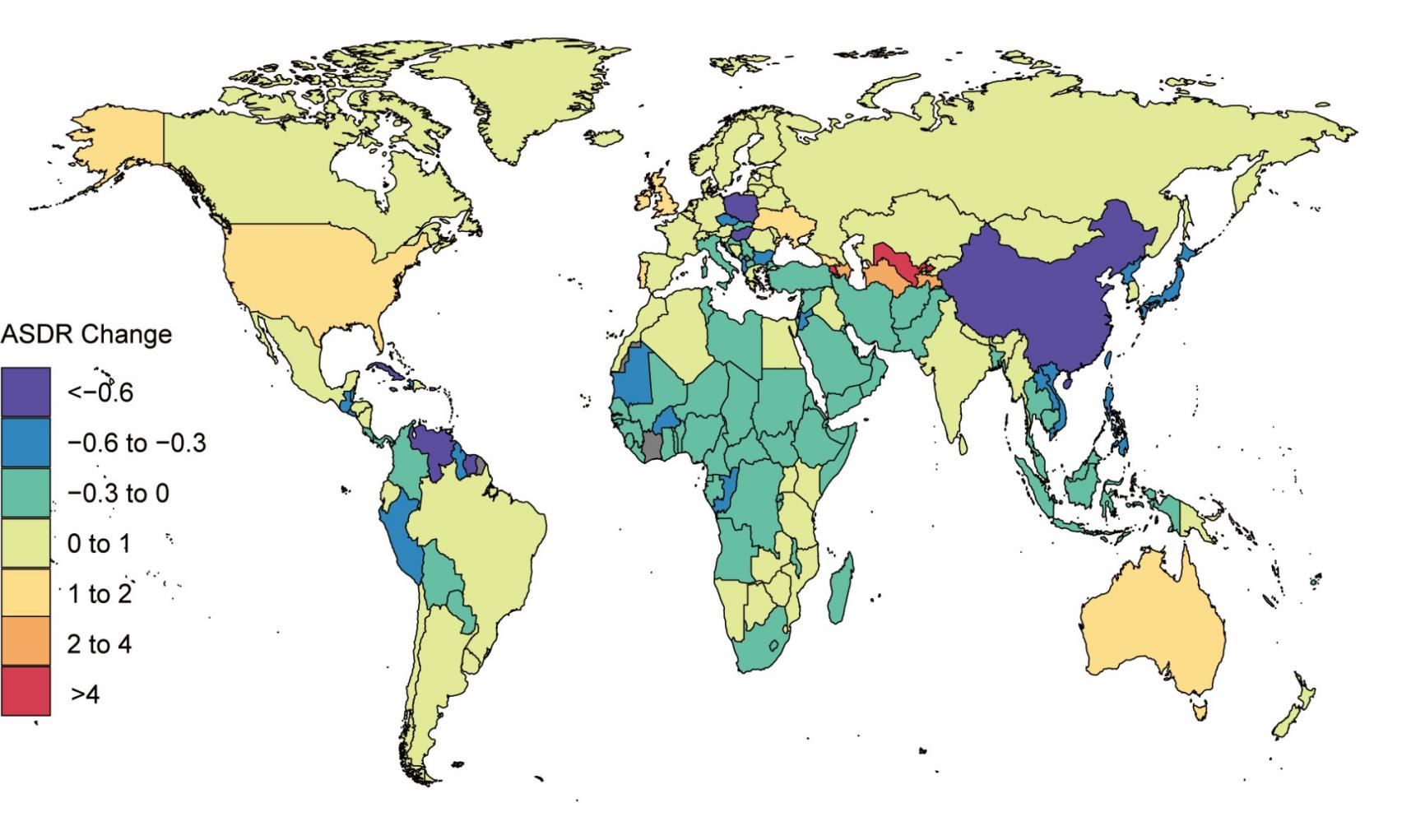


**Fig S12**: The global DALYs ASR chance of liver cancer due to hepatitis C per 100,000 population in 2019, by country and territory. ASDR indicates Age-standardized DALYs rate.


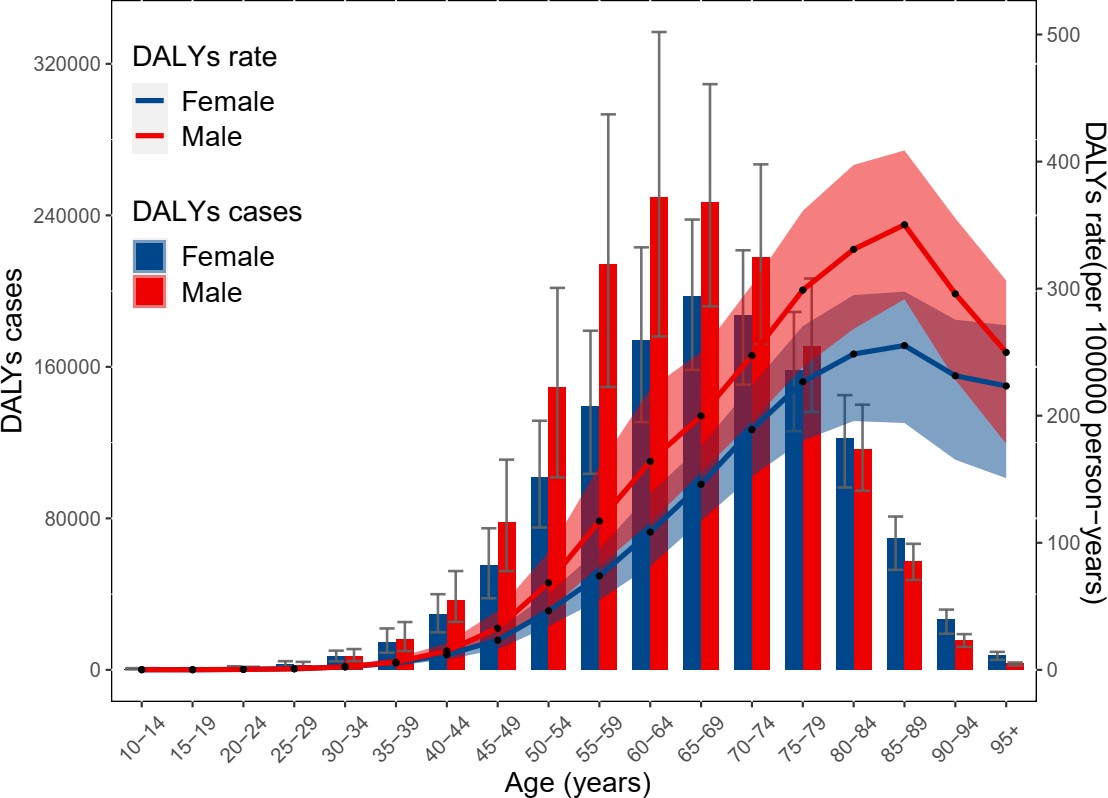


**Fig S13**: Global DALYs rate and DALYs cases of liver cancer due to hepatitis C per 100,000 population by age and sex, 2019.


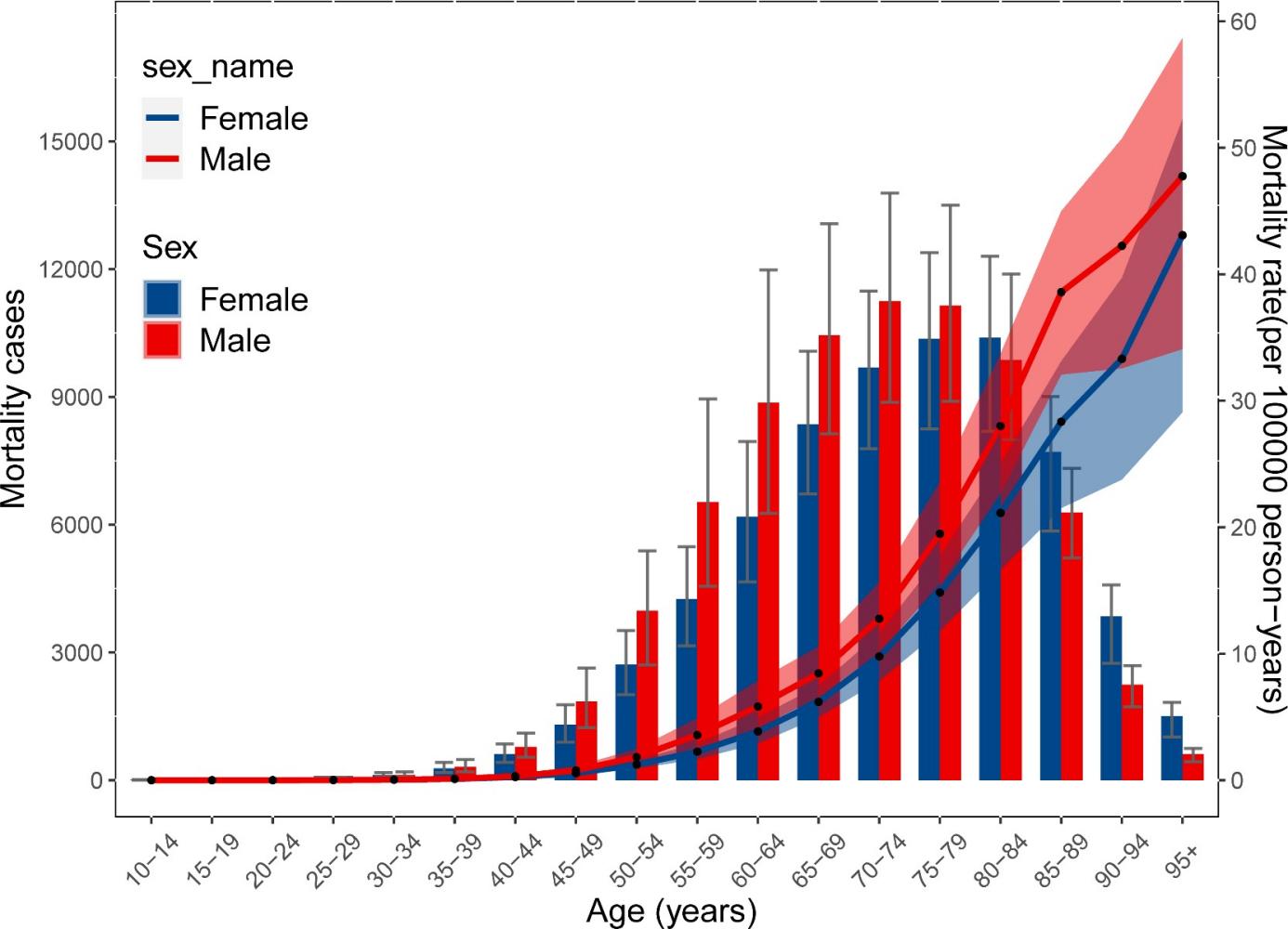


**Fig S14**: Global mortality rate and mortality cases of liver cancer due to hepatitis C per 100,000 population by age and sex, 2019.


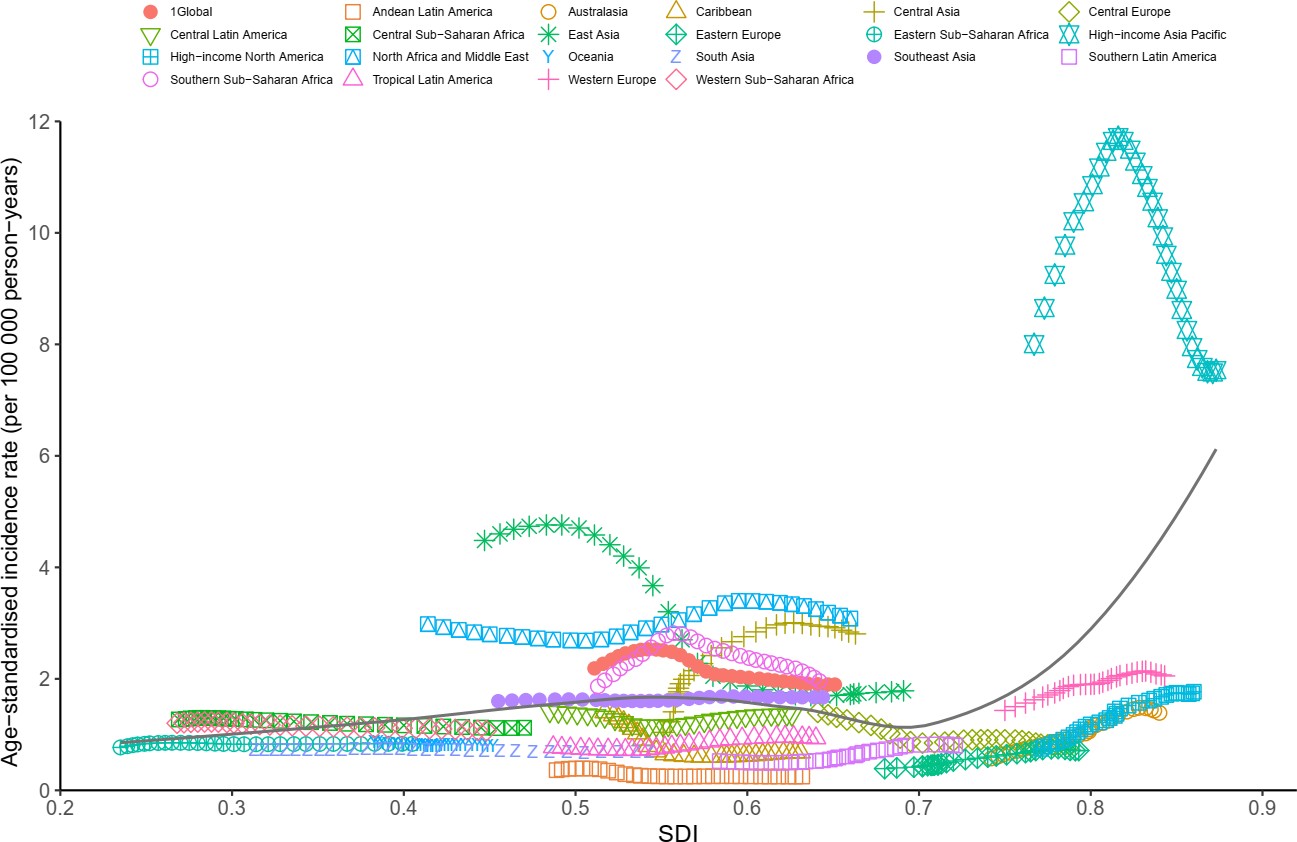


**Fig S15**: Age-standardized incidence rates of liver cancer due to hepatitis C in 21 GBD regions by SDI, 1990–2019.


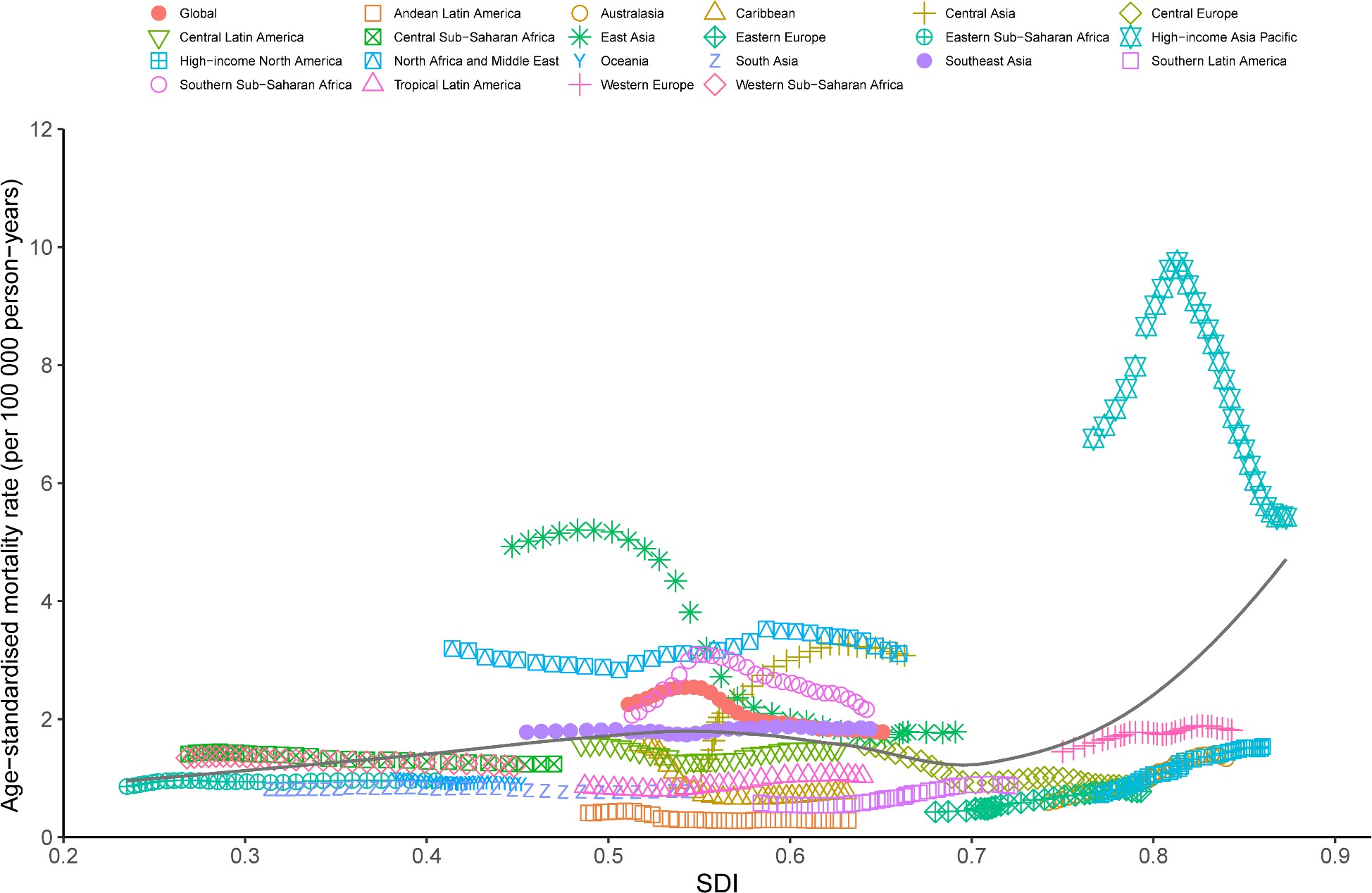


**Fig S16**: Age-standardized mortality rates of liver cancer due to hepatitis C in 21 GBD regions by SDI, 1990–2019.


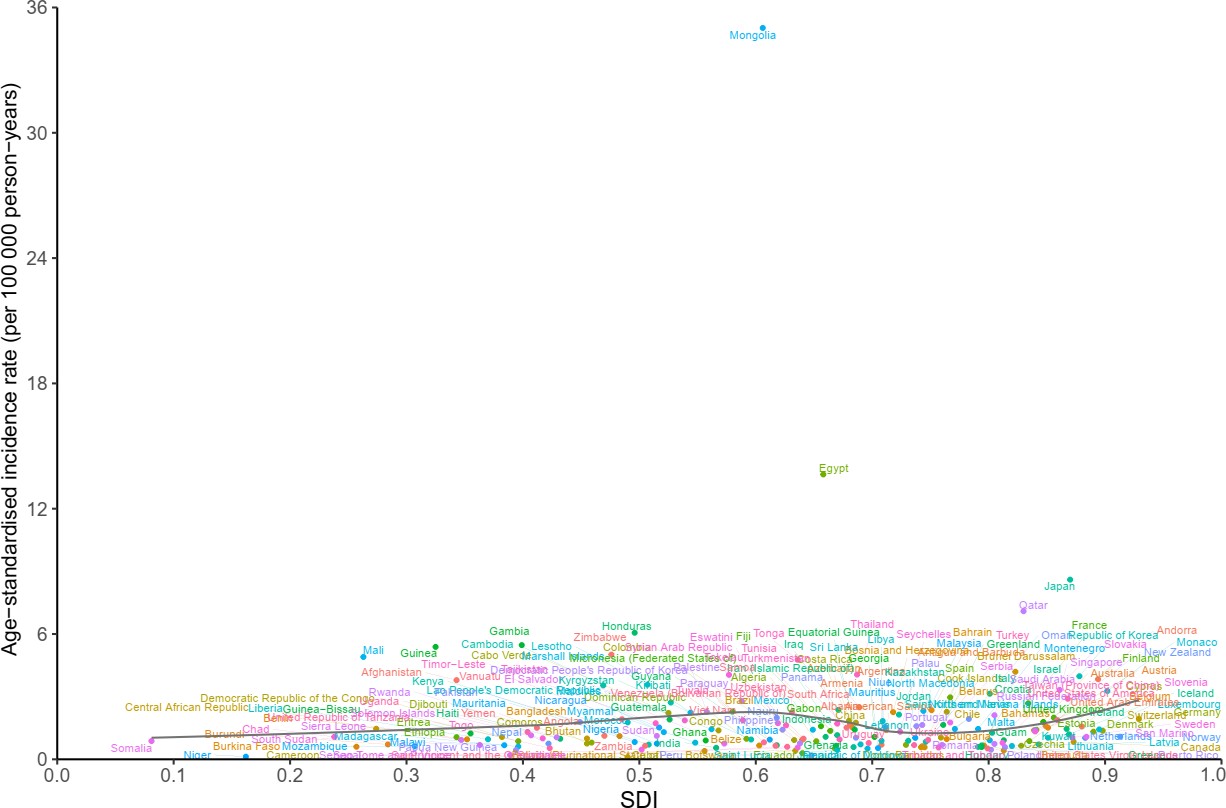


**Fig S17**: Age-standardized incidence rates for liver cancer due to hepatitis C by 204 countries and territories and SDI, 2019.


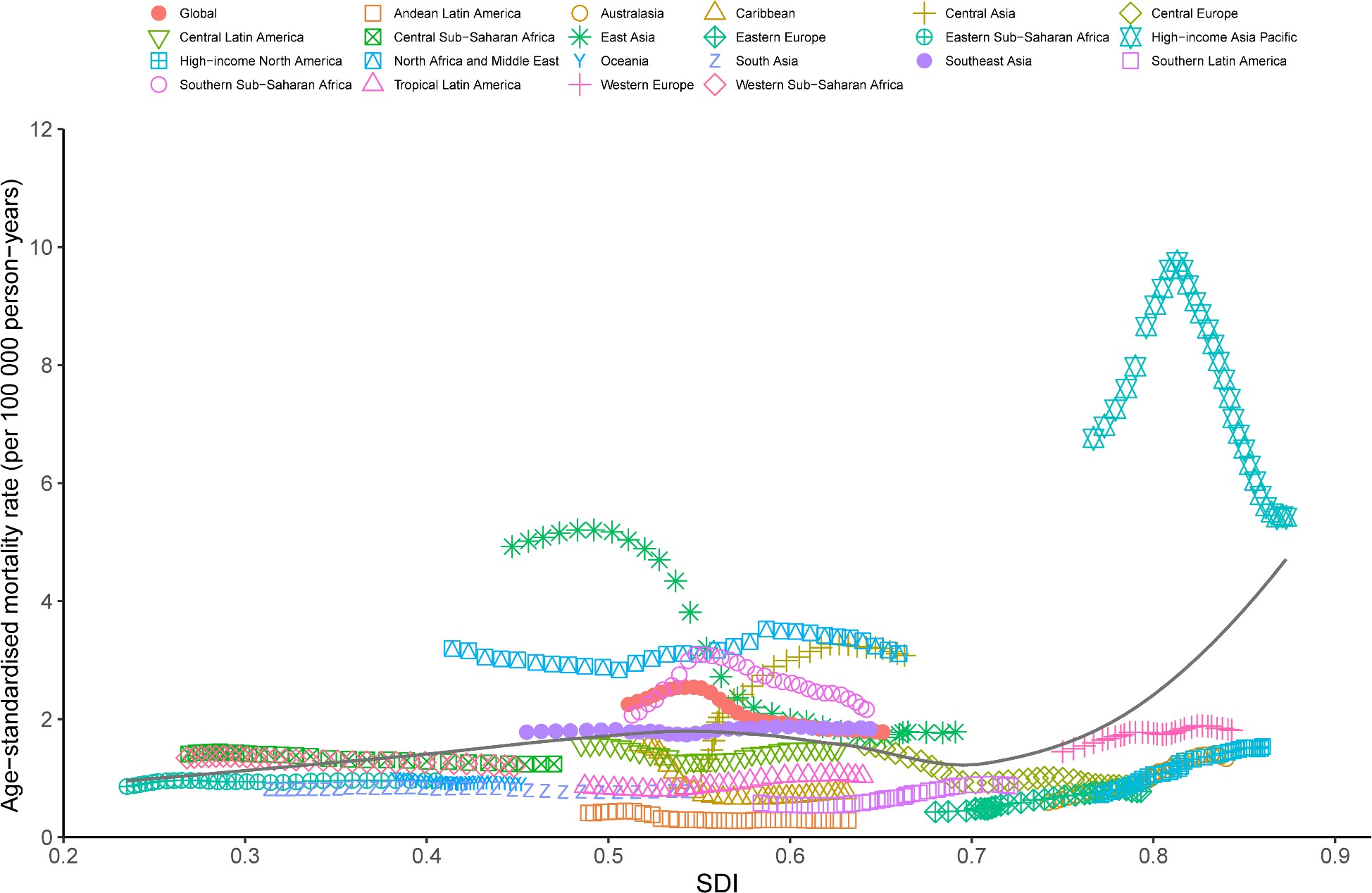


**Fig S18**: Age-standardized mortality rates for liver cancer due to hepatitis C by 204 countries and territories and SDI, 2019.
