# Supplementary material for: Burden of liver cancer due to hepatitis C from 1990 to 2019 at the global, regional, and national levels
Source: Front Oncol. 2023 Dec 19;13:1218901. doi: 10.3389/fonc.2023.1218901 (PMC10760495; doi:10.3389/fonc.2023.1218901)
Supplement: Supplementary file 3 [file Presentation_1.pptx]

## Slide 1
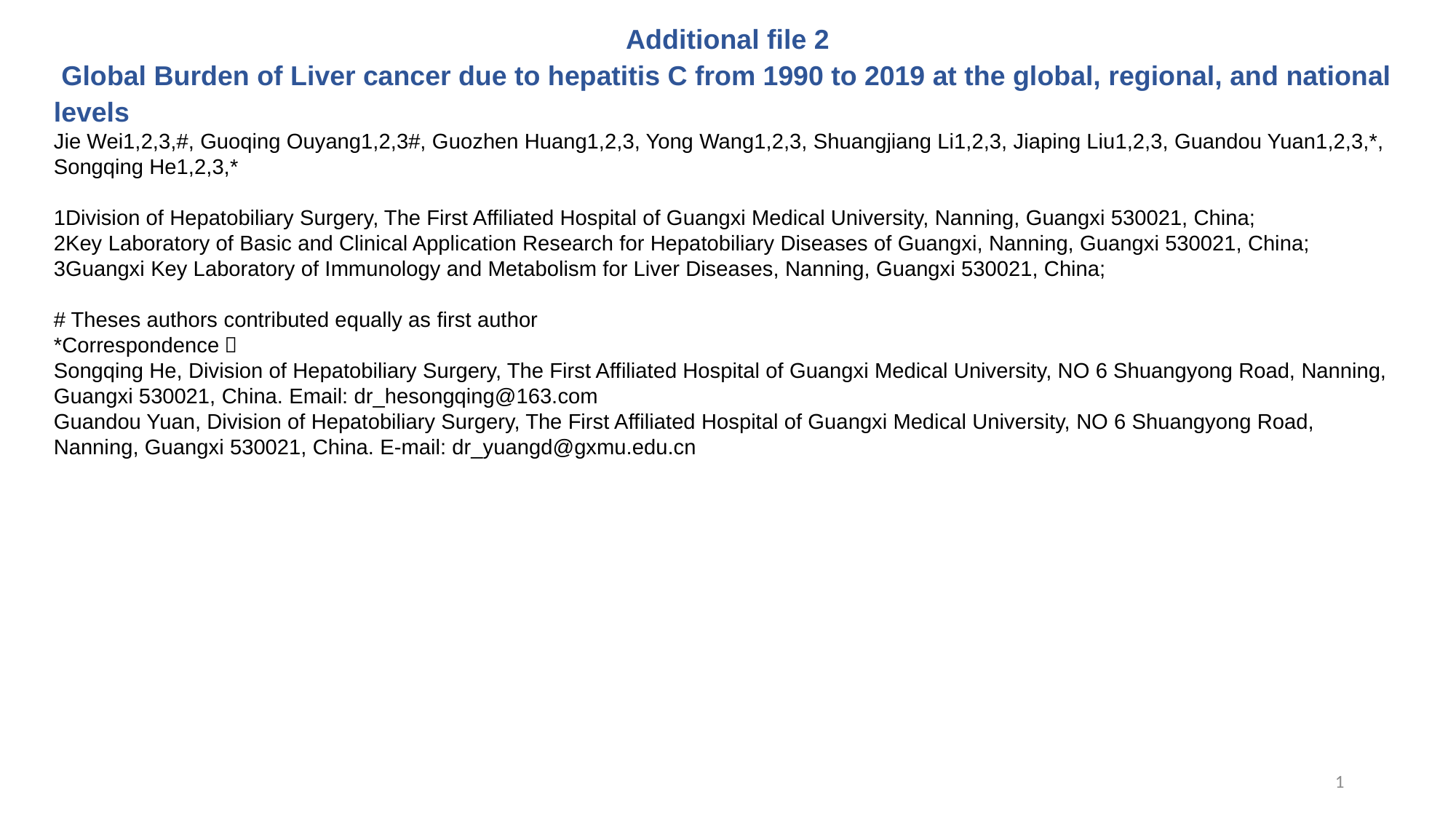

Additional file 2
 Global Burden of Liver cancer due to hepatitis C from 1990 to 2019 at the global, regional, and national levels
Jie Wei1,2,3,#, Guoqing Ouyang1,2,3#, Guozhen Huang1,2,3, Yong Wang1,2,3, Shuangjiang Li1,2,3, Jiaping Liu1,2,3, Guandou Yuan1,2,3,*, Songqing He1,2,3,*
1Division of Hepatobiliary Surgery, The First Affiliated Hospital of Guangxi Medical University, Nanning, Guangxi 530021, China;
2Key Laboratory of Basic and Clinical Application Research for Hepatobiliary Diseases of Guangxi, Nanning, Guangxi 530021, China;
3Guangxi Key Laboratory of Immunology and Metabolism for Liver Diseases, Nanning, Guangxi 530021, China;
# Theses authors contributed equally as first author
*Correspondence：
Songqing He, Division of Hepatobiliary Surgery, The First Affiliated Hospital of Guangxi Medical University, NO 6 Shuangyong Road, Nanning, Guangxi 530021, China. Email: dr_hesongqing@163.com
Guandou Yuan, Division of Hepatobiliary Surgery, The First Affiliated Hospital of Guangxi Medical University, NO 6 Shuangyong Road, Nanning, Guangxi 530021, China. E-mail: dr_yuangd@gxmu.edu.cn
1

## Slide 2
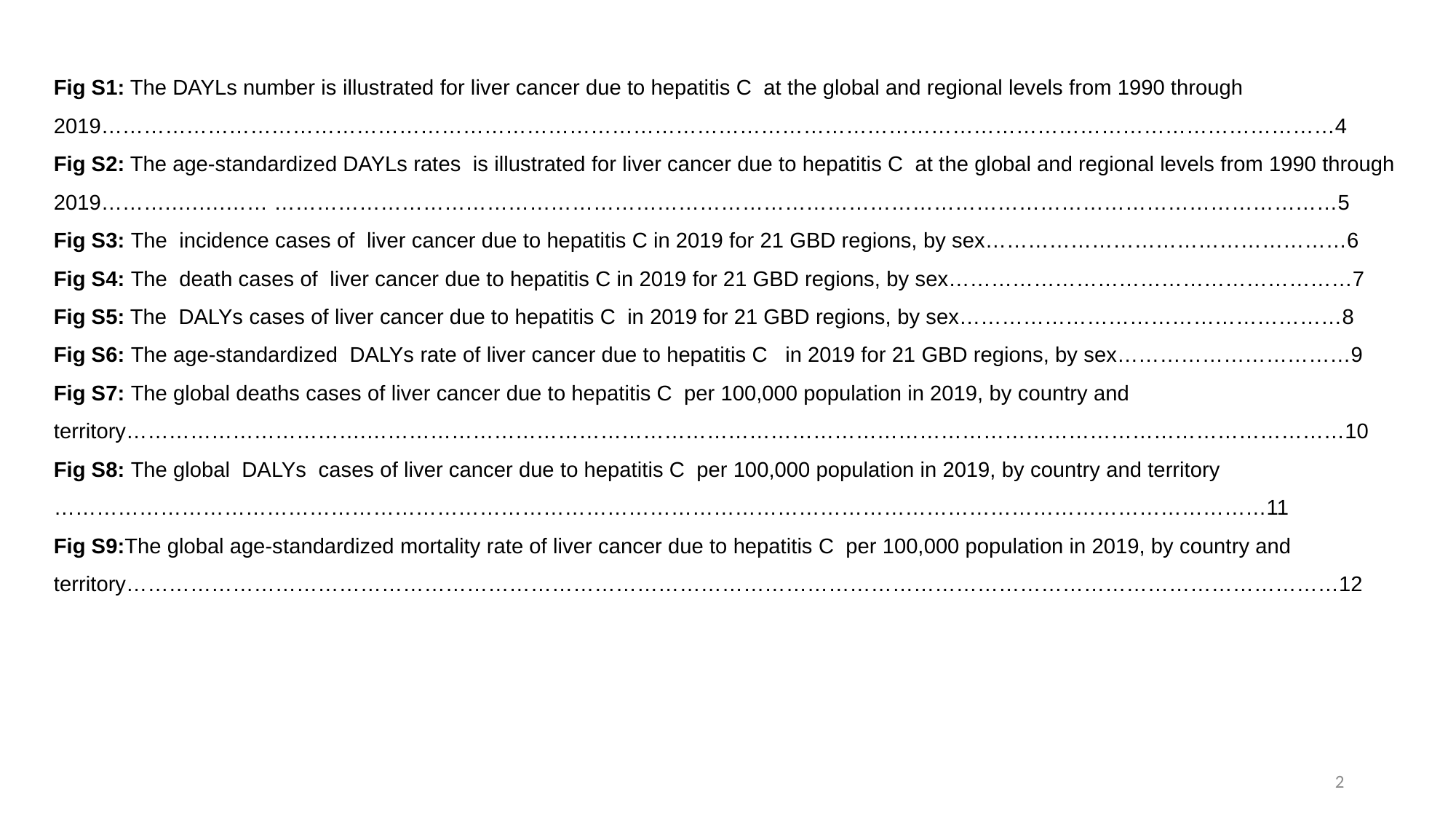

Fig S1: The DAYLs number is illustrated for liver cancer due to hepatitis C at the global and regional levels from 1990 through 2019…………………………………………………………………………………………………………………………………………………………4
Fig S2: The age-standardized DAYLs rates is illustrated for liver cancer due to hepatitis C at the global and regional levels from 1990 through 2019……….….….…… ……………………………………………………………………………………………………………………………………5
Fig S3: The incidence cases of liver cancer due to hepatitis C in 2019 for 21 GBD regions, by sex……………………………………………6
Fig S4: The death cases of liver cancer due to hepatitis C in 2019 for 21 GBD regions, by sex…………………………………………………7
Fig S5: The DALYs cases of liver cancer due to hepatitis C in 2019 for 21 GBD regions, by sex………………………………………………8
Fig S6: The age-standardized DALYs rate of liver cancer due to hepatitis C in 2019 for 21 GBD regions, by sex……………………………9
Fig S7: The global deaths cases of liver cancer due to hepatitis C per 100,000 population in 2019, by country and territory…………………………….…………………………………………………………………………………………………………………………10
Fig S8: The global DALYs cases of liver cancer due to hepatitis C per 100,000 population in 2019, by country and territory ………………………………………………………………………………………………………………………………………………………11
Fig S9:The global age-standardized mortality rate of liver cancer due to hepatitis C per 100,000 population in 2019, by country and territory………………………………………………………………………………………………………………………………………………………12
2

## Slide 3
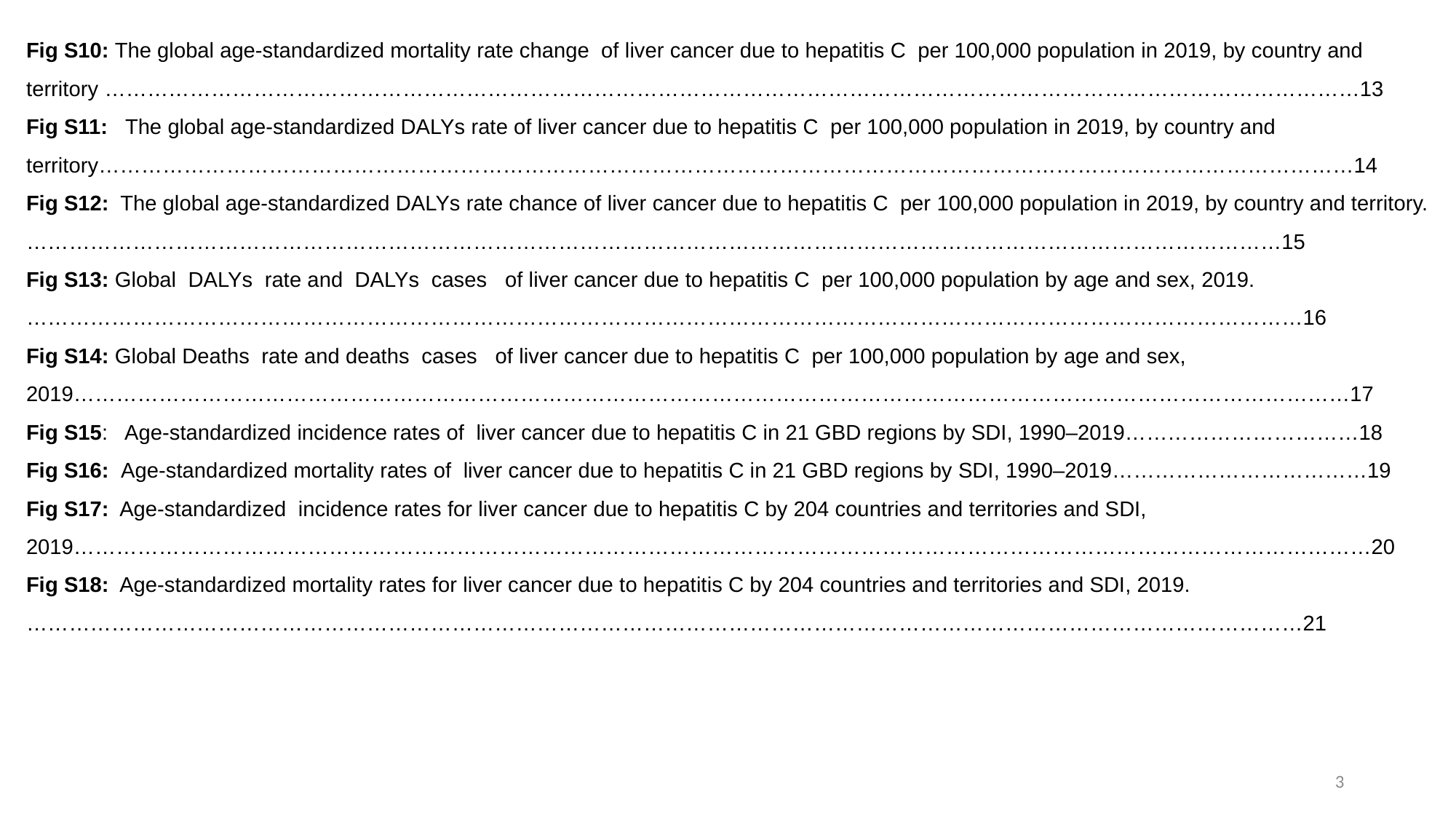

Fig S10: The global age-standardized mortality rate change of liver cancer due to hepatitis C per 100,000 population in 2019, by country and territory ……………………………………………………………………………………………………………………………………………………………13
Fig S11: The global age-standardized DALYs rate of liver cancer due to hepatitis C per 100,000 population in 2019, by country and territory……………………………………………………………………………………………………………………………………………………………14
Fig S12: The global age-standardized DALYs rate chance of liver cancer due to hepatitis C per 100,000 population in 2019, by country and territory. ……………………………………………………………………………………………………………………………………………………………15
Fig S13: Global DALYs rate and DALYs cases of liver cancer due to hepatitis C per 100,000 population by age and sex, 2019.………………………………………………………………………………………………………………………………………………………………16
Fig S14: Global Deaths rate and deaths cases of liver cancer due to hepatitis C per 100,000 population by age and sex, 2019………………………………………………………………………………………………………………………………………………………………17
Fig S15: Age-standardized incidence rates of liver cancer due to hepatitis C in 21 GBD regions by SDI, 1990–2019……………………………18
Fig S16: Age-standardized mortality rates of liver cancer due to hepatitis C in 21 GBD regions by SDI, 1990–2019………………………………19
Fig S17: Age-standardized incidence rates for liver cancer due to hepatitis C by 204 countries and territories and SDI, 2019…………………………………………………………………………………………………………………………………………………………………20
Fig S18: Age-standardized mortality rates for liver cancer due to hepatitis C by 204 countries and territories and SDI, 2019. ………………………………………………………………………………………………………………………………………………………………21
3

## Slide 4
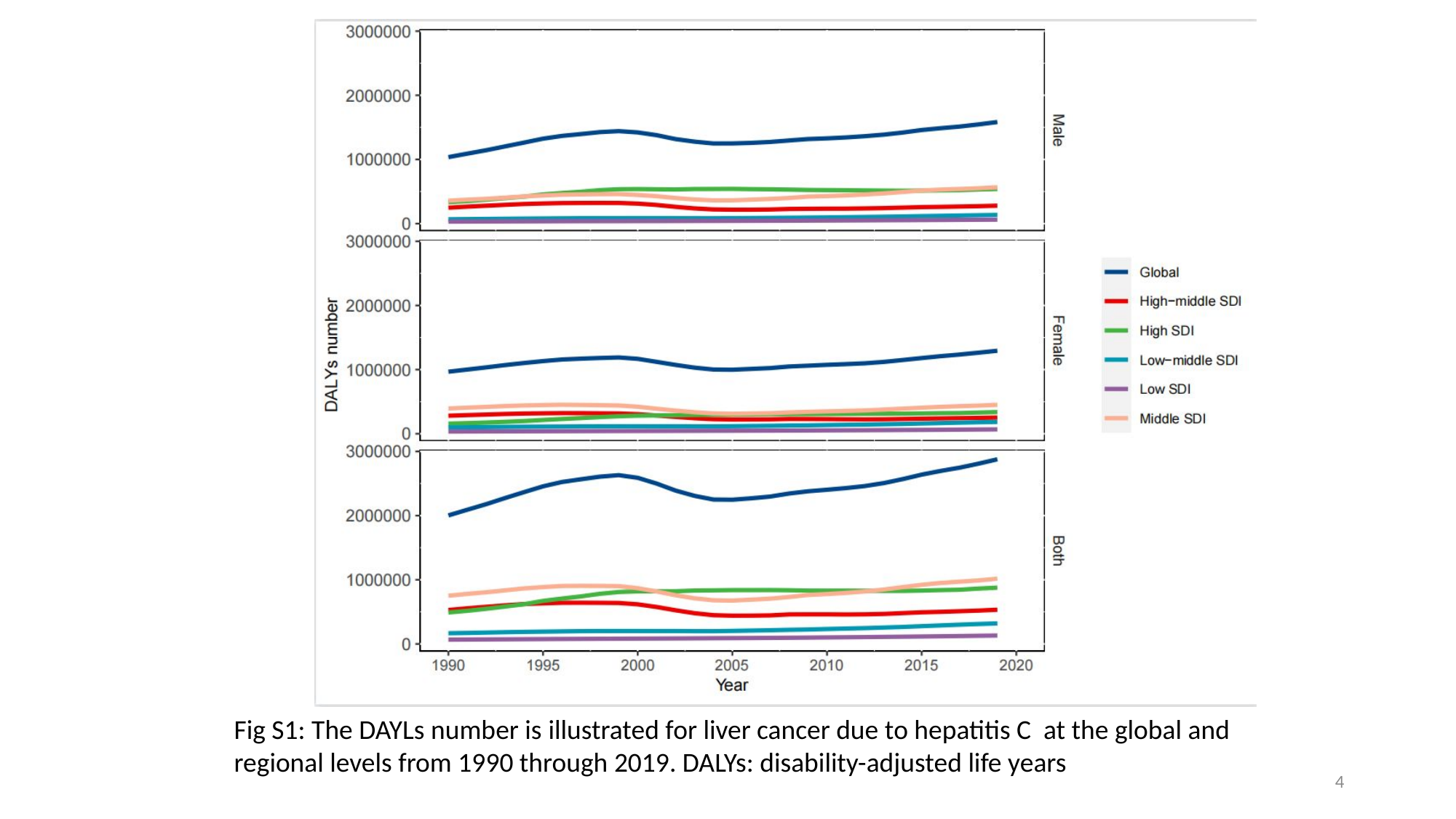

Fig S1: The DAYLs number is illustrated for liver cancer due to hepatitis C at the global and regional levels from 1990 through 2019. DALYs: disability-adjusted life years
4

## Slide 5
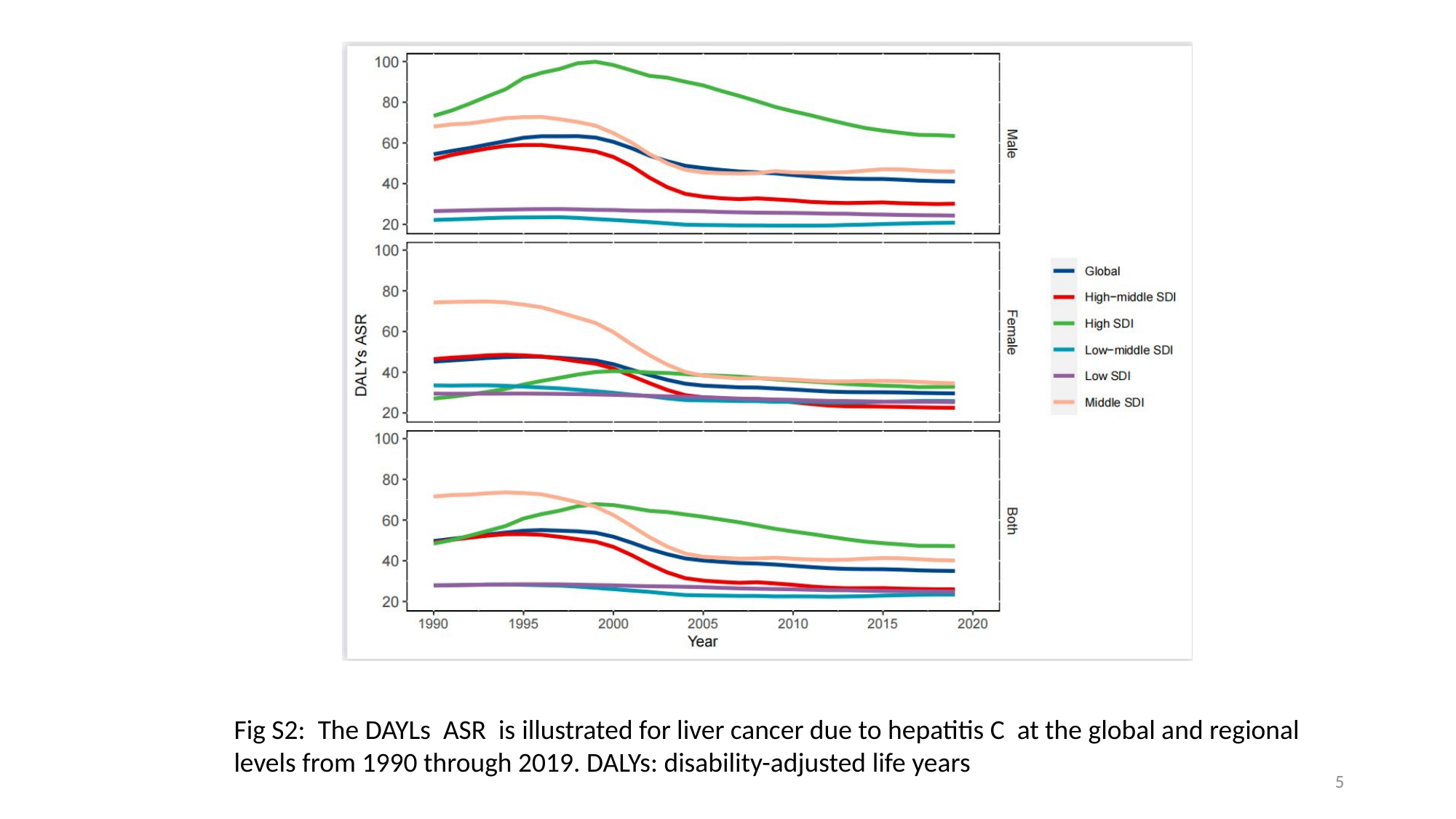

Fig S2: The DAYLs ASR is illustrated for liver cancer due to hepatitis C at the global and regional levels from 1990 through 2019. DALYs: disability-adjusted life years
5

## Slide 6
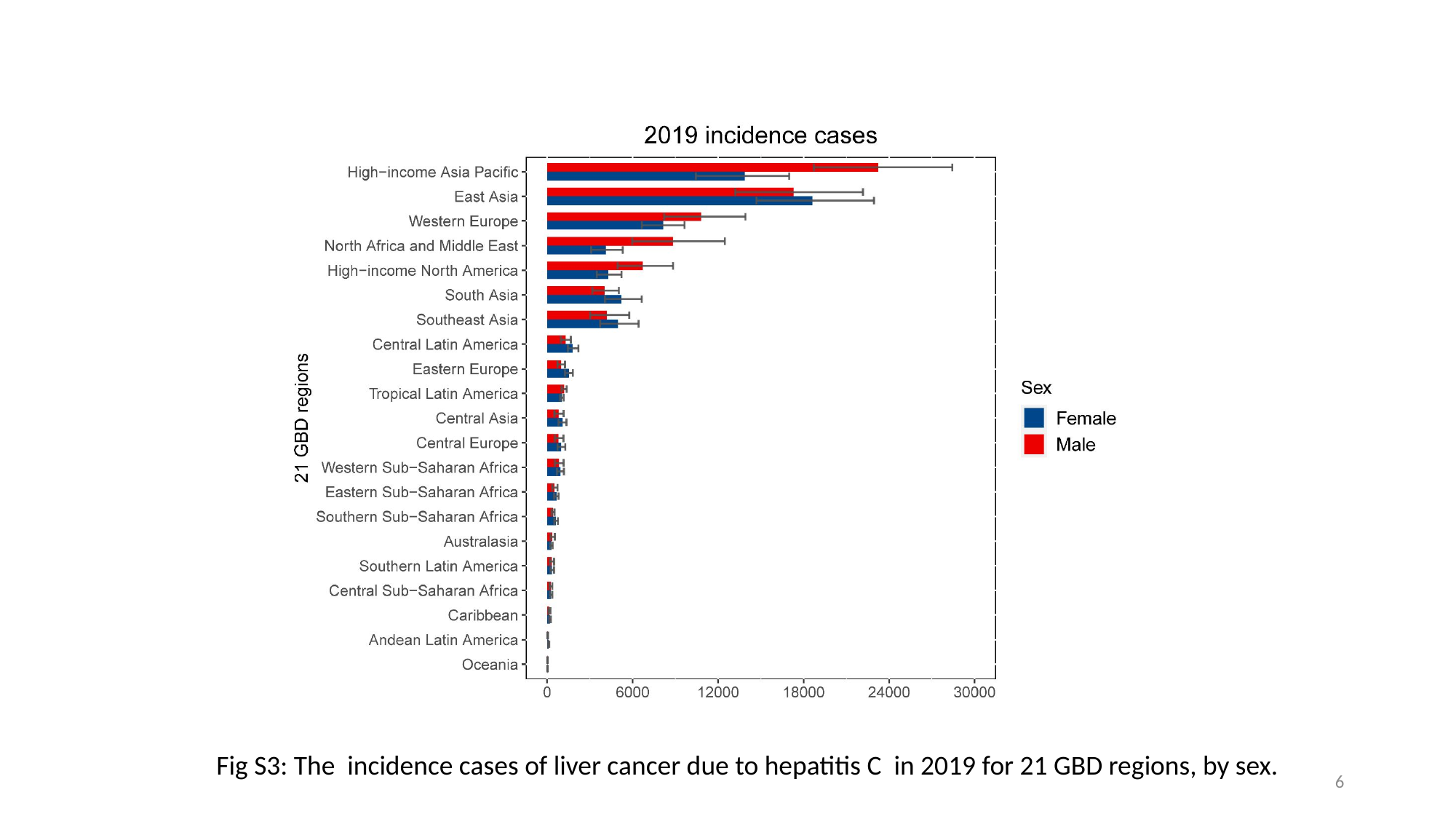

Fig S3: The incidence cases of liver cancer due to hepatitis C in 2019 for 21 GBD regions, by sex.
6

## Slide 7
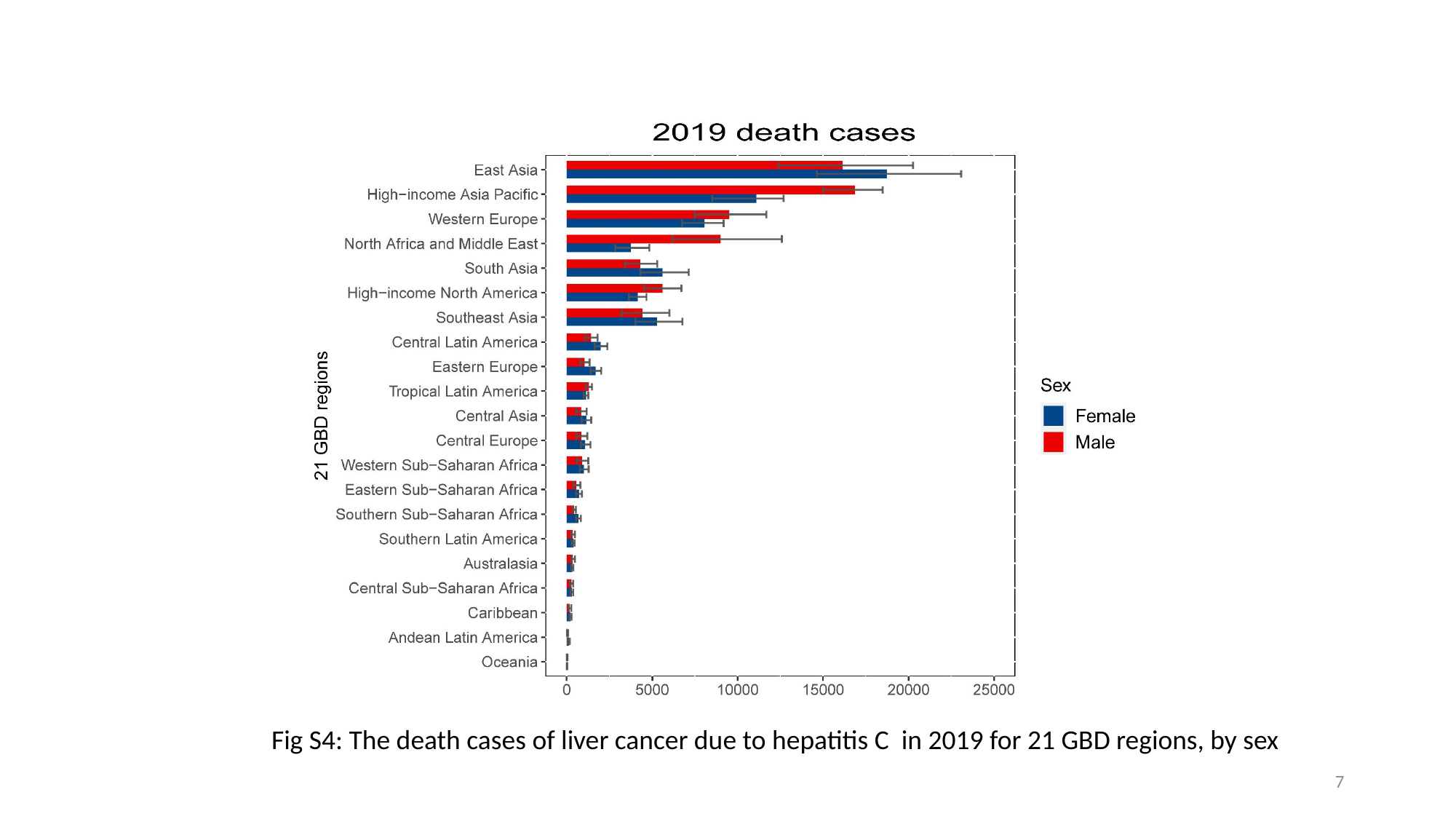

Fig S4: The death cases of liver cancer due to hepatitis C in 2019 for 21 GBD regions, by sex
7

## Slide 8
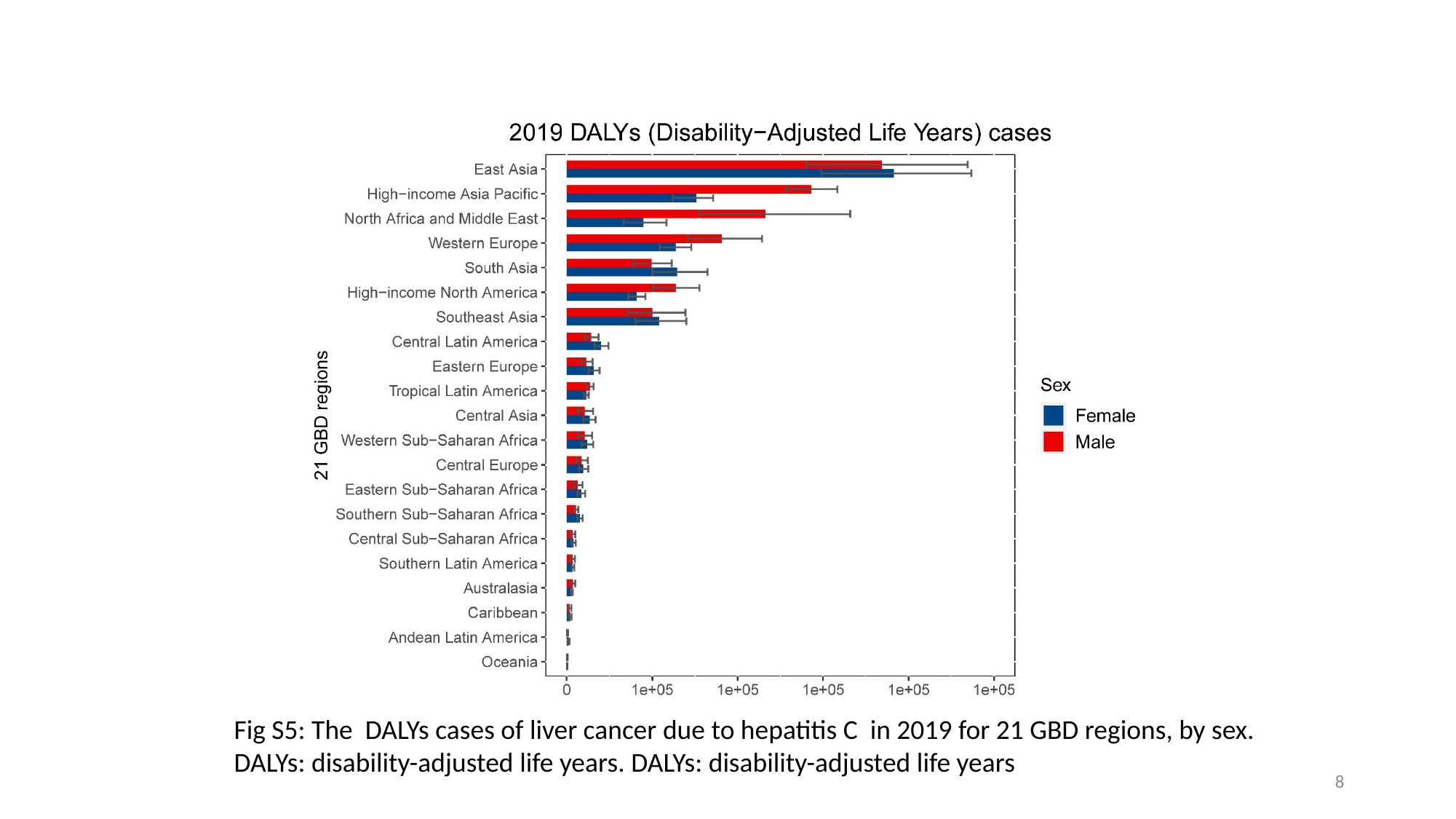

Fig S5: The DALYs cases of liver cancer due to hepatitis C in 2019 for 21 GBD regions, by sex. DALYs: disability-adjusted life years. DALYs: disability-adjusted life years
8

## Slide 9
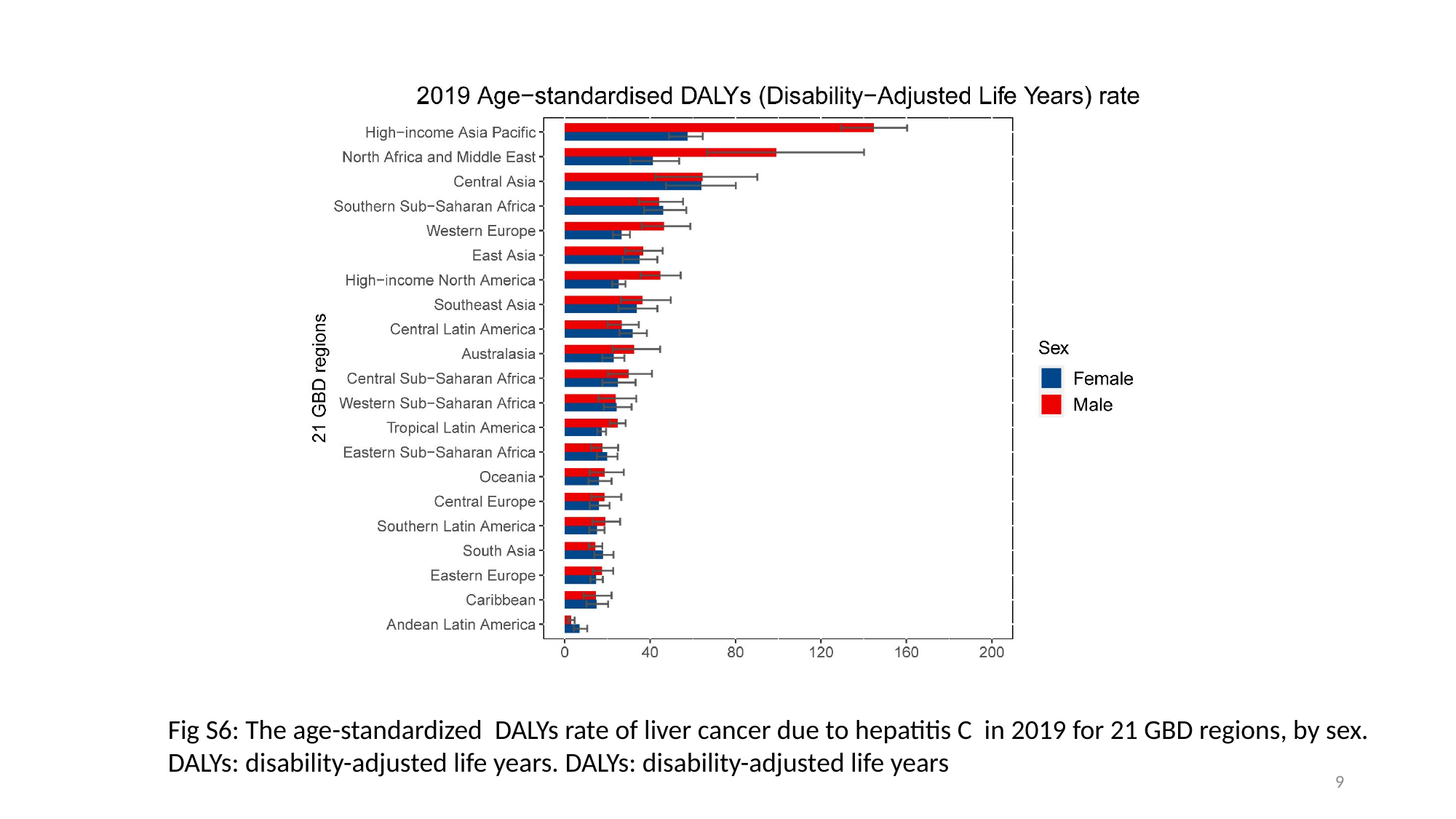

Fig S6: The age-standardized DALYs rate of liver cancer due to hepatitis C in 2019 for 21 GBD regions, by sex. DALYs: disability-adjusted life years. DALYs: disability-adjusted life years
9

## Slide 10
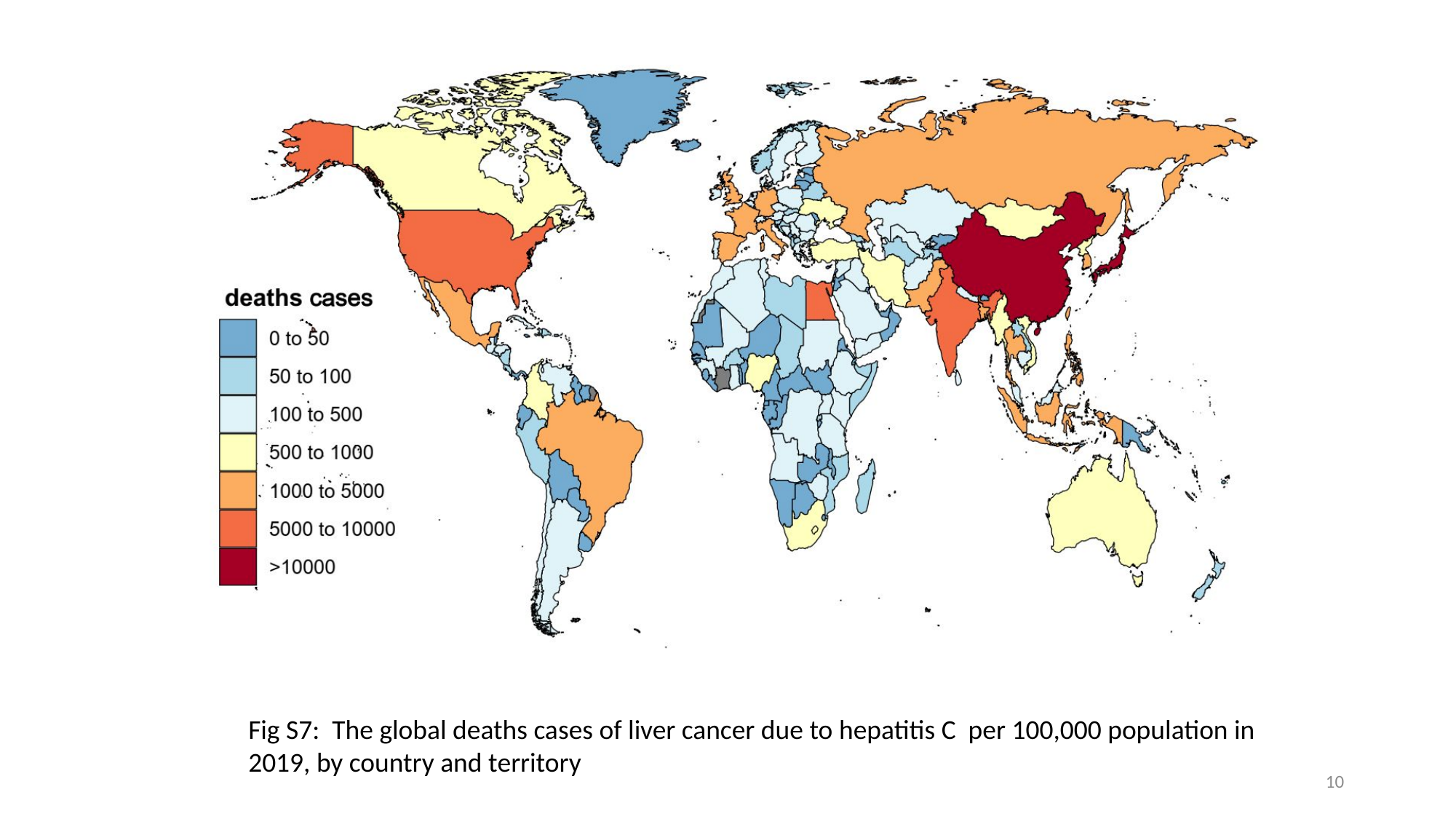

Fig S7: The global deaths cases of liver cancer due to hepatitis C per 100,000 population in 2019, by country and territory
10

## Slide 11
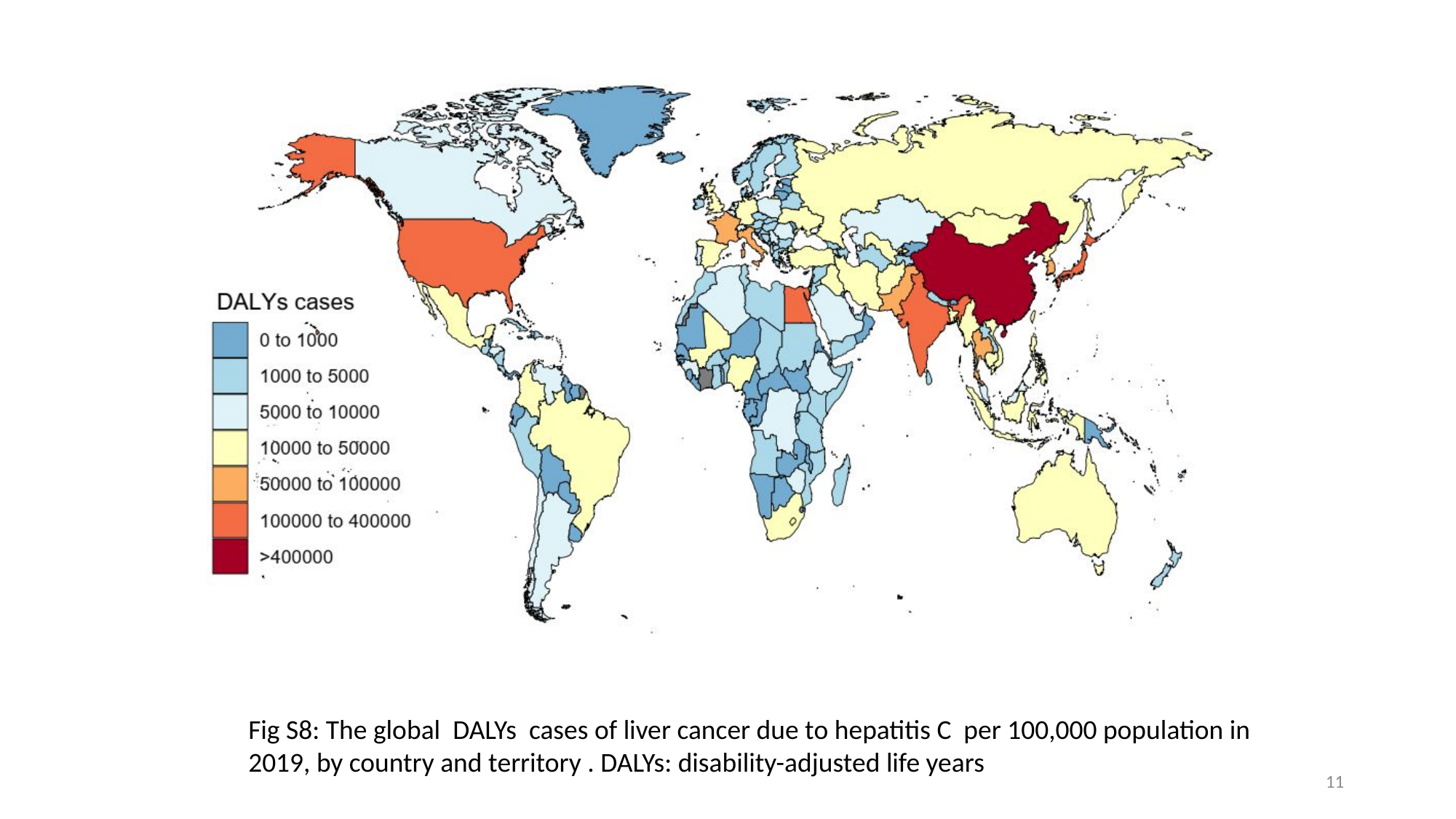

Fig S8: The global DALYs cases of liver cancer due to hepatitis C per 100,000 population in 2019, by country and territory . DALYs: disability-adjusted life years
11

## Slide 12
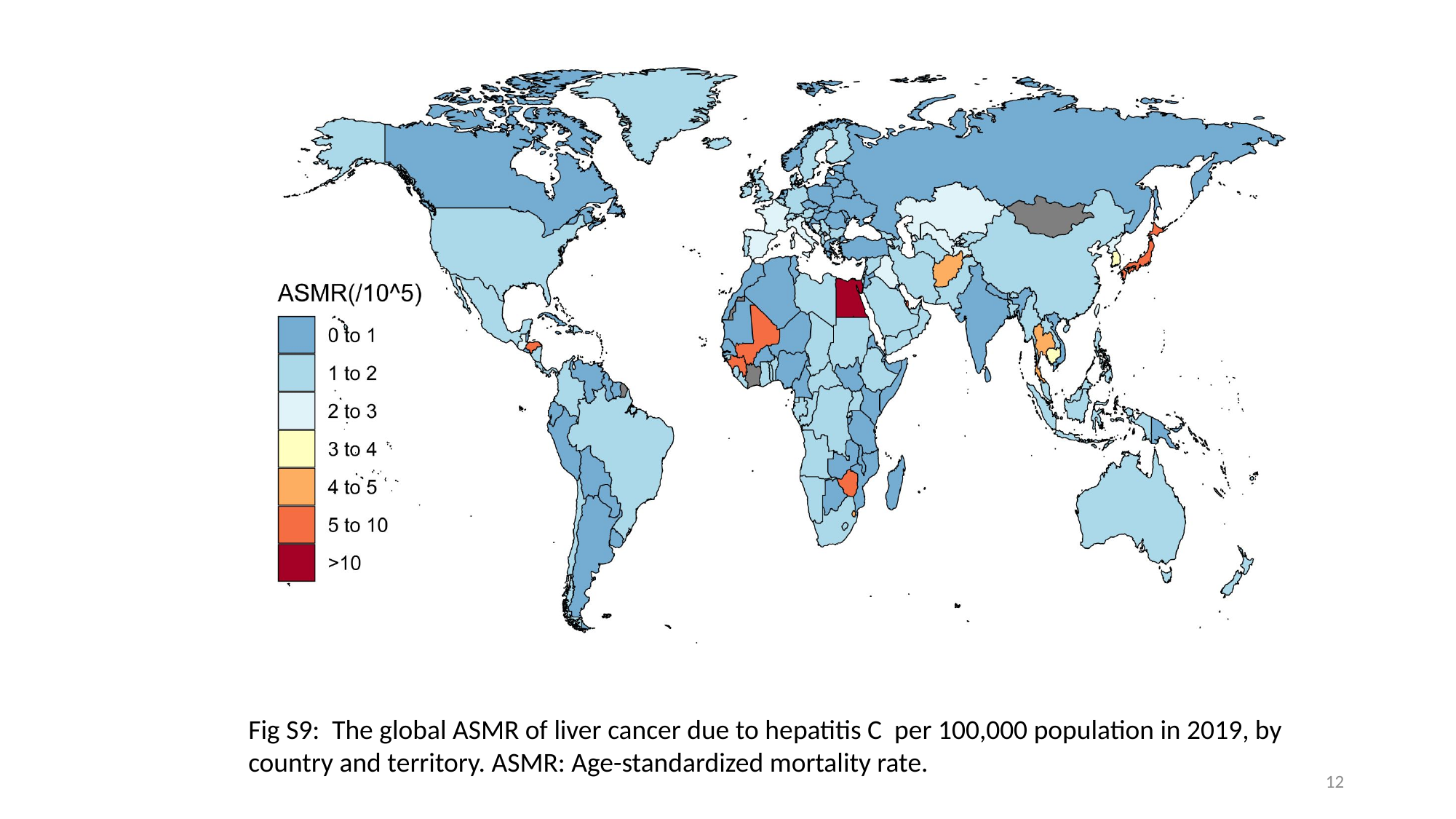

Fig S9: The global ASMR of liver cancer due to hepatitis C per 100,000 population in 2019, by country and territory. ASMR: Age-standardized mortality rate.
12

## Slide 13
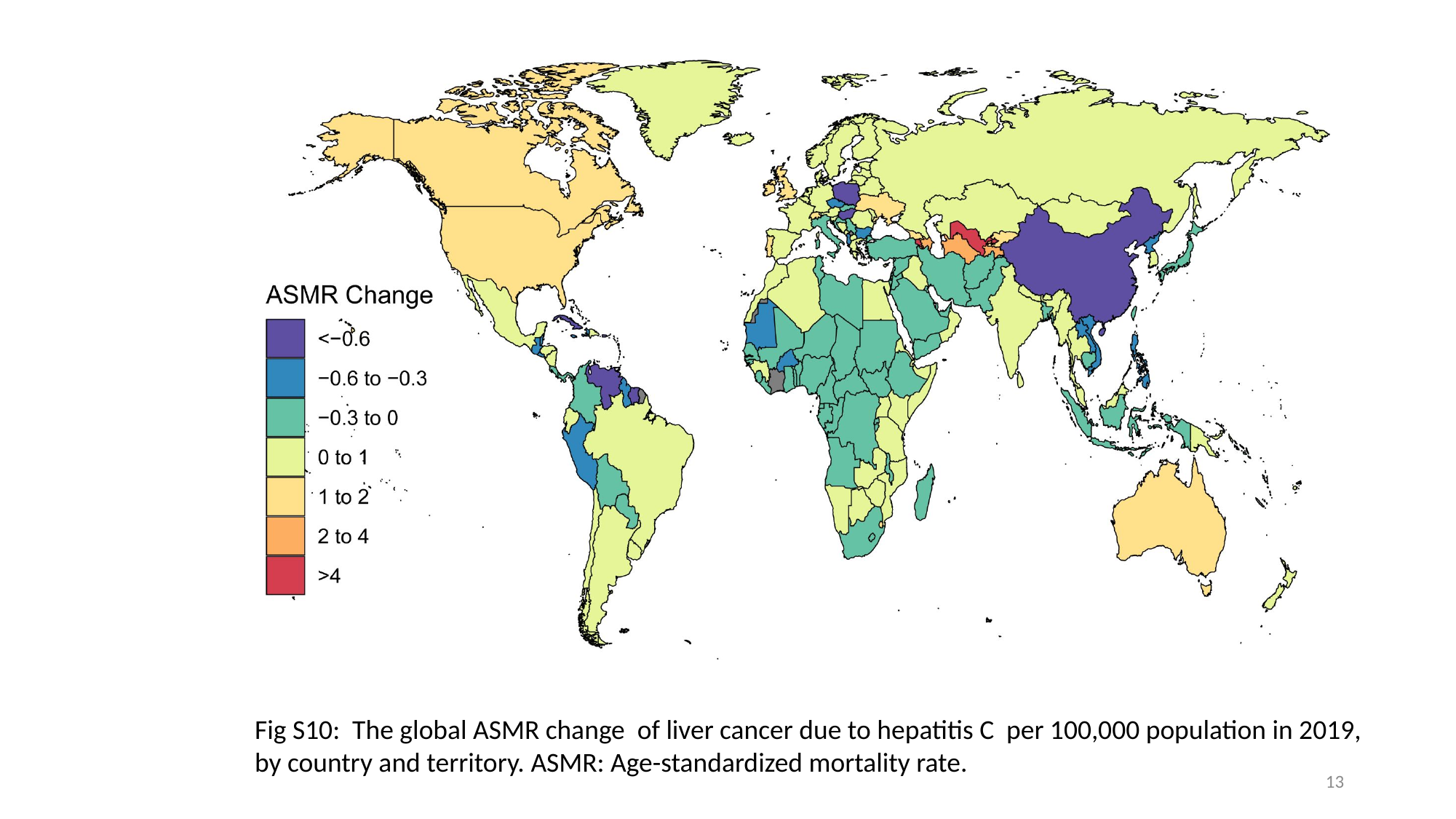

Fig S10: The global ASMR change of liver cancer due to hepatitis C per 100,000 population in 2019, by country and territory. ASMR: Age-standardized mortality rate.
13

## Slide 14
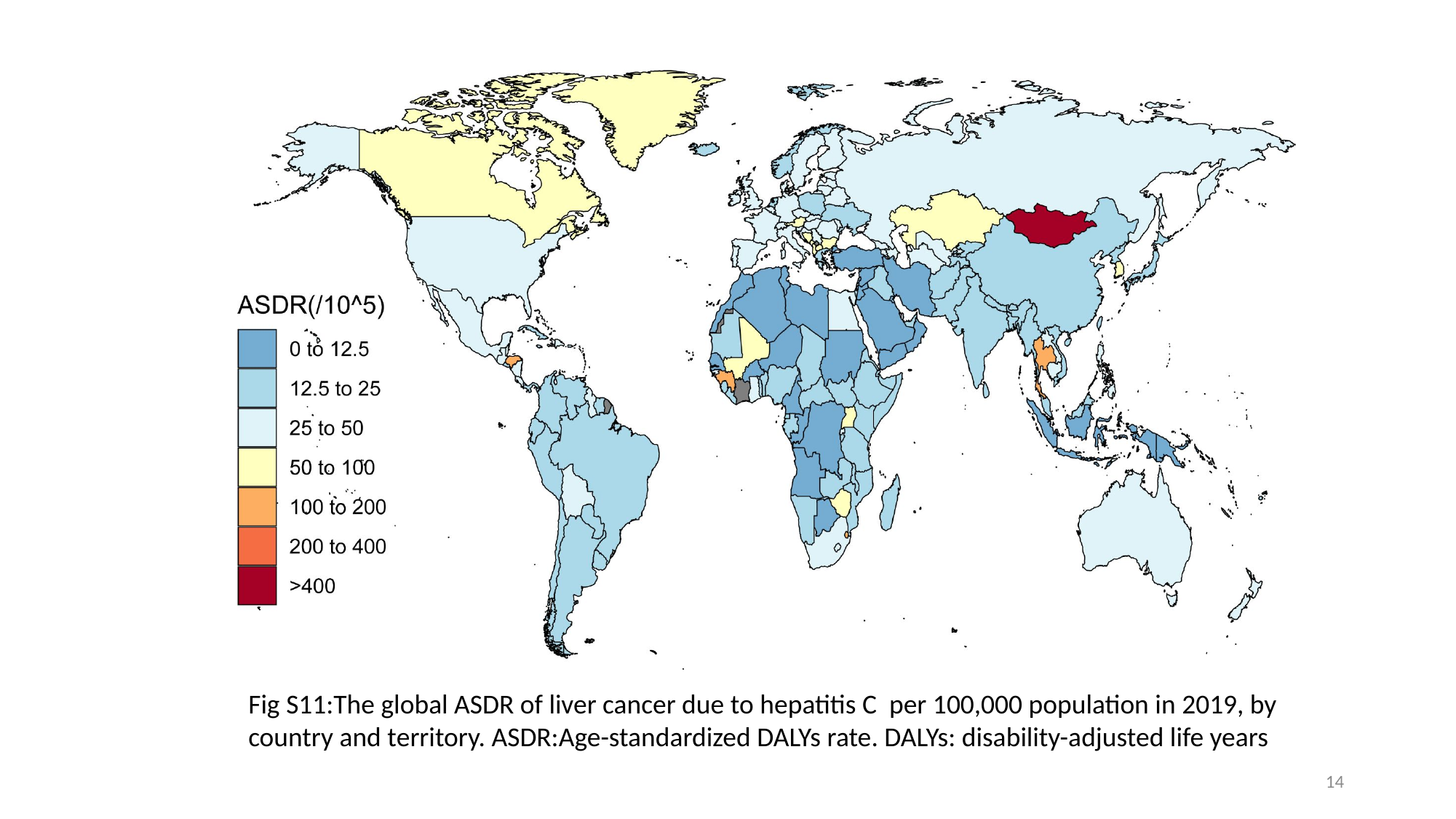

Fig S11:The global ASDR of liver cancer due to hepatitis C per 100,000 population in 2019, by country and territory. ASDR:Age-standardized DALYs rate. DALYs: disability-adjusted life years
14

## Slide 15
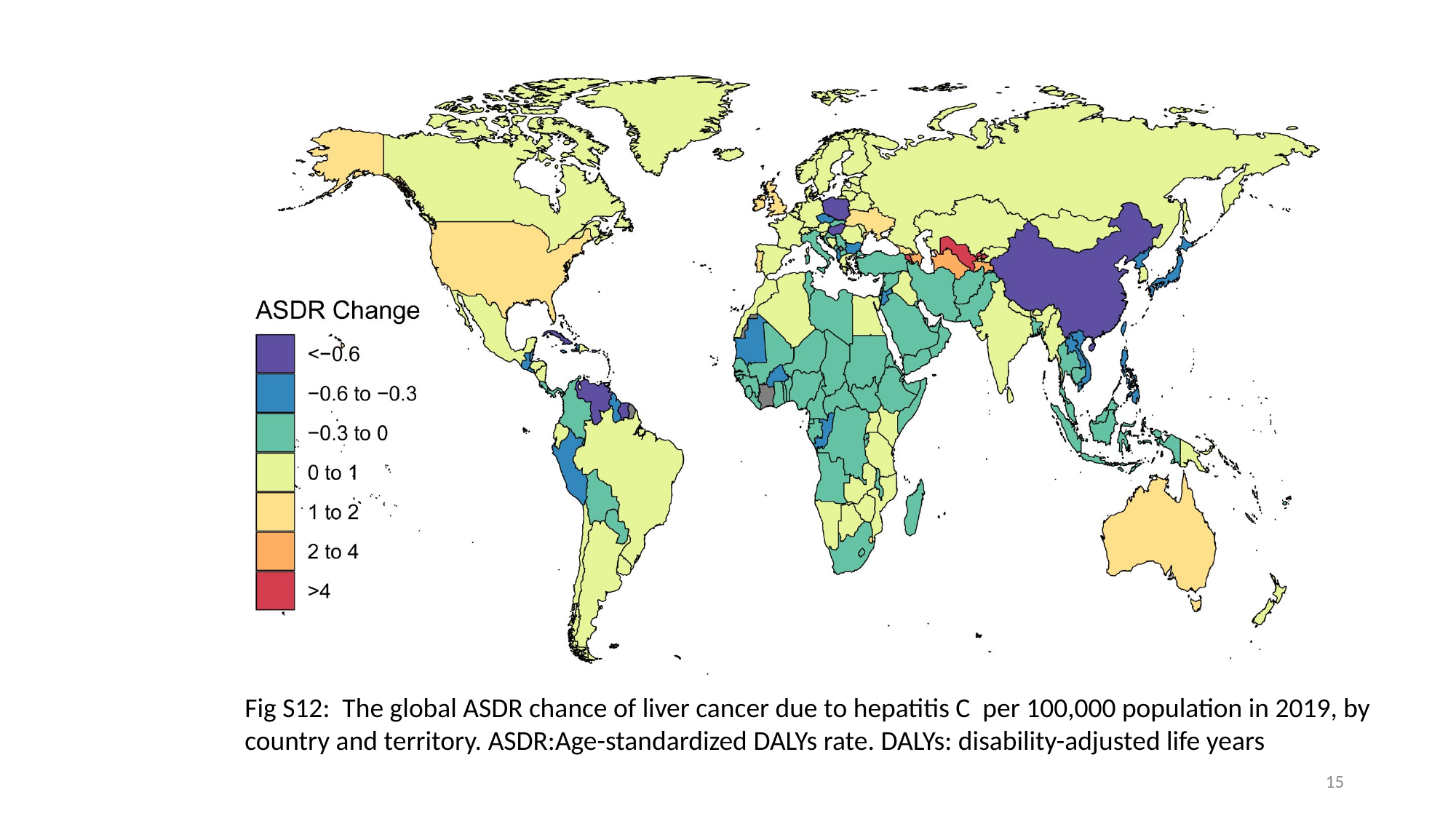

Fig S12: The global ASDR chance of liver cancer due to hepatitis C per 100,000 population in 2019, by country and territory. ASDR:Age-standardized DALYs rate. DALYs: disability-adjusted life years
15

## Slide 16
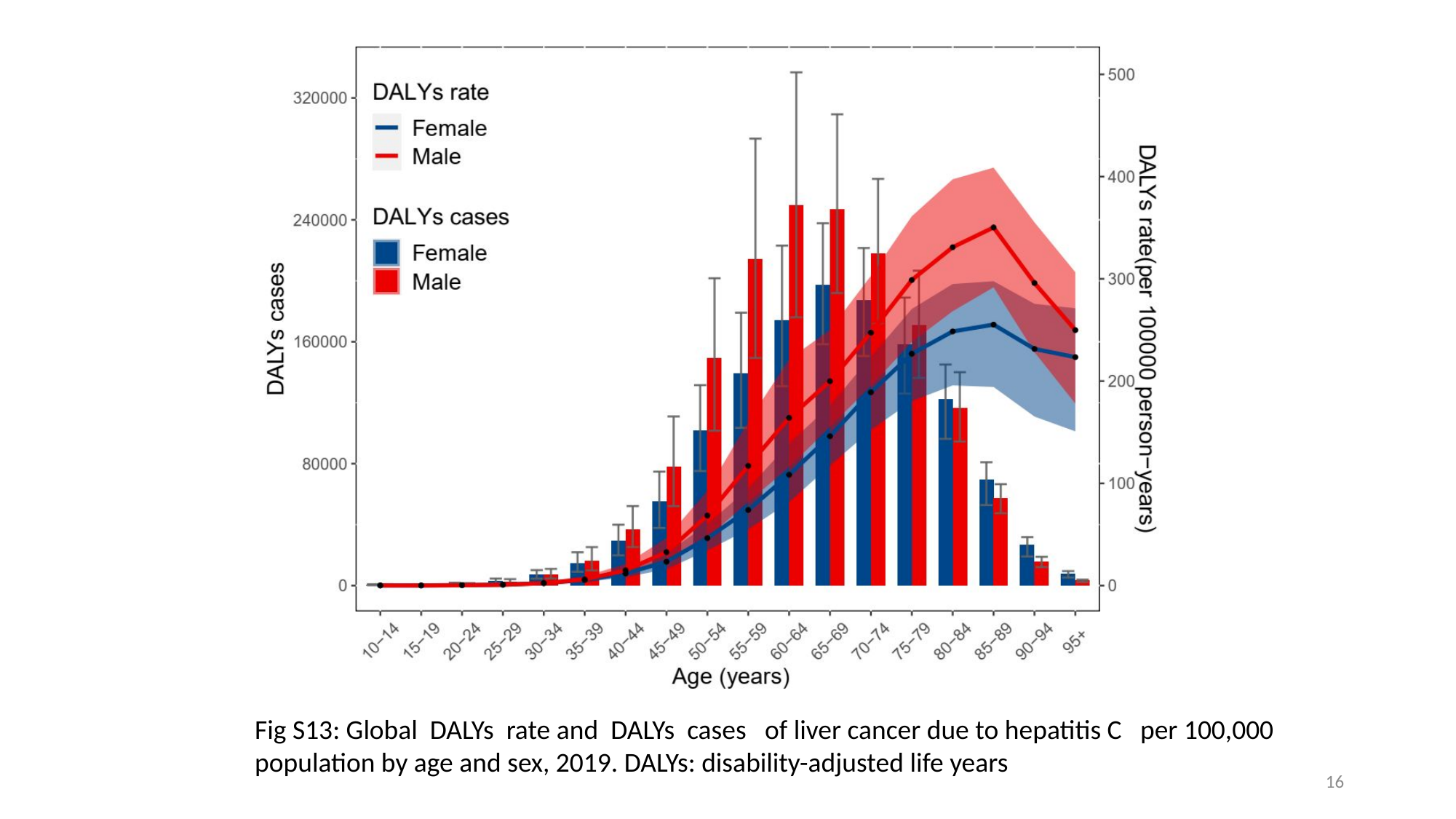

Fig S13: Global DALYs rate and DALYs cases of liver cancer due to hepatitis C per 100,000 population by age and sex, 2019. DALYs: disability-adjusted life years
16

## Slide 17
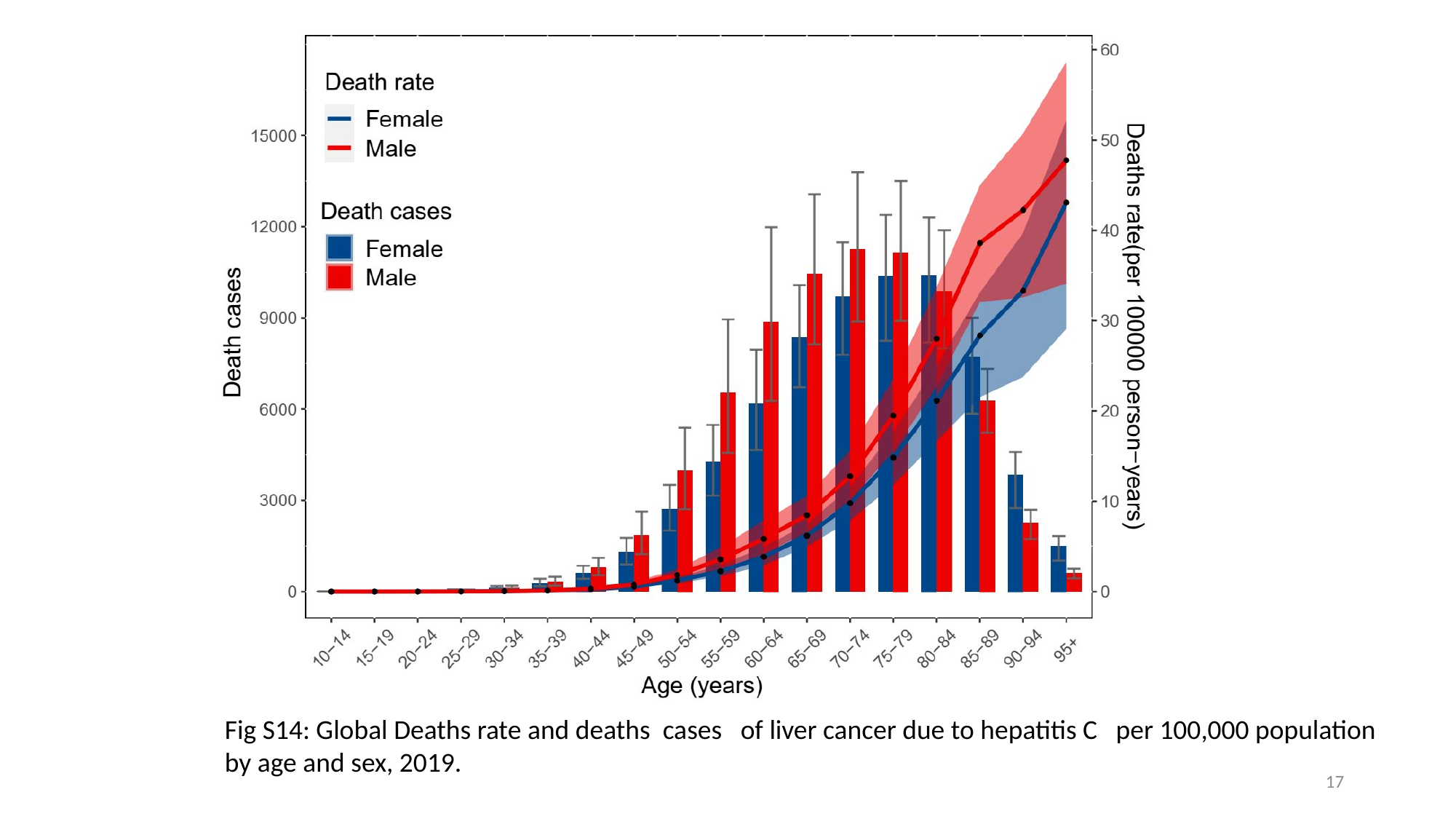

Fig S14: Global Deaths rate and deaths cases of liver cancer due to hepatitis C per 100,000 population by age and sex, 2019.
17

## Slide 18
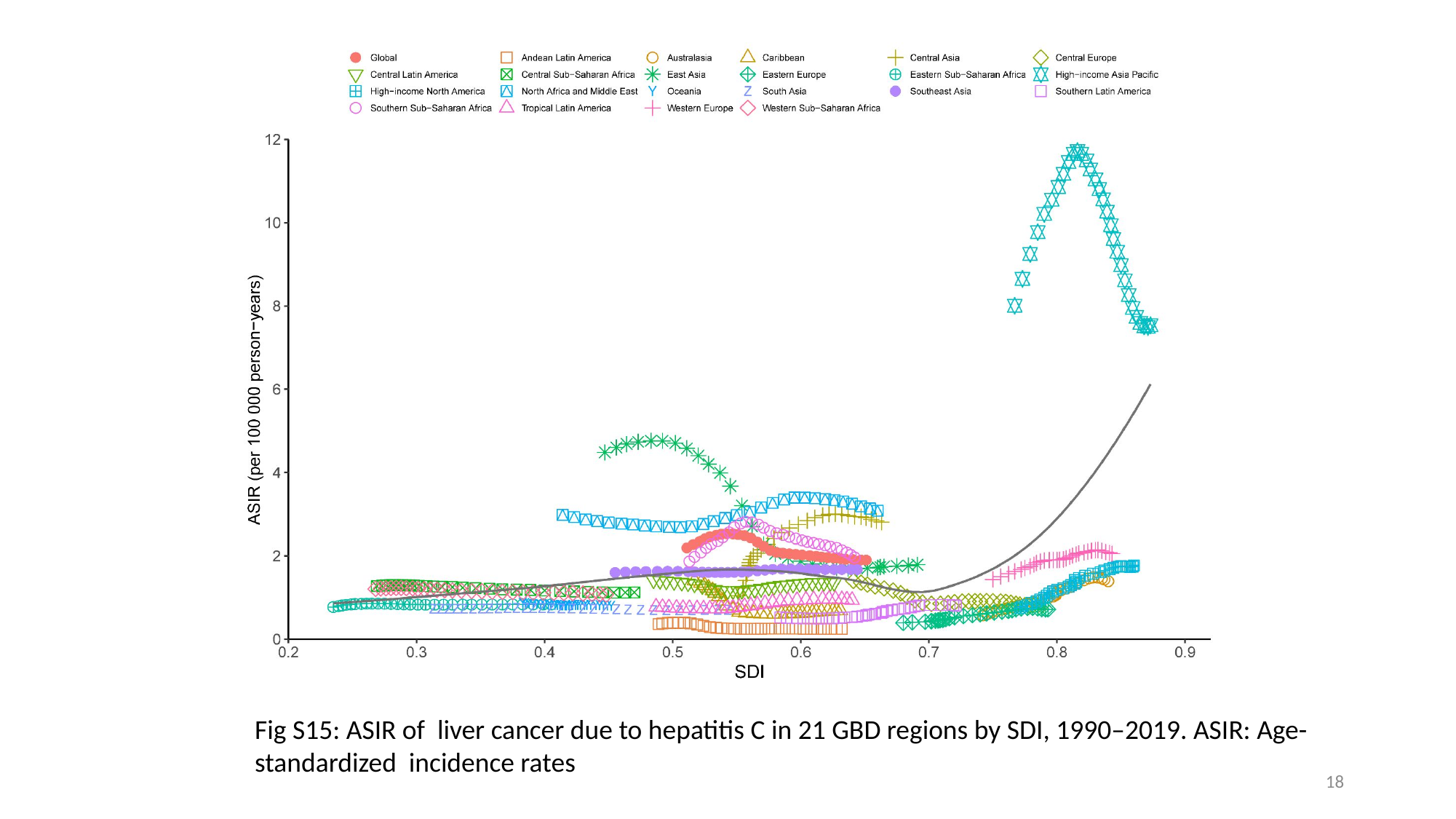

Fig S15: ASIR of liver cancer due to hepatitis C in 21 GBD regions by SDI, 1990–2019. ASIR: Age-standardized incidence rates
18

## Slide 19
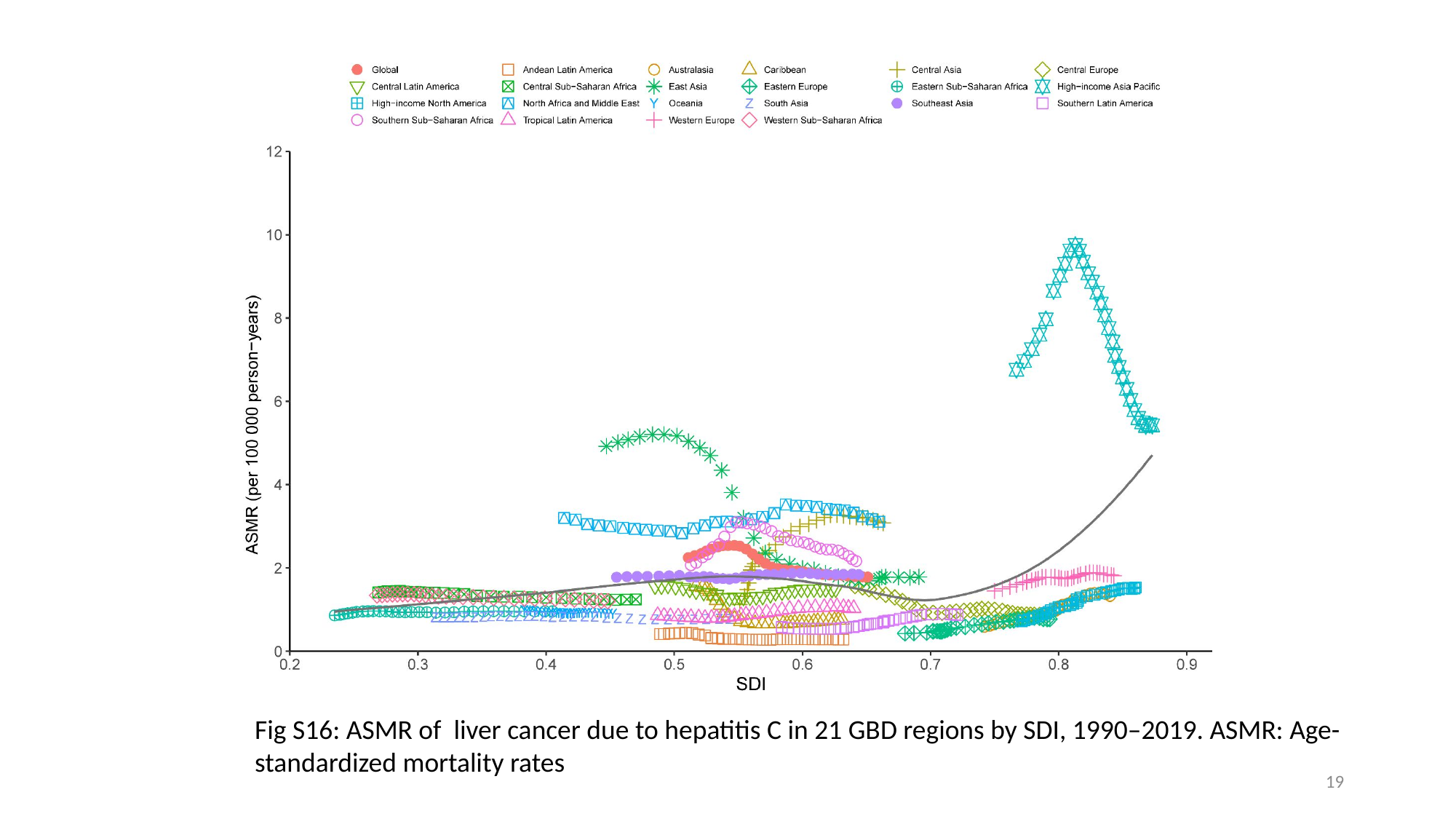

Fig S16: ASMR of liver cancer due to hepatitis C in 21 GBD regions by SDI, 1990–2019. ASMR: Age-standardized mortality rates
19

## Slide 20
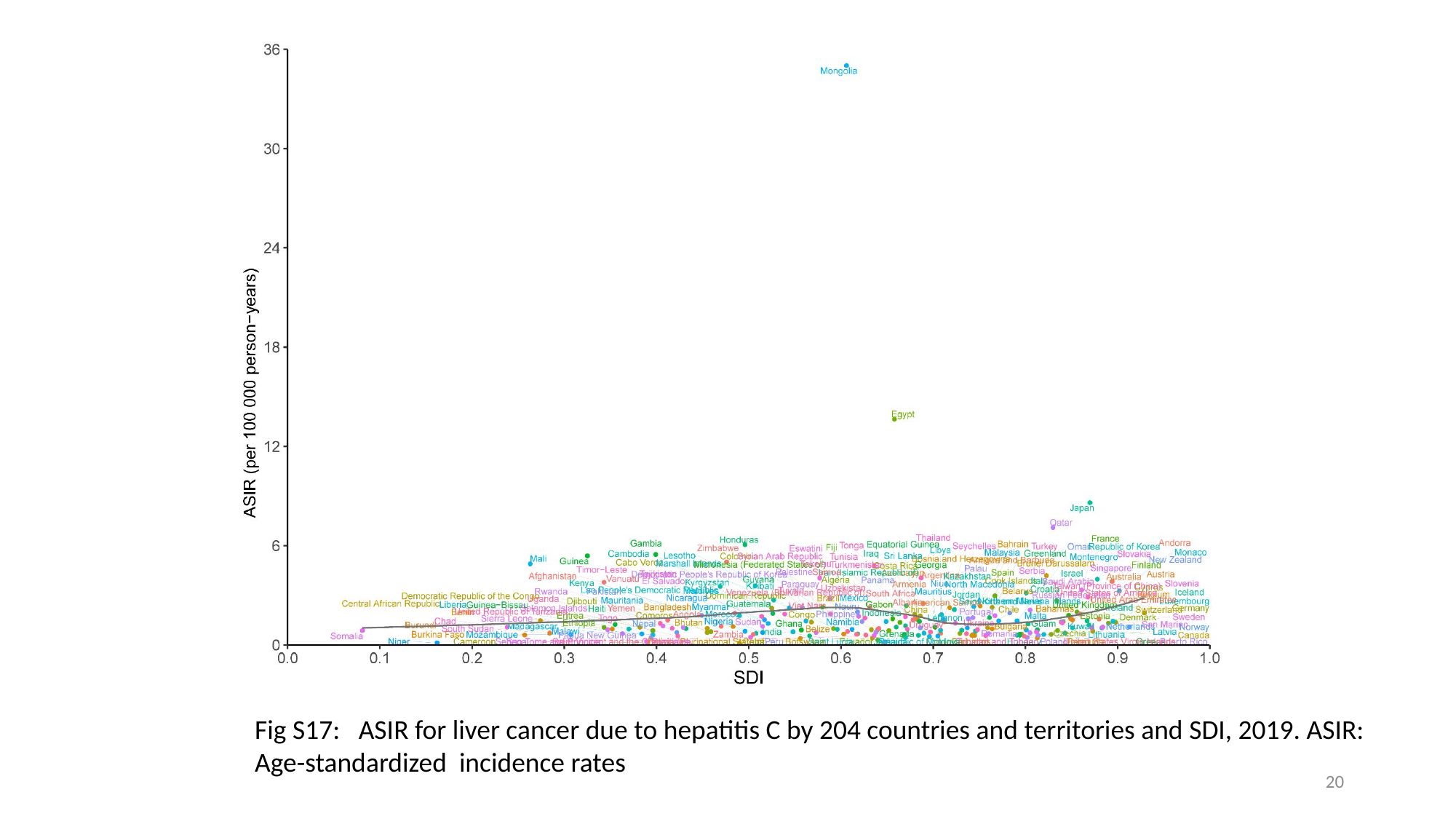

Fig S17: ASIR for liver cancer due to hepatitis C by 204 countries and territories and SDI, 2019. ASIR: Age-standardized incidence rates
20

## Slide 21
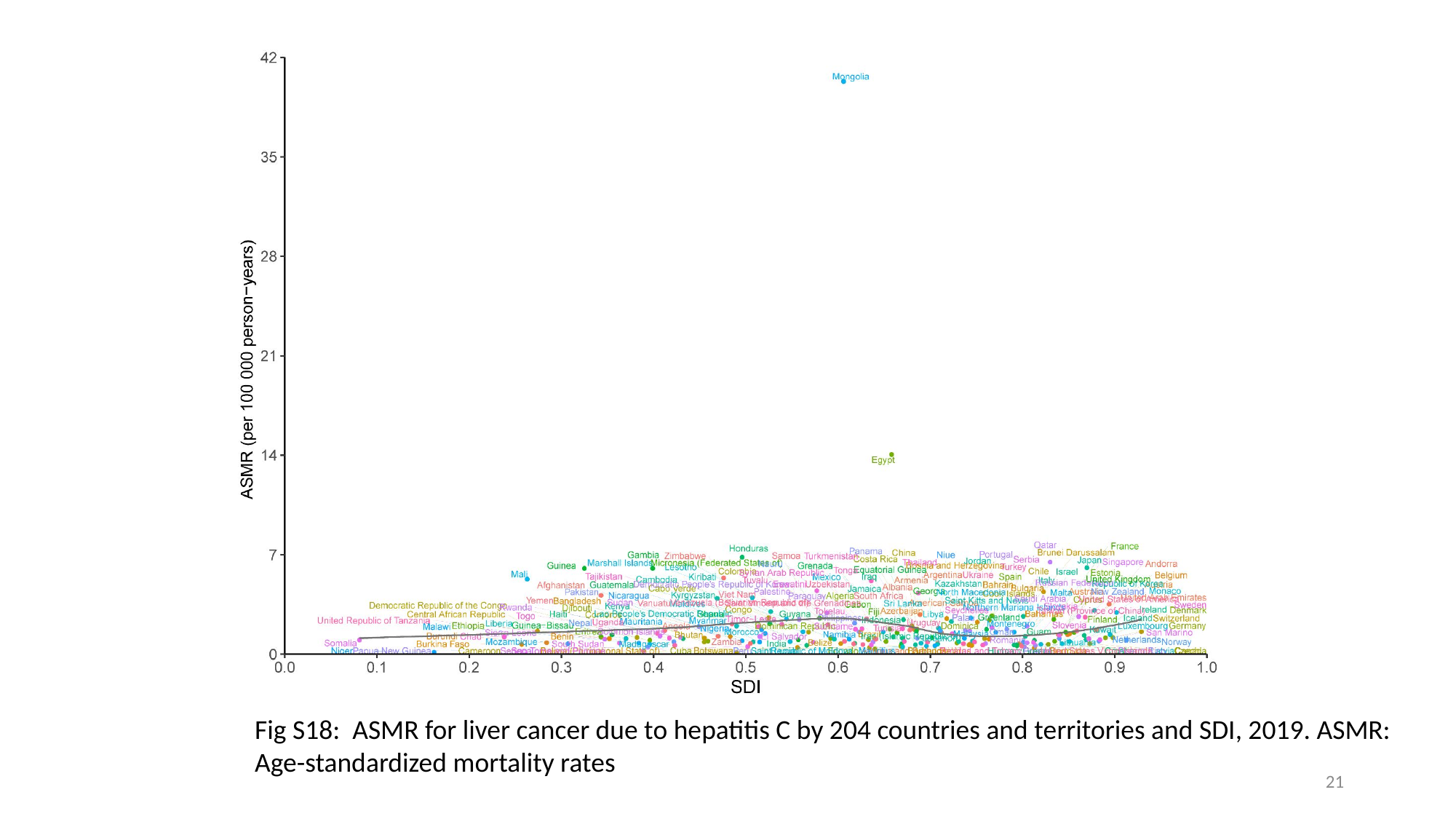

Fig S18: ASMR for liver cancer due to hepatitis C by 204 countries and territories and SDI, 2019. ASMR: Age-standardized mortality rates
21
