# Supplementary material for: Burden of liver cancer due to hepatitis C from 1990 to 2019 at the global, regional, and national levels
Source: Front Oncol. 2023 Dec 19;13:1218901. doi: 10.3389/fonc.2023.1218901 (PMC10760495; doi:10.3389/fonc.2023.1218901)
Supplement: Supplementary file 4 [file Table_1.docx]

**Additional file 1**

**Global Burden of Liver cancer due to hepatitis C from 1990 to 2019 at the global, regional, and national levels**

Jie Wei^1,2,3,#^, Guoqing Ouyang^1,2,3#^, Guozhen Huang^1,2,3^, Yong Wang^1,2,3^, Shuangjiang Li^1,2,3^, Jiaping Liu^1,2,3^, Guandou Yuan^1,2,3,^*, Songqing He^1,2,3,^*

^1^Division of Hepatobiliary Surgery, The First Affiliated Hospital of Guangxi Medical University, Nanning, Guangxi 530021, China;

^2^Key Laboratory of Basic and Clinical Application Research for Hepatobiliary Dis-eases of Guangxi, Nanning, Guangxi 530021, China;

^3^Guangxi Key Laboratory of Immunology and Metabolism for Liver Diseases, Nan-ning, Guangxi 530021, China;

**# Theses authors contributed equally as first author**

***Correspondence：**

Songqing He, Division of Hepatobiliary Surgery, The First Affiliated Hospital of Guangxi Medical University, NO 6 Shuangyong Road, Nanning, Guangxi 530021, China. Email: dr_hesongqing@163.com

Guandou Yuan, Division of Hepatobiliary Surgery, The First Affiliated Hospital of Guangxi Medical University, NO 6 Shuangyong Road, Nanning, Guangxi 530021, China. E-mail: dr_yuangd@gxmu.edu.cn

**Table S1.** **The incidence of liver cancer due to hepatitis C between 1990 and 2019 at national level, both sexes……………..………………………………………………..3-14**

**Table S2. The death of liver cancer due to hepatitis C between 1990 and 2019 at national level, both sexes………………………………………………………………….………16-25**

**Table S3. The disability-adjusted life years (DALYs) of liver cancer due to hepatitis C between 1990 and 2019 at national level, both sexes……………………..……….…..26-36**

| **Table S1. The incidence of liver cancer due to hepatitis C between 1990 and 2019 at national level, both sexes** | | | | | | |
| --- | --- | --- | --- | --- | --- | --- |
| **Region** | **Case in 1990** | **Case in 2019** | **Change in absolute number (95% UI)** | **ASIR in 1990** | **ASIR in 2019** | **change in ASIR per 100 000 population (95% UI)** |
| Afghanistan | 304 (204-428) | 439 (291-622) | 0.44 (0.02-1.04) | 4.43 (3.04-6.04) | 3.79 (2.62-5.31) | -0.14 (-0.37-0.16) |
| Albania | 61 (43-81) | 74 (47-111) | 0.21 (-0.12-0.65) | 3.23 (2.31-4.22) | 1.69 (1.08-2.52) | -0.48 (-0.61--0.29) |
| Algeria | 69 (47-96) | 260 (181-357) | 2.75 (1.66-4.06) | 0.66 (0.47-0.87) | 0.87 (0.61-1.18) | 0.31 (-0.05-0.71) |
| American Samoa | 0 (0-0) | 1 (0-1) | 1.58 (1-2.42) | 1.24 (0.81-1.72) | 1.4 (0.95-1.91) | 0.13 (-0.12-0.47) |
| Andorra | 2 (1-3) | 5 (4-8) | 2.22 (1.11-3.75) | 3.25 (2.12-4.91) | 3.84 (2.5-5.45) | 0.18 (-0.22-0.72) |
| Angola | 43 (30-60) | 110 (77-149) | 1.53 (0.77-2.62) | 1.25 (0.88-1.69) | 1.13 (0.82-1.48) | -0.1 (-0.35-0.24) |
| Antigua and Barbuda | 1 (1-1) | 1 (0-1) | -0.38 (-0.49--0.25) | 1.75 (1.19-2.38) | 0.63 (0.43-0.88) | -0.64 (-0.7--0.57) |
| Argentina | 149 (107-202) | 385 (263-529) | 1.59 (0.99-2.36) | 0.47 (0.34-0.63) | 0.7 (0.48-0.97) | 0.49 (0.14-0.92) |
| Armenia | 10 (7-12) | 103 (73-137) | 9.45 (7.6-11.61) | 0.4 (0.3-0.5) | 2.49 (1.78-3.27) | 5.21 (4.08-6.46) |
| Australia | 110 (79-143) | 581 (388-825) | 4.3 (3.12-5.74) | 0.56 (0.41-0.72) | 1.39 (0.93-1.97) | 1.48 (0.92-2.15) |
| Austria | 119 (88-155) | 289 (198-412) | 1.42 (0.93-2.05) | 0.99 (0.73-1.29) | 1.6 (1.1-2.3) | 0.61 (0.28-1.03) |
| Azerbaijan | 16 (12-21) | 121 (79-178) | 6.37 (4.34-9.23) | 0.36 (0.27-0.45) | 1.52 (1.01-2.26) | 3.25 (2.04-5.09) |
| Bahamas | 2 (2-3) | 2 (2-3) | 0.04 (-0.17-0.31) | 1.61 (1.1-2.21) | 0.66 (0.44-0.91) | -0.59 (-0.67--0.48) |
| Bahrain | 3 (2-4) | 15 (10-21) | 4.12 (2.75-5.89) | 2.23 (1.6-2.91) | 2.35 (1.67-3.14) | 0.05 (-0.22-0.39) |
| Bangladesh | 535 (379-730) | 1384 (973-1845) | 1.59 (0.89-2.54) | 1.19 (0.85-1.61) | 1.11 (0.78-1.46) | -0.07 (-0.31-0.26) |
| Barbados | 2 (1-3) | 3 (2-4) | 0.52 (0.13-1.08) | 0.62 (0.4-0.91) | 0.58 (0.39-0.82) | -0.06 (-0.3-0.29) |
| Belarus | 43 (31-57) | 94 (61-137) | 1.18 (0.65-1.81) | 0.33 (0.24-0.43) | 0.58 (0.37-0.85) | 0.74 (0.31-1.25) |
| Belgium | 176 (133-218) | 356 (248-484) | 1.02 (0.61-1.55) | 1.11 (0.84-1.37) | 1.51 (1.05-2.08) | 0.37 (0.09-0.74) |
| Belize | 1 (1-2) | 2 (1-2) | 0.26 (0.03-0.52) | 1.49 (1.05-2.01) | 0.66 (0.45-0.92) | -0.56 (-0.63--0.46) |
| Benin | 23 (15-34) | 41 (25-58) | 0.74 (0.28-1.42) | 1.26 (0.81-1.78) | 0.96 (0.62-1.35) | -0.24 (-0.43-0.04) |
| Bermuda | 1 (1-1) | 1 (0-1) | -0.37 (-0.49--0.2) | 1.52 (1.04-2.08) | 0.42 (0.28-0.61) | -0.72 (-0.78--0.65) |
| Bhutan | 1 (1-2) | 5 (3-8) | 2.53 (1.43-4.23) | 0.65 (0.39-1.01) | 1 (0.62-1.54) | 0.53 (0.08-1.19) |
| Bolivia (Plurinational State of) | 12 (6-20) | 32 (17-53) | 1.66 (0.89-2.76) | 0.42 (0.23-0.71) | 0.4 (0.22-0.65) | -0.05 (-0.32-0.32) |
| Bosnia and Herzegovina | 59 (40-80) | 137 (93-195) | 1.31 (0.76-1.99) | 1.53 (1.07-2.03) | 2.26 (1.55-3.18) | 0.47 (0.15-0.88) |
| Botswana | 2 (1-3) | 5 (3-7) | 2.23 (0.67-5.25) | 0.31 (0.15-0.6) | 0.41 (0.27-0.59) | 0.34 (-0.3-1.59) |
| Brazil | 640 (570-712) | 2178 (1907-2436) | 2.4 (2.21-2.63) | 0.78 (0.69-0.86) | 0.94 (0.82-1.05) | 0.21 (0.15-0.29) |
| Brunei Darussalam | 3 (2-4) | 11 (7-15) | 3.06 (2.17-4.23) | 3.49 (2.36-4.77) | 4.19 (3.04-5.52) | 0.2 (-0.03-0.53) |
| Bulgaria | 180 (121-246) | 142 (93-209) | -0.21 (-0.38-0) | 1.43 (1-1.92) | 0.97 (0.64-1.41) | -0.32 (-0.46--0.15) |
| Burkina Faso | 33 (22-46) | 48 (32-66) | 0.44 (0.04-0.98) | 0.87 (0.59-1.17) | 0.6 (0.39-0.82) | -0.31 (-0.49--0.07) |
| Burundi | 17 (10-27) | 28 (16-43) | 0.59 (0.04-1.42) | 0.81 (0.5-1.22) | 0.71 (0.44-1.09) | -0.13 (-0.41-0.29) |
| Cabo Verde | 1 (0-1) | 9 (6-13) | 14.77 (11.11-20.32) | 0.24 (0.16-0.33) | 2.22 (1.46-3.17) | 8.35 (6.13-11.63) |
| Cambodia | 181 (125-243) | 392 (274-525) | 1.17 (0.54-2.15) | 4.27 (3.04-5.55) | 3.54 (2.54-4.63) | -0.17 (-0.4-0.19) |
| Cameroon | 5 (3-7) | 11 (7-17) | 1.47 (0.64-2.56) | 0.12 (0.07-0.18) | 0.11 (0.07-0.16) | -0.07 (-0.37-0.31) |
| Canada | 116 (80-157) | 578 (361-852) | 4 (2.83-5.43) | 0.36 (0.25-0.48) | 0.83 (0.52-1.22) | 1.33 (0.79-2) |
| Central African Republic | 17 (10-25) | 28 (16-43) | 0.67 (0.2-1.3) | 1.55 (1.01-2.25) | 1.46 (0.9-2.17) | -0.06 (-0.3-0.26) |
| Chad | 34 (21-49) | 53 (32-78) | 0.55 (0.19-1.08) | 1.28 (0.81-1.81) | 1.07 (0.68-1.54) | -0.16 (-0.35-0.11) |
| Chile | 70 (51-89) | 252 (168-352) | 2.62 (1.8-3.62) | 0.72 (0.52-0.92) | 1.04 (0.69-1.45) | 0.44 (0.13-0.84) |
| China | 35447 (28972-43242) | 34036 (27796-40829) | -0.04 (-0.25-0.21) | 4.53 (3.76-5.48) | 1.75 (1.44-2.09) | -0.61 (-0.69--0.51) |
| Colombia | 177 (134-220) | 489 (337-684) | 1.76 (1.12-2.58) | 1.09 (0.83-1.34) | 0.92 (0.63-1.29) | -0.15 (-0.35-0.1) |
| Comoros | 2 (1-3) | 3 (2-5) | 1.1 (0.46-2.43) | 0.79 (0.45-1.27) | 0.75 (0.48-1.15) | -0.05 (-0.32-0.47) |
| Congo | 19 (12-27) | 32 (21-47) | 0.71 (0.24-1.37) | 1.88 (1.32-2.62) | 1.38 (0.95-1.97) | -0.27 (-0.45--0.02) |
| Cook Islands | 0 (0-0) | 1 (0-1) | 0.62 (0.27-1.09) | 2.96 (1.97-4.05) | 2.27 (1.52-3.21) | -0.23 (-0.39--0.02) |
| Costa Rica | 34 (25-43) | 93 (63-129) | 1.76 (1.14-2.56) | 2.01 (1.51-2.56) | 1.84 (1.24-2.54) | -0.08 (-0.29-0.18) |
| Croatia | 40 (27-55) | 61 (39-89) | 0.54 (0.2-0.99) | 0.63 (0.43-0.87) | 0.67 (0.43-0.96) | 0.05 (-0.17-0.34) |
| Cuba | 158 (111-210) | 101 (66-144) | -0.36 (-0.49--0.23) | 1.53 (1.08-2.03) | 0.52 (0.34-0.75) | -0.66 (-0.73--0.59) |
| Cyprus | 9 (6-12) | 27 (19-34) | 1.91 (1.3-2.85) | 1.22 (0.88-1.6) | 1.41 (1.04-1.82) | 0.16 (-0.07-0.49) |
| Czechia | 133 (91-178) | 141 (93-205) | 0.06 (-0.16-0.33) | 0.95 (0.66-1.27) | 0.65 (0.42-0.94) | -0.32 (-0.45--0.15) |
| Democratic People's Republic of Korea | 477 (299-690) | 717 (461-1019) | 0.5 (0.1-1.03) | 3.21 (2.07-4.53) | 2.27 (1.48-3.21) | -0.29 (-0.48--0.06) |
| Democratic Republic of the Congo | 171 (124-226) | 352 (245-484) | 1.05 (0.44-1.95) | 1.19 (0.9-1.52) | 1.07 (0.76-1.43) | -0.11 (-0.34-0.23) |
| Denmark | 68 (50-88) | 152 (104-215) | 1.25 (0.75-1.85) | 0.82 (0.6-1.05) | 1.32 (0.9-1.86) | 0.62 (0.26-1.06) |
| Djibouti | 1 (1-2) | 4 (2-7) | 3.31 (2.02-5.09) | 0.79 (0.48-1.3) | 0.8 (0.49-1.29) | 0.02 (-0.26-0.37) |
| Dominica | 2 (1-2) | 1 (0-1) | -0.58 (-0.67--0.47) | 2.03 (1.43-2.76) | 0.69 (0.46-0.99) | -0.66 (-0.73--0.56) |
| Dominican Republic | 30 (21-41) | 87 (55-139) | 1.9 (0.91-3.28) | 0.88 (0.62-1.18) | 0.98 (0.62-1.55) | 0.11 (-0.26-0.62) |
| Ecuador | 11 (7-16) | 41 (24-62) | 2.64 (1.68-3.76) | 0.23 (0.15-0.34) | 0.29 (0.18-0.44) | 0.26 (-0.06-0.66) |
| Egypt | 3294 (2484-4067) | 8678 (5840-12411) | 1.63 (0.72-3.16) | 11.3 (8.43-13.9) | 13.64 (9.44-19.19) | 0.21 (-0.2-0.89) |
| El Salvador | 40 (30-50) | 45 (30-63) | 0.14 (-0.16-0.51) | 1.38 (1.05-1.73) | 0.75 (0.5-1.06) | -0.45 (-0.6--0.28) |
| Equatorial Guinea | 3 (2-4) | 6 (3-9) | 1.18 (0.16-2.5) | 1.47 (1.02-2.03) | 1.42 (0.81-2.18) | -0.03 (-0.48-0.52) |
| Eritrea | 7 (4-12) | 19 (11-29) | 1.73 (0.83-3.26) | 0.83 (0.47-1.38) | 0.87 (0.54-1.28) | 0.04 (-0.29-0.58) |
| Estonia | 11 (8-15) | 24 (16-34) | 1.18 (0.66-1.8) | 0.54 (0.39-0.72) | 0.87 (0.56-1.26) | 0.62 (0.22-1.04) |
| Eswatini | 4 (3-8) | 22 (7-41) | 3.89 (0.26-9.8) | 1.69 (1.03-2.9) | 4.04 (1.43-7.46) | 1.4 (-0.34-4.11) |
| Ethiopia | 191 (130-272) | 384 (302-492) | 1.01 (0.25-2.09) | 1.09 (0.77-1.51) | 1.06 (0.83-1.35) | -0.03 (-0.36-0.44) |
| Fiji | 4 (3-6) | 9 (6-14) | 1.2 (0.57-2.21) | 1.34 (0.88-1.91) | 1.36 (0.91-1.97) | 0.01 (-0.25-0.44) |
| Finland | 90 (67-114) | 258 (181-359) | 1.87 (1.24-2.73) | 1.23 (0.93-1.56) | 2 (1.39-2.76) | 0.62 (0.27-1.1) |
| France | 1545 (1143-2013) | 3645 (2536-5003) | 1.36 (0.8-2.08) | 1.87 (1.39-2.46) | 2.68 (1.84-3.71) | 0.43 (0.07-0.88) |
| Gabon | 9 (6-12) | 15 (9-23) | 0.7 (0.1-1.6) | 1.67 (1.17-2.22) | 1.58 (1.02-2.33) | -0.05 (-0.38-0.45) |
| Gambia | 13 (8-19) | 49 (31-73) | 2.79 (1.54-4.49) | 4.03 (2.54-5.88) | 5.47 (3.43-8.11) | 0.36 (-0.07-0.9) |
| Georgia | 24 (18-31) | 65 (46-87) | 1.68 (1.18-2.27) | 0.41 (0.31-0.52) | 1.07 (0.76-1.44) | 1.62 (1.11-2.24) |
| Germany | 957 (735-1207) | 2725 (1845-3793) | 1.85 (1.17-2.68) | 0.74 (0.57-0.93) | 1.37 (0.93-1.9) | 0.86 (0.4-1.4) |
| Ghana | 57 (35-83) | 128 (78-190) | 1.27 (0.56-2.2) | 1.03 (0.66-1.49) | 0.9 (0.58-1.31) | -0.12 (-0.4-0.23) |
| Greece | 65 (45-88) | 158 (104-229) | 1.44 (0.9-2.13) | 0.42 (0.3-0.57) | 0.59 (0.38-0.86) | 0.39 (0.09-0.75) |
| Greenland | 0 (0-1) | 1 (1-2) | 1.84 (1.11-2.75) | 1.19 (0.82-1.63) | 1.65 (1.09-2.39) | 0.38 (0.06-0.79) |
| Grenada | 2 (1-2) | 1 (0-1) | -0.57 (-0.64--0.48) | 2.09 (1.45-2.8) | 0.65 (0.44-0.89) | -0.69 (-0.74--0.63) |
| Guam | 1 (0-1) | 2 (1-3) | 2.35 (1.57-3.32) | 0.82 (0.55-1.13) | 0.91 (0.59-1.34) | 0.11 (-0.14-0.41) |
| Guatemala | 132 (99-167) | 200 (142-273) | 0.51 (0.16-0.97) | 4.03 (3.07-5) | 1.9 (1.37-2.58) | -0.53 (-0.63--0.4) |
| Guinea | 165 (106-234) | 272 (165-405) | 0.65 (0.16-1.25) | 5.35 (3.51-7.46) | 5.38 (3.29-8) | 0.01 (-0.28-0.36) |
| Guinea-Bissau | 5 (3-9) | 8 (5-12) | 0.4 (0.02-0.93) | 1.49 (0.9-2.33) | 1.24 (0.75-1.89) | -0.17 (-0.38-0.14) |
| Guyana | 5 (3-7) | 4 (2-6) | -0.24 (-0.44--0.02) | 1.46 (1-2.03) | 0.67 (0.43-0.96) | -0.54 (-0.65--0.4) |
| Haiti | 49 (26-79) | 61 (33-99) | 0.25 (-0.11-0.74) | 1.69 (0.95-2.74) | 0.98 (0.53-1.56) | -0.42 (-0.59--0.2) |
| Honduras | 86 (29-137) | 343 (140-558) | 2.98 (2.05-4.82) | 4.48 (1.48-7.06) | 6.06 (2.5-9.75) | 0.35 (0.04-1) |
| Hungary | 220 (153-292) | 119 (78-168) | -0.46 (-0.56--0.34) | 1.49 (1.05-1.95) | 0.59 (0.39-0.84) | -0.6 (-0.68--0.51) |
| Iceland | 2 (2-3) | 7 (5-9) | 2.08 (1.56-2.71) | 0.76 (0.57-0.97) | 1.2 (0.87-1.59) | 0.58 (0.32-0.9) |
| India | 1935 (1517-2422) | 5842 (4521-7313) | 2.02 (1.42-2.68) | 0.51 (0.4-0.63) | 0.55 (0.43-0.69) | 0.08 (-0.13-0.31) |
| Indonesia | 1015 (856-1211) | 2002 (1634-2333) | 0.97 (0.54-1.43) | 1.16 (0.98-1.39) | 1.08 (0.89-1.23) | -0.07 (-0.27-0.14) |
| Iran (Islamic Republic of) | 258 (198-340) | 764 (649-891) | 1.96 (1.14-2.97) | 1.26 (0.96-1.66) | 1.16 (0.98-1.35) | -0.08 (-0.34-0.26) |
| Iraq | 120 (82-166) | 490 (330-682) | 3.08 (1.88-4.87) | 1.65 (1.14-2.26) | 2.37 (1.64-3.28) | 0.43 (0.02-1.02) |
| Ireland | 23 (17-29) | 111 (75-154) | 3.89 (2.75-5.32) | 0.55 (0.42-0.68) | 1.46 (0.99-2.03) | 1.65 (1.05-2.43) |
| Israel | 56 (42-70) | 148 (105-204) | 1.66 (1.07-2.44) | 1.14 (0.87-1.42) | 1.27 (0.9-1.74) | 0.11 (-0.14-0.44) |
| Italy | 3109 (2833-3394) | 4438 (3568-5463) | 0.43 (0.17-0.71) | 3.45 (3.14-3.77) | 3.14 (2.5-3.92) | -0.09 (-0.25-0.1) |
| Jamaica | 16 (11-21) | 18 (12-25) | 0.08 (-0.17-0.37) | 0.9 (0.62-1.17) | 0.59 (0.38-0.85) | -0.35 (-0.5--0.17) |
| Japan | 15780 (14838-16676) | 33312 (26824-39444) | 1.11 (0.73-1.47) | 9.14 (8.59-9.66) | 8.6 (7.06-10.25) | -0.06 (-0.21-0.1) |
| Jordan | 12 (8-17) | 48 (33-66) | 3 (1.83-4.71) | 1.1 (0.72-1.51) | 0.89 (0.61-1.2) | -0.19 (-0.42-0.17) |
| Kazakhstan | 140 (102-181) | 356 (247-483) | 1.54 (1.15-1.98) | 1.14 (0.84-1.46) | 2.14 (1.5-2.86) | 0.87 (0.59-1.19) |
| Kenya | 44 (30-76) | 133 (87-195) | 2.04 (1.45-2.78) | 0.59 (0.41-1.01) | 0.7 (0.47-1.01) | 0.19 (-0.04-0.45) |
| Kiribati | 1 (1-2) | 2 (1-2) | 0.61 (0.12-1.28) | 3.07 (2.06-4.33) | 2.72 (1.75-3.93) | -0.11 (-0.37-0.24) |
| Kuwait | 5 (3-6) | 21 (15-29) | 3.34 (2.29-4.51) | 0.96 (0.69-1.22) | 1.03 (0.73-1.39) | 0.08 (-0.17-0.36) |
| Kyrgyzstan | 14 (10-18) | 39 (28-51) | 1.84 (1.33-2.41) | 0.47 (0.35-0.59) | 0.95 (0.68-1.24) | 1.01 (0.66-1.4) |
| Lao People's Democratic Republic | 52 (30-82) | 69 (43-98) | 0.31 (-0.11-0.94) | 2.71 (1.61-4.1) | 1.77 (1.15-2.48) | -0.35 (-0.55--0.06) |
| Latvia | 16 (11-21) | 27 (18-37) | 0.71 (0.36-1.1) | 0.43 (0.31-0.57) | 0.64 (0.43-0.89) | 0.48 (0.18-0.81) |
| Lebanon | 16 (10-24) | 46 (29-72) | 1.85 (0.84-3.68) | 0.78 (0.5-1.14) | 0.88 (0.55-1.38) | 0.13 (-0.25-0.79) |
| Lesotho | 14 (8-26) | 42 (19-71) | 1.92 (-0.02-5.03) | 1.58 (0.94-2.84) | 3.57 (1.68-5.91) | 1.27 (-0.2-3.59) |
| Liberia | 13 (8-19) | 17 (10-26) | 0.31 (-0.06-0.86) | 1.24 (0.81-1.75) | 0.96 (0.57-1.44) | -0.23 (-0.43-0.07) |
| Libya | 31 (21-46) | 87 (57-128) | 1.76 (0.75-3.14) | 1.82 (1.19-2.64) | 1.83 (1.2-2.66) | 0.01 (-0.35-0.51) |
| Lithuania | 20 (14-26) | 43 (28-58) | 1.14 (0.68-1.63) | 0.44 (0.31-0.57) | 0.71 (0.47-0.98) | 0.63 (0.28-1.01) |
| Luxembourg | 6 (4-7) | 15 (10-21) | 1.59 (1.02-2.31) | 1.02 (0.75-1.32) | 1.43 (0.98-2.06) | 0.4 (0.1-0.81) |
| Madagascar | 31 (19-50) | 56 (34-87) | 0.85 (0.29-1.62) | 0.65 (0.4-1.05) | 0.61 (0.39-0.93) | -0.06 (-0.33-0.31) |
| Malawi | 25 (16-39) | 42 (27-58) | 0.63 (0.18-1.29) | 0.73 (0.46-1.08) | 0.68 (0.45-0.93) | -0.08 (-0.32-0.26) |
| Malaysia | 80 (51-114) | 252 (159-378) | 2.14 (1.34-3.33) | 0.97 (0.62-1.36) | 1.03 (0.67-1.53) | 0.07 (-0.21-0.45) |
| Maldives | 1 (1-2) | 4 (3-5) | 1.95 (0.81-3.76) | 1.76 (1-2.92) | 1.44 (0.95-2.01) | -0.18 (-0.46-0.27) |
| Mali | 210 (141-293) | 409 (263-588) | 0.94 (0.43-1.57) | 5.14 (3.54-6.98) | 4.9 (3.25-6.81) | -0.05 (-0.29-0.24) |
| Malta | 3 (2-3) | 9 (6-12) | 2.17 (1.59-2.91) | 0.64 (0.48-0.81) | 0.91 (0.66-1.22) | 0.43 (0.17-0.75) |
| Marshall Islands | 0 (0-1) | 1 (0-1) | 0.77 (0.25-1.47) | 2.6 (1.64-3.93) | 2.24 (1.36-3.33) | -0.14 (-0.38-0.16) |
| Mauritania | 13 (8-18) | 16 (10-23) | 0.23 (-0.1-0.65) | 1.35 (0.84-1.93) | 0.83 (0.53-1.19) | -0.39 (-0.54--0.18) |
| Mauritius | 3 (2-4) | 9 (6-13) | 1.67 (1.04-2.47) | 0.5 (0.36-0.64) | 0.54 (0.35-0.77) | 0.08 (-0.17-0.39) |
| Mexico | 383 (344-420) | 1595 (1337-1895) | 3.17 (2.61-3.79) | 0.98 (0.88-1.07) | 1.42 (1.19-1.68) | 0.45 (0.26-0.66) |
| Micronesia (Federated States of) | 1 (1-2) | 1 (1-2) | 0.36 (-0.12-1.03) | 2.5 (1.56-3.74) | 2.29 (1.37-3.46) | -0.09 (-0.38-0.34) |
| Monaco | 1 (1-1) | 3 (2-4) | 2.47 (1.58-3.75) | 1.26 (0.85-1.74) | 3.28 (2.27-4.41) | 1.61 (0.91-2.55) |
| Mongolia | 206 (138-288) | 657 (435-925) | 2.19 (1.35-3.41) | 20.42 (13.9-28.35) | 35.02 (24.73-46.77) | 0.71 (0.28-1.35) |
| Montenegro | 9 (6-12) | 14 (10-21) | 0.69 (0.27-1.35) | 1.4 (0.94-1.92) | 1.46 (0.99-2.05) | 0.04 (-0.22-0.45) |
| Morocco | 81 (53-114) | 216 (144-297) | 1.66 (0.94-2.72) | 0.7 (0.46-0.95) | 0.8 (0.53-1.08) | 0.14 (-0.16-0.57) |
| Mozambique | 20 (12-32) | 55 (33-82) | 1.75 (0.49-3.31) | 0.41 (0.25-0.66) | 0.62 (0.38-0.92) | 0.51 (-0.17-1.34) |
| Myanmar | 182 (108-291) | 548 (373-745) | 2.01 (1.1-3.4) | 0.9 (0.57-1.39) | 1.3 (0.9-1.73) | 0.44 (0.04-1.1) |
| Namibia | 4 (2-7) | 12 (8-17) | 2.02 (0.85-3.9) | 0.61 (0.38-0.99) | 0.94 (0.63-1.3) | 0.56 (-0.03-1.46) |
| Nauru | 0 (0-0) | 0 (0-0) | -0.05 (-0.33-0.32) | 2.29 (1.47-3.27) | 1.99 (1.24-3.04) | -0.13 (-0.35-0.16) |
| Nepal | 49 (32-72) | 148 (98-232) | 2.03 (1.06-3.19) | 0.61 (0.41-0.87) | 0.75 (0.51-1.14) | 0.23 (-0.16-0.7) |
| Netherlands | 91 (68-117) | 355 (247-495) | 2.9 (2.07-3.87) | 0.45 (0.34-0.58) | 1.03 (0.71-1.43) | 1.26 (0.77-1.85) |
| New Zealand | 25 (21-28) | 108 (87-134) | 3.42 (2.63-4.31) | 0.63 (0.55-0.73) | 1.39 (1.12-1.72) | 1.21 (0.82-1.66) |
| Nicaragua | 18 (13-23) | 62 (44-84) | 2.53 (1.75-3.43) | 1.22 (0.89-1.56) | 1.52 (1.1-2.02) | 0.25 (-0.03-0.58) |
| Niger | 3 (2-5) | 9 (5-13) | 1.7 (1-2.62) | 0.13 (0.09-0.19) | 0.13 (0.08-0.18) | -0.04 (-0.27-0.27) |
| Nigeria | 307 (216-419) | 558 (421-718) | 0.82 (0.31-1.56) | 0.78 (0.56-1.06) | 0.75 (0.58-0.95) | -0.04 (-0.3-0.36) |
| Niue | 0 (0-0) | 0 (0-0) | -0.17 (-0.4-0.12) | 1.81 (1.21-2.58) | 1.55 (1.01-2.25) | -0.14 (-0.37-0.16) |
| North Macedonia | 41 (28-56) | 73 (48-105) | 0.76 (0.35-1.29) | 2.3 (1.6-3.07) | 2.32 (1.56-3.27) | 0.01 (-0.22-0.3) |
| Northern Mariana Islands | 0 (0-0) | 1 (0-1) | 2.3 (1.44-3.41) | 1.5 (0.99-2.08) | 1.45 (0.93-2.02) | -0.03 (-0.24-0.22) |
| Norway | 43 (38-48) | 104 (84-126) | 1.43 (1.03-1.87) | 0.62 (0.55-0.69) | 1.08 (0.87-1.32) | 0.74 (0.46-1.06) |
| Oman | 10 (6-14) | 27 (18-37) | 1.87 (0.99-3.23) | 1.62 (1.04-2.33) | 1.93 (1.35-2.54) | 0.19 (-0.18-0.79) |
| Pakistan | 1034 (662-1434) | 1869 (1400-2382) | 0.81 (0.29-1.66) | 1.86 (1.18-2.58) | 1.79 (1.31-2.24) | -0.04 (-0.3-0.43) |
| Palau | 0 (0-0) | 0 (0-0) | 1.22 (0.48-2.39) | 1.59 (0.96-2.41) | 1.59 (1.03-2.27) | 0 (-0.32-0.48) |
| Palestine | 28 (19-40) | 55 (39-73) | 0.95 (0.34-1.75) | 3.61 (2.47-5) | 2.79 (2.04-3.63) | -0.23 (-0.47-0.09) |
| Panama | 18 (13-23) | 43 (29-60) | 1.4 (0.81-2.16) | 1.23 (0.92-1.57) | 1.04 (0.7-1.48) | -0.16 (-0.37-0.11) |
| Papua New Guinea | 6 (4-8) | 15 (10-23) | 1.63 (0.91-2.63) | 0.41 (0.26-0.58) | 0.44 (0.29-0.64) | 0.08 (-0.2-0.44) |
| Paraguay | 18 (13-24) | 40 (27-56) | 1.2 (0.62-1.98) | 0.87 (0.63-1.12) | 0.77 (0.52-1.05) | -0.12 (-0.35-0.19) |
| Peru | 45 (27-67) | 60 (36-96) | 0.35 (-0.07-0.93) | 0.41 (0.25-0.6) | 0.19 (0.11-0.3) | -0.53 (-0.67--0.34) |
| Philippines | 547 (400-703) | 1022 (806-1295) | 0.87 (0.4-1.67) | 2.07 (1.54-2.62) | 1.42 (1.13-1.78) | -0.31 (-0.48--0.04) |
| Poland | 857 (743-974) | 333 (269-409) | -0.61 (-0.67--0.54) | 2.02 (1.76-2.27) | 0.46 (0.37-0.56) | -0.77 (-0.81--0.73) |
| Portugal | 92 (69-117) | 388 (268-539) | 3.23 (2.29-4.39) | 0.66 (0.51-0.83) | 1.64 (1.11-2.29) | 1.49 (0.92-2.17) |
| Puerto Rico | 54 (37-73) | 44 (28-64) | -0.19 (-0.38-0.04) | 1.48 (1.03-1.96) | 0.58 (0.37-0.85) | -0.61 (-0.7--0.49) |
| Qatar | 4 (3-6) | 31 (19-46) | 6.34 (3.97-9.8) | 6.25 (4.02-8.87) | 7.09 (4.79-9.84) | 0.13 (-0.21-0.6) |
| Republic of Korea | 476 (314-679) | 3549 (2361-4874) | 6.45 (4.35-9.32) | 1.81 (1.23-2.55) | 3.98 (2.69-5.45) | 1.19 (0.61-1.97) |
| Republic of Moldova | 14 (10-19) | 30 (20-43) | 1.13 (0.76-1.53) | 0.33 (0.24-0.45) | 0.52 (0.35-0.73) | 0.55 (0.29-0.82) |
| Romania | 118 (81-159) | 261 (175-365) | 1.22 (0.77-1.73) | 0.43 (0.31-0.57) | 0.69 (0.46-0.97) | 0.59 (0.27-0.95) |
| Russian Federation | 767 (657-878) | 1757 (1418-2117) | 1.29 (1.01-1.62) | 0.43 (0.37-0.49) | 0.74 (0.6-0.89) | 0.71 (0.49-0.95) |
| Rwanda | 30 (18-48) | 55 (37-79) | 0.85 (0.21-1.83) | 1.15 (0.7-1.75) | 1.05 (0.72-1.48) | -0.08 (-0.37-0.35) |
| Saint Kitts and Nevis | 1 (1-2) | 1 (0-1) | -0.57 (-0.66--0.46) | 3.06 (2.15-4.07) | 0.84 (0.56-1.18) | -0.73 (-0.77--0.67) |
| Saint Lucia | 1 (1-2) | 1 (1-1) | -0.18 (-0.33--0.01) | 1.37 (0.98-1.82) | 0.45 (0.3-0.64) | -0.67 (-0.73--0.6) |
| Saint Vincent and the Grenadines | 1 (1-2) | 1 (1-1) | -0.33 (-0.44--0.22) | 1.68 (1.19-2.23) | 0.6 (0.41-0.84) | -0.64 (-0.7--0.59) |
| Samoa | 1 (1-1) | 1 (1-2) | 0.4 (0.03-0.87) | 1.18 (0.77-1.73) | 0.99 (0.63-1.45) | -0.16 (-0.37-0.1) |
| San Marino | 0 (0-0) | 1 (0-1) | 1.74 (0.96-2.79) | 0.77 (0.55-1.03) | 1.08 (0.73-1.55) | 0.4 (0.01-0.96) |
| Sao Tome and Principe | 0 (0-0) | 0 (0-1) | 0.54 (0.06-1.2) | 0.47 (0.31-0.65) | 0.47 (0.28-0.72) | 0 (-0.31-0.39) |
| Saudi Arabia | 96 (62-134) | 284 (188-399) | 1.97 (0.94-3.42) | 1.91 (1.25-2.64) | 2.11 (1.46-2.88) | 0.11 (-0.26-0.66) |
| Senegal | 6 (4-9) | 13 (8-21) | 1.2 (0.54-2.05) | 0.21 (0.13-0.31) | 0.2 (0.12-0.31) | -0.05 (-0.32-0.3) |
| Serbia | 212 (145-294) | 288 (194-405) | 0.36 (0-0.81) | 1.93 (1.37-2.65) | 1.76 (1.22-2.43) | -0.09 (-0.31-0.21) |
| Seychelles | 1 (1-1) | 1 (1-2) | 0.27 (0.03-0.55) | 1.91 (1.32-2.6) | 1.32 (0.9-1.79) | -0.31 (-0.43--0.16) |
| Sierra Leone | 20 (12-29) | 30 (18-43) | 0.5 (0.06-1.1) | 1.09 (0.7-1.59) | 0.93 (0.58-1.34) | -0.15 (-0.39-0.18) |
| Singapore | 41 (27-56) | 246 (156-355) | 5.05 (3.8-6.78) | 2.02 (1.37-2.74) | 3.32 (2.11-4.72) | 0.65 (0.31-1.1) |
| Slovakia | 60 (42-81) | 73 (47-107) | 0.22 (-0.07-0.58) | 1 (0.7-1.34) | 0.79 (0.51-1.14) | -0.21 (-0.4-0.01) |
| Slovenia | 18 (12-27) | 60 (39-86) | 2.34 (1.29-3.63) | 0.73 (0.48-1.08) | 1.33 (0.86-1.92) | 0.82 (0.25-1.55) |
| Solomon Islands | 2 (1-3) | 3 (2-4) | 0.85 (0.29-1.7) | 1.32 (0.8-1.94) | 1.15 (0.77-1.61) | -0.13 (-0.37-0.24) |
| Somalia | 19 (10-32) | 48 (27-85) | 1.57 (0.73-2.77) | 0.86 (0.49-1.43) | 0.87 (0.51-1.56) | 0.01 (-0.29-0.45) |
| South Africa | 317 (203-492) | 630 (534-751) | 0.99 (0.24-2.01) | 1.61 (1.02-2.49) | 1.51 (1.29-1.81) | -0.06 (-0.41-0.44) |
| South Sudan | 16 (9-27) | 23 (12-39) | 0.42 (0-0.97) | 0.74 (0.43-1.26) | 0.68 (0.39-1.15) | -0.08 (-0.33-0.25) |
| Spain | 1041 (823-1278) | 2839 (2022-3751) | 1.73 (1.11-2.49) | 1.88 (1.49-2.3) | 2.97 (2.07-3.98) | 0.58 (0.22-1.03) |
| Sri Lanka | 50 (33-68) | 173 (110-259) | 2.47 (1.45-3.9) | 0.53 (0.37-0.71) | 0.72 (0.46-1.04) | 0.34 (-0.04-0.87) |
| Sudan | 95 (51-147) | 182 (102-292) | 0.92 (0.32-1.78) | 1.12 (0.61-1.73) | 1.12 (0.64-1.78) | 0 (-0.3-0.44) |
| Suriname | 4 (2-5) | 3 (2-5) | -0.09 (-0.29-0.15) | 1.45 (1.01-1.96) | 0.56 (0.38-0.8) | -0.61 (-0.69--0.51) |
| Sweden | 141 (120-163) | 217 (172-266) | 0.54 (0.32-0.79) | 0.92 (0.78-1.07) | 1.04 (0.82-1.28) | 0.13 (-0.04-0.31) |
| Switzerland | 88 (63-115) | 340 (229-478) | 2.88 (2.01-4.14) | 0.83 (0.6-1.09) | 1.93 (1.29-2.73) | 1.33 (0.79-2.07) |
| Syrian Arab Republic | 83 (54-118) | 186 (124-264) | 1.24 (0.5-2.43) | 1.8 (1.18-2.55) | 1.73 (1.18-2.42) | -0.04 (-0.36-0.47) |
| Taiwan (Province of China) | 501 (365-646) | 1159 (796-1593) | 1.31 (0.77-2.02) | 3.16 (2.32-3.99) | 2.9 (1.97-4) | -0.08 (-0.29-0.19) |
| Tajikistan | 13 (10-16) | 75 (55-101) | 4.91 (3.53-6.58) | 0.48 (0.37-0.59) | 1.86 (1.39-2.44) | 2.91 (2.03-3.97) |
| Thailand | 1220 (816-1710) | 4113 (2573-6175) | 2.37 (1.43-3.64) | 3.79 (2.6-5.22) | 4.06 (2.57-6.04) | 0.07 (-0.22-0.46) |
| Timor-Leste | 5 (3-8) | 13 (8-20) | 1.56 (0.74-2.8) | 2.08 (1.3-3.15) | 1.73 (1.12-2.52) | -0.17 (-0.41-0.16) |
| Togo | 14 (9-20) | 31 (19-45) | 1.25 (0.62-2.15) | 1.26 (0.83-1.76) | 1.01 (0.65-1.42) | -0.2 (-0.41-0.1) |
| Tokelau | 0 (0-0) | 0 (0-0) | -0.25 (-0.47-0.06) | 2.05 (1.31-2.92) | 1.63 (1.06-2.31) | -0.21 (-0.44-0.12) |
| Tonga | 2 (2-4) | 4 (2-5) | 0.51 (0.11-1.02) | 4.66 (2.95-6.94) | 4.77 (3.02-7.04) | 0.02 (-0.24-0.36) |
| Trinidad and Tobago | 13 (9-17) | 10 (6-15) | -0.19 (-0.4-0.07) | 1.59 (1.11-2.09) | 0.57 (0.36-0.83) | -0.64 (-0.73--0.54) |
| Tunisia | 41 (28-56) | 115 (77-168) | 1.8 (0.84-3.45) | 0.88 (0.61-1.18) | 0.95 (0.64-1.37) | 0.08 (-0.29-0.71) |
| Turkey | 309 (200-439) | 772 (528-1032) | 1.5 (0.8-2.65) | 0.95 (0.61-1.33) | 0.92 (0.63-1.23) | -0.02 (-0.29-0.41) |
| Turkmenistan | 8 (6-10) | 67 (43-94) | 7.67 (5.56-10.27) | 0.44 (0.34-0.55) | 1.71 (1.12-2.35) | 2.84 (1.94-3.96) |
| Tuvalu | 0 (0-0) | 0 (0-0) | 0.16 (-0.16-0.64) | 2.44 (1.55-3.62) | 1.87 (1.16-2.73) | -0.23 (-0.44-0.06) |
| Uganda | 53 (34-76) | 165 (107-234) | 2.11 (1.28-3.21) | 0.9 (0.59-1.25) | 1.31 (0.87-1.85) | 0.46 (0.1-0.93) |
| Ukraine | 205 (178-234) | 534 (436-650) | 1.6 (1.2-2.08) | 0.29 (0.25-0.33) | 0.68 (0.56-0.84) | 1.37 (1-1.8) |
| United Arab Emirates | 5 (2-9) | 46 (17-113) | 8.84 (4.49-14.84) | 1.53 (0.72-3.09) | 1.58 (0.63-3.86) | 0.03 (-0.39-0.56) |
| United Kingdom | 669 (597-747) | 2341 (1911-2840) | 2.5 (1.95-3.1) | 0.73 (0.65-0.81) | 1.79 (1.46-2.17) | 1.45 (1.06-1.88) |
| United Republic of Tanzania | 46 (30-63) | 114 (77-159) | 1.51 (0.96-2.23) | 0.48 (0.33-0.65) | 0.54 (0.36-0.74) | 0.11 (-0.12-0.4) |
| United States of America | 2485 (2235-2716) | 10409 (8344-12654) | 3.19 (2.47-3.95) | 0.78 (0.71-0.86) | 1.88 (1.51-2.28) | 1.4 (0.98-1.83) |
| United States Virgin Islands | 1 (0-1) | 1 (1-1) | 0.34 (0-0.74) | 0.94 (0.64-1.32) | 0.52 (0.35-0.72) | -0.44 (-0.58--0.28) |
| Uruguay | 19 (14-25) | 44 (30-60) | 1.31 (0.73-2) | 0.48 (0.34-0.62) | 0.78 (0.52-1.09) | 0.64 (0.24-1.14) |
| Uzbekistan | 36 (27-44) | 411 (275-564) | 10.56 (8.18-13.31) | 0.34 (0.26-0.42) | 2.26 (1.61-3) | 5.7 (4.54-6.99) |
| Vanuatu | 1 (1-2) | 3 (2-5) | 1.61 (0.79-2.91) | 2 (1.15-3.32) | 1.92 (1.16-2.88) | -0.04 (-0.32-0.41) |
| Venezuela (Bolivarian Republic of) | 215 (160-270) | 227 (152-319) | 0.06 (-0.2-0.36) | 2.36 (1.77-2.97) | 0.81 (0.55-1.13) | -0.66 (-0.74--0.56) |
| Viet Nam | 347 (192-542) | 536 (308-842) | 0.54 (0.06-1.39) | 0.91 (0.52-1.41) | 0.65 (0.38-1.01) | -0.29 (-0.51-0.11) |
| Yemen | 64 (37-103) | 175 (115-252) | 1.71 (0.91-2.92) | 1.5 (0.87-2.35) | 1.51 (1.02-2.15) | 0 (-0.28-0.42) |
| Zambia | 13 (9-20) | 36 (23-51) | 1.67 (0.78-2.83) | 0.56 (0.36-0.84) | 0.65 (0.43-0.91) | 0.17 (-0.22-0.62) |
| Zimbabwe | 139 (95-202) | 325 (215-462) | 1.33 (0.61-2.27) | 3.69 (2.59-5.26) | 5.02 (3.37-7.07) | 0.36 (-0.05-0.91) |
|  |  |  |  |  |  |  |

**ASIR: age-standardized incidence rate**

| **Table S2. The death of liver cancer due to hepatitis C between 1990 and 2019 at national level, both sexes** | | | | | | |
| --- | --- | --- | --- | --- | --- | --- |
| **Region** | **Case in 1990** | **Case in 2019** | **Change in**  **absolute number**  **(95% UI)** | **ASMR in 1990** | **ASMR in 2019** | **change in**  **ASMR per 100 000**  **population**  **(95% UI)** |
| Afghanistan | 318 (214-442) | 451 (300-635) | 0.42 (0.02-0.99) | 4.81 (3.32-6.52) | 4.13 (2.91-5.72) | -0.14 (-0.37-0.14) |
| Albania | 67 (47-88) | 81 (51-120) | 0.21 (-0.12-0.64) | 3.62 (2.61-4.72) | 1.85 (1.19-2.73) | -0.49 (-0.62--0.31) |
| Algeria | 73 (50-100) | 256 (178-350) | 2.5 (1.5-3.71) | 0.75 (0.54-0.97) | 0.89 (0.64-1.21) | 0.2 (-0.12-0.55) |
| American Samoa | 0 (0-0) | 1 (0-1) | 1.6 (1.01-2.4) | 1.37 (0.9-1.88) | 1.53 (1.05-2.08) | 0.12 (-0.13-0.44) |
| Andorra | 2 (1-3) | 5 (3-7) | 2.06 (1.01-3.51) | 3.33 (2.19-4.98) | 3.51 (2.33-4.97) | 0.05 (-0.3-0.53) |
| Angola | 45 (31-61) | 114 (81-155) | 1.53 (0.82-2.55) | 1.39 (1-1.84) | 1.26 (0.93-1.65) | -0.1 (-0.33-0.24) |
| Antigua and Barbuda | 1 (1-1) | 1 (0-1) | -0.4 (-0.5--0.28) | 1.94 (1.34-2.62) | 0.71 (0.49-0.98) | -0.64 (-0.7--0.57) |
| Argentina | 164 (118-221) | 425 (313-539) | 1.59 (1.22-2.1) | 0.53 (0.39-0.7) | 0.77 (0.57-0.98) | 0.46 (0.26-0.74) |
| Armenia | 11 (8-13) | 114 (81-150) | 9.72 (7.83-11.87) | 0.45 (0.34-0.55) | 2.78 (2-3.62) | 5.23 (4.1-6.44) |
| Australia | 115 (84-149) | 589 (431-763) | 4.14 (3.6-4.74) | 0.59 (0.43-0.76) | 1.37 (1-1.78) | 1.34 (1.12-1.59) |
| Austria | 121 (89-158) | 256 (187-342) | 1.12 (0.86-1.38) | 0.99 (0.73-1.28) | 1.37 (0.99-1.85) | 0.39 (0.22-0.56) |
| Azerbaijan | 18 (13-23) | 129 (85-188) | 6.18 (4.18-9.07) | 0.4 (0.3-0.5) | 1.72 (1.13-2.58) | 3.32 (2.05-5.29) |
| Bahamas | 3 (2-3) | 3 (2-4) | 0.04 (-0.17-0.3) | 1.76 (1.23-2.39) | 0.72 (0.48-1) | -0.59 (-0.67--0.49) |
| Bahrain | 3 (2-4) | 13 (9-18) | 3.29 (2.16-4.76) | 2.52 (1.82-3.25) | 2.25 (1.6-2.96) | -0.11 (-0.33-0.17) |
| Bangladesh | 562 (403-760) | 1502 (1055-1991) | 1.67 (0.96-2.62) | 1.3 (0.94-1.75) | 1.24 (0.89-1.61) | -0.04 (-0.29-0.27) |
| Barbados | 2 (1-3) | 3 (2-5) | 0.48 (0.11-1.03) | 0.69 (0.45-1.02) | 0.64 (0.44-0.91) | -0.07 (-0.3-0.27) |
| Belarus | 47 (34-61) | 99 (64-142) | 1.11 (0.62-1.72) | 0.37 (0.27-0.47) | 0.61 (0.4-0.87) | 0.65 (0.26-1.14) |
| Belgium | 188 (144-233) | 349 (265-438) | 0.85 (0.66-1.06) | 1.18 (0.9-1.45) | 1.42 (1.07-1.81) | 0.21 (0.09-0.34) |
| Belize | 1 (1-2) | 2 (1-3) | 0.22 (0-0.47) | 1.68 (1.19-2.24) | 0.74 (0.5-1.02) | -0.56 (-0.64--0.47) |
| Benin | 26 (16-37) | 44 (28-63) | 0.73 (0.29-1.37) | 1.41 (0.91-1.98) | 1.08 (0.71-1.52) | -0.23 (-0.42-0.04) |
| Bermuda | 1 (1-1) | 1 (0-1) | -0.39 (-0.51--0.22) | 1.71 (1.18-2.3) | 0.45 (0.29-0.64) | -0.74 (-0.79--0.68) |
| Bhutan | 2 (1-2) | 6 (4-9) | 2.72 (1.54-4.47) | 0.71 (0.44-1.1) | 1.12 (0.7-1.73) | 0.57 (0.12-1.25) |
| Bolivia (Plurinational State of) | 13 (7-22) | 36 (20-59) | 1.74 (0.96-2.82) | 0.48 (0.27-0.81) | 0.47 (0.27-0.75) | -0.03 (-0.3-0.34) |
| Bosnia and Herzegovina | 62 (43-84) | 150 (103-214) | 1.41 (0.84-2.1) | 1.67 (1.17-2.2) | 2.5 (1.74-3.5) | 0.5 (0.18-0.89) |
| Botswana | 2 (1-3) | 5 (3-8) | 2.18 (0.65-5.18) | 0.35 (0.17-0.66) | 0.46 (0.3-0.64) | 0.32 (-0.3-1.56) |
| Brazil | 684 (609-763) | 2374 (2082-2653) | 2.47 (2.27-2.71) | 0.86 (0.77-0.96) | 1.04 (0.91-1.15) | 0.2 (0.13-0.29) |
| Brunei Darussalam | 3 (2-4) | 10 (7-14) | 2.76 (1.96-3.81) | 3.86 (2.66-5.23) | 4.39 (3.24-5.71) | 0.14 (-0.08-0.44) |
| Bulgaria | 191 (129-260) | 155 (103-227) | -0.19 (-0.36-0.02) | 1.55 (1.09-2.05) | 1.05 (0.69-1.51) | -0.33 (-0.46--0.16) |
| Burkina Faso | 36 (24-49) | 51 (34-71) | 0.44 (0.04-0.98) | 0.99 (0.69-1.33) | 0.68 (0.45-0.94) | -0.31 (-0.5--0.07) |
| Burundi | 19 (11-29) | 30 (18-46) | 0.57 (0.04-1.38) | 0.91 (0.57-1.34) | 0.81 (0.5-1.22) | -0.11 (-0.39-0.29) |
| Cabo Verde | 1 (0-1) | 10 (7-14) | 14.89 (11.24-20.66) | 0.27 (0.18-0.37) | 2.56 (1.68-3.63) | 8.5 (6.26-11.94) |
| Cambodia | 188 (131-250) | 415 (296-550) | 1.2 (0.57-2.22) | 4.67 (3.32-6.02) | 3.92 (2.84-5.08) | -0.16 (-0.39-0.2) |
| Cameroon | 5 (3-8) | 12 (7-18) | 1.46 (0.65-2.51) | 0.13 (0.08-0.2) | 0.12 (0.08-0.18) | -0.07 (-0.36-0.32) |
| Canada | 114 (80-155) | 521 (356-734) | 3.58 (3.04-4.2) | 0.35 (0.25-0.48) | 0.73 (0.5-1.02) | 1.07 (0.84-1.32) |
| Central African Republic | 18 (11-26) | 29 (17-44) | 0.66 (0.2-1.29) | 1.72 (1.13-2.46) | 1.62 (1.01-2.37) | -0.06 (-0.29-0.26) |
| Chad | 37 (23-53) | 57 (36-83) | 0.54 (0.18-1.07) | 1.42 (0.91-2.01) | 1.21 (0.77-1.72) | -0.15 (-0.34-0.12) |
| Chile | 73 (53-93) | 266 (192-344) | 2.63 (2.19-3.14) | 0.77 (0.57-0.98) | 1.1 (0.8-1.42) | 0.42 (0.26-0.61) |
| China | 37163 (30837-45223) | 33079 (27212-39258) | -0.11 (-0.29-0.12) | 4.99 (4.19-5.99) | 1.75 (1.45-2.07) | -0.65 (-0.72--0.56) |
| Colombia | 190 (144-234) | 541 (372-750) | 1.85 (1.21-2.67) | 1.2 (0.92-1.47) | 1.02 (0.69-1.41) | -0.16 (-0.35-0.09) |
| Comoros | 2 (1-3) | 4 (2-6) | 1.12 (0.5-2.38) | 0.89 (0.52-1.42) | 0.86 (0.54-1.31) | -0.04 (-0.31-0.43) |
| Congo | 20 (13-28) | 33 (22-49) | 0.71 (0.25-1.36) | 2.09 (1.47-2.88) | 1.55 (1.08-2.19) | -0.26 (-0.45--0.01) |
| Cook Islands | 0 (0-1) | 1 (0-1) | 0.6 (0.26-1.05) | 3.24 (2.16-4.38) | 2.39 (1.61-3.34) | -0.26 (-0.41--0.07) |
| Costa Rica | 37 (27-46) | 100 (68-137) | 1.74 (1.13-2.53) | 2.21 (1.66-2.8) | 1.99 (1.35-2.72) | -0.1 (-0.3-0.17) |
| Croatia | 42 (29-58) | 62 (40-89) | 0.46 (0.14-0.88) | 0.69 (0.47-0.93) | 0.67 (0.43-0.96) | -0.03 (-0.24-0.23) |
| Cuba | 175 (125-233) | 111 (74-158) | -0.36 (-0.49--0.22) | 1.7 (1.2-2.23) | 0.56 (0.37-0.8) | -0.67 (-0.73--0.59) |
| Cyprus | 10 (7-13) | 26 (19-33) | 1.61 (1.09-2.43) | 1.38 (1-1.78) | 1.4 (1.05-1.79) | 0.02 (-0.17-0.29) |
| Czechia | 145 (100-195) | 151 (101-216) | 0.04 (-0.18-0.3) | 1.04 (0.73-1.38) | 0.69 (0.45-0.98) | -0.34 (-0.47--0.19) |
| Democratic People's Republic of Korea | 495 (312-713) | 756 (492-1071) | 0.53 (0.11-1.07) | 3.49 (2.26-4.87) | 2.44 (1.6-3.41) | -0.3 (-0.49--0.07) |
| Democratic Republic of the Congo | 178 (129-233) | 366 (257-498) | 1.06 (0.47-1.93) | 1.33 (1.01-1.68) | 1.17 (0.85-1.55) | -0.11 (-0.34-0.22) |
| Denmark | 57 (42-73) | 137 (100-177) | 1.4 (1.13-1.7) | 0.68 (0.5-0.86) | 1.15 (0.85-1.49) | 0.7 (0.51-0.91) |
| Djibouti | 1 (1-2) | 4 (2-7) | 3.41 (2.13-5.2) | 0.89 (0.55-1.44) | 0.92 (0.57-1.47) | 0.04 (-0.24-0.38) |
| Dominica | 2 (1-2) | 1 (0-1) | -0.58 (-0.67--0.46) | 2.28 (1.63-3.09) | 0.79 (0.52-1.11) | -0.66 (-0.73--0.56) |
| Dominican Republic | 33 (23-44) | 98 (62-152) | 1.96 (0.98-3.3) | 1 (0.72-1.33) | 1.12 (0.72-1.72) | 0.12 (-0.24-0.6) |
| Ecuador | 13 (8-19) | 47 (29-71) | 2.7 (1.74-3.84) | 0.27 (0.17-0.4) | 0.35 (0.21-0.52) | 0.28 (-0.04-0.66) |
| Egypt | 3360 (2517-4136) | 8629 (5849-12345) | 1.57 (0.68-3.05) | 11.9 (8.94-14.63) | 14.05 (9.83-19.71) | 0.18 (-0.21-0.83) |
| El Salvador | 43 (33-53) | 51 (35-71) | 0.19 (-0.12-0.57) | 1.5 (1.15-1.87) | 0.84 (0.57-1.18) | -0.44 (-0.59--0.26) |
| Equatorial Guinea | 3 (2-4) | 6 (4-10) | 1.23 (0.19-2.57) | 1.62 (1.14-2.2) | 1.61 (0.93-2.47) | 0 (-0.46-0.57) |
| Eritrea | 7 (4-13) | 20 (12-30) | 1.78 (0.87-3.27) | 0.92 (0.53-1.53) | 0.98 (0.62-1.44) | 0.06 (-0.27-0.61) |
| Estonia | 12 (9-16) | 26 (17-36) | 1.14 (0.64-1.76) | 0.59 (0.42-0.77) | 0.91 (0.59-1.29) | 0.54 (0.17-0.94) |
| Eswatini | 5 (3-8) | 23 (8-43) | 3.84 (0.26-9.48) | 1.88 (1.16-3.2) | 4.46 (1.64-8.17) | 1.37 (-0.33-4.01) |
| Ethiopia | 200 (136-284) | 422 (328-537) | 1.11 (0.31-2.18) | 1.21 (0.86-1.65) | 1.21 (0.95-1.55) | 0 (-0.34-0.47) |
| Fiji | 4 (3-6) | 10 (6-14) | 1.2 (0.59-2.17) | 1.48 (0.98-2.11) | 1.5 (1.02-2.14) | 0.01 (-0.25-0.43) |
| Finland | 81 (60-102) | 203 (152-257) | 1.52 (1.21-1.87) | 1.1 (0.83-1.38) | 1.52 (1.13-1.94) | 0.39 (0.23-0.57) |
| France | 1605 (1202-2056) | 3504 (2649-4375) | 1.18 (0.87-1.55) | 1.92 (1.44-2.5) | 2.45 (1.84-3.11) | 0.28 (0.1-0.48) |
| Gabon | 9 (7-13) | 16 (10-24) | 0.7 (0.1-1.58) | 1.84 (1.31-2.42) | 1.77 (1.15-2.59) | -0.04 (-0.37-0.47) |
| Gambia | 14 (8-20) | 53 (33-78) | 2.84 (1.62-4.51) | 4.46 (2.85-6.4) | 6.04 (3.83-8.89) | 0.35 (-0.06-0.88) |
| Georgia | 26 (20-34) | 71 (51-95) | 1.7 (1.21-2.28) | 0.46 (0.35-0.58) | 1.16 (0.83-1.54) | 1.53 (1.04-2.1) |
| Germany | 978 (755-1220) | 2413 (1784-3079) | 1.47 (1.15-1.8) | 0.75 (0.58-0.93) | 1.17 (0.87-1.49) | 0.56 (0.36-0.76) |
| Ghana | 60 (38-88) | 138 (86-204) | 1.28 (0.57-2.25) | 1.16 (0.75-1.66) | 1.02 (0.66-1.46) | -0.12 (-0.4-0.23) |
| Greece | 69 (48-94) | 166 (115-227) | 1.39 (1.06-1.79) | 0.46 (0.33-0.61) | 0.59 (0.41-0.8) | 0.27 (0.12-0.45) |
| Greenland | 0 (0-1) | 1 (1-2) | 1.86 (1.12-2.79) | 1.26 (0.89-1.71) | 1.74 (1.15-2.52) | 0.38 (0.05-0.79) |
| Grenada | 2 (1-2) | 1 (1-1) | -0.59 (-0.66--0.5) | 2.32 (1.62-3.12) | 0.73 (0.5-1) | -0.69 (-0.74--0.63) |
| Guam | 1 (0-1) | 2 (1-3) | 2.36 (1.59-3.31) | 0.92 (0.63-1.27) | 0.96 (0.63-1.4) | 0.04 (-0.2-0.32) |
| Guatemala | 141 (106-178) | 219 (157-294) | 0.55 (0.21-1.01) | 4.5 (3.46-5.61) | 2.13 (1.55-2.85) | -0.53 (-0.63--0.4) |
| Guinea | 179 (118-254) | 296 (182-436) | 0.65 (0.16-1.25) | 6 (3.94-8.41) | 6.04 (3.67-8.9) | 0.01 (-0.28-0.36) |
| Guinea-Bissau | 6 (3-9) | 8 (5-12) | 0.4 (0.02-0.92) | 1.62 (0.99-2.5) | 1.36 (0.84-2.09) | -0.16 (-0.37-0.14) |
| Guyana | 5 (4-8) | 4 (3-6) | -0.24 (-0.42--0.02) | 1.62 (1.12-2.23) | 0.75 (0.49-1.06) | -0.53 (-0.64--0.4) |
| Haiti | 52 (29-84) | 65 (35-106) | 0.25 (-0.11-0.74) | 1.9 (1.09-3.14) | 1.1 (0.6-1.73) | -0.42 (-0.59--0.2) |
| Honduras | 94 (31-148) | 376 (154-605) | 3.01 (2.07-4.89) | 4.99 (1.63-7.85) | 6.82 (2.82-10.85) | 0.37 (0.05-1.03) |
| Hungary | 241 (168-322) | 131 (88-184) | -0.46 (-0.56--0.34) | 1.65 (1.16-2.16) | 0.64 (0.43-0.9) | -0.61 (-0.68--0.52) |
| Iceland | 2 (2-3) | 6 (4-8) | 1.78 (1.38-2.23) | 0.74 (0.56-0.93) | 1.03 (0.76-1.35) | 0.4 (0.2-0.63) |
| India | 2031 (1592-2531) | 6292 (4887-7983) | 2.1 (1.45-2.74) | 0.57 (0.45-0.7) | 0.61 (0.47-0.77) | 0.07 (-0.15-0.29) |
| Indonesia | 1072 (902-1289) | 2163 (1784-2521) | 1.02 (0.59-1.54) | 1.29 (1.08-1.54) | 1.22 (1.02-1.39) | -0.05 (-0.25-0.18) |
| Iran (Islamic Republic of) | 272 (209-355) | 720 (607-840) | 1.65 (0.95-2.59) | 1.42 (1.08-1.86) | 1.12 (0.94-1.31) | -0.21 (-0.43-0.08) |
| Iraq | 127 (88-175) | 480 (326-666) | 2.76 (1.66-4.32) | 1.8 (1.27-2.42) | 2.43 (1.7-3.31) | 0.35 (-0.03-0.87) |
| Ireland | 24 (18-30) | 101 (76-129) | 3.24 (2.69-3.91) | 0.58 (0.44-0.72) | 1.31 (0.99-1.68) | 1.27 (0.99-1.61) |
| Israel | 61 (47-76) | 149 (116-185) | 1.45 (1.13-1.82) | 1.26 (0.97-1.55) | 1.25 (0.97-1.56) | 0 (-0.12-0.14) |
| Italy | 3121 (2853-3391) | 4032 (3535-4452) | 0.29 (0.17-0.38) | 3.43 (3.13-3.73) | 2.66 (2.34-2.93) | -0.22 (-0.28--0.17) |
| Jamaica | 18 (13-24) | 20 (13-28) | 0.09 (-0.15-0.38) | 0.99 (0.7-1.3) | 0.65 (0.43-0.94) | -0.34 (-0.49--0.17) |
| Japan | 13060 (12273-13760) | 25052 (21086-27540) | 0.92 (0.68-1.07) | 7.63 (7.16-8.03) | 6.08 (5.3-6.63) | -0.2 (-0.28--0.15) |
| Jordan | 13 (8-18) | 46 (31-62) | 2.58 (1.51-4.11) | 1.22 (0.8-1.68) | 0.88 (0.61-1.19) | -0.28 (-0.49-0.04) |
| Kazakhstan | 147 (108-188) | 372 (259-502) | 1.52 (1.14-1.96) | 1.23 (0.9-1.55) | 2.3 (1.62-3.05) | 0.88 (0.6-1.19) |
| Kenya | 47 (32-80) | 155 (102-227) | 2.29 (1.61-3.12) | 0.66 (0.46-1.13) | 0.86 (0.58-1.23) | 0.29 (0.04-0.58) |
| Kiribati | 1 (1-2) | 2 (1-3) | 0.59 (0.12-1.26) | 3.36 (2.28-4.69) | 3 (1.93-4.33) | -0.11 (-0.36-0.24) |
| Kuwait | 5 (3-6) | 18 (12-24) | 2.68 (1.82-3.7) | 0.98 (0.7-1.25) | 0.89 (0.63-1.2) | -0.09 (-0.3-0.14) |
| Kyrgyzstan | 15 (11-19) | 42 (31-55) | 1.82 (1.34-2.37) | 0.52 (0.39-0.65) | 1.06 (0.77-1.37) | 1.04 (0.68-1.42) |
| Lao People's Democratic Republic | 55 (32-86) | 74 (47-104) | 0.33 (-0.09-0.96) | 2.98 (1.78-4.46) | 1.98 (1.31-2.75) | -0.34 (-0.53--0.05) |
| Latvia | 17 (12-23) | 30 (20-41) | 0.73 (0.38-1.12) | 0.47 (0.34-0.62) | 0.69 (0.46-0.95) | 0.45 (0.16-0.78) |
| Lebanon | 17 (11-25) | 36 (23-58) | 1.14 (0.4-2.4) | 0.85 (0.56-1.21) | 0.7 (0.44-1.11) | -0.18 (-0.45-0.28) |
| Lesotho | 15 (9-28) | 44 (20-74) | 1.88 (-0.02-4.96) | 1.76 (1.05-3.13) | 3.97 (1.91-6.49) | 1.26 (-0.19-3.5) |
| Liberia | 14 (9-20) | 18 (11-28) | 0.31 (-0.05-0.83) | 1.39 (0.91-1.94) | 1.09 (0.67-1.64) | -0.22 (-0.42-0.07) |
| Libya | 33 (22-48) | 84 (55-123) | 1.56 (0.63-2.81) | 1.96 (1.29-2.83) | 1.83 (1.21-2.66) | -0.06 (-0.4-0.39) |
| Lithuania | 21 (15-28) | 45 (30-62) | 1.1 (0.66-1.58) | 0.47 (0.34-0.6) | 0.73 (0.48-1) | 0.56 (0.24-0.91) |
| Luxembourg | 6 (4-8) | 14 (10-20) | 1.42 (0.98-1.93) | 1.07 (0.8-1.38) | 1.37 (0.98-1.88) | 0.28 (0.05-0.55) |
| Madagascar | 33 (20-52) | 59 (36-91) | 0.82 (0.29-1.57) | 0.73 (0.45-1.17) | 0.69 (0.45-1.05) | -0.05 (-0.32-0.32) |
| Malawi | 27 (17-41) | 45 (30-63) | 0.7 (0.24-1.37) | 0.81 (0.52-1.18) | 0.77 (0.52-1.07) | -0.05 (-0.29-0.29) |
| Malaysia | 86 (55-123) | 264 (169-395) | 2.06 (1.28-3.19) | 1.07 (0.69-1.49) | 1.12 (0.73-1.65) | 0.05 (-0.22-0.42) |
| Maldives | 1 (1-2) | 4 (3-5) | 1.97 (0.83-3.75) | 2 (1.15-3.25) | 1.58 (1.04-2.2) | -0.21 (-0.47-0.22) |
| Mali | 218 (147-302) | 426 (277-606) | 0.95 (0.44-1.58) | 5.5 (3.82-7.37) | 5.28 (3.56-7.22) | -0.04 (-0.28-0.24) |
| Malta | 3 (2-4) | 8 (6-11) | 1.9 (1.45-2.45) | 0.68 (0.51-0.86) | 0.85 (0.63-1.1) | 0.26 (0.07-0.49) |
| Marshall Islands | 0 (0-1) | 1 (0-1) | 0.71 (0.21-1.36) | 2.88 (1.84-4.3) | 2.47 (1.52-3.65) | -0.14 (-0.38-0.16) |
| Mauritania | 14 (8-20) | 17 (11-25) | 0.26 (-0.07-0.68) | 1.51 (0.96-2.13) | 0.94 (0.61-1.36) | -0.37 (-0.52--0.16) |
| Mauritius | 4 (3-5) | 10 (6-14) | 1.67 (1.04-2.45) | 0.55 (0.39-0.7) | 0.59 (0.39-0.83) | 0.07 (-0.18-0.37) |
| Mexico | 417 (376-458) | 1736 (1460-2060) | 3.16 (2.61-3.8) | 1.1 (0.99-1.2) | 1.56 (1.31-1.86) | 0.42 (0.24-0.64) |
| Micronesia (Federated States of) | 1 (1-2) | 1 (1-2) | 0.33 (-0.14-0.99) | 2.77 (1.72-4.09) | 2.53 (1.54-3.79) | -0.09 (-0.38-0.32) |
| Monaco | 1 (1-1) | 3 (2-4) | 2.22 (1.39-3.36) | 1.23 (0.84-1.71) | 2.93 (2.06-3.93) | 1.38 (0.76-2.26) |
| Mongolia | 215 (146-299) | 697 (473-971) | 2.24 (1.4-3.46) | 21.95 (15.12-29.98) | 40.31 (28.58-53.28) | 0.84 (0.38-1.47) |
| Montenegro | 9 (6-13) | 15 (10-22) | 0.68 (0.26-1.33) | 1.51 (1.02-2.07) | 1.55 (1.07-2.18) | 0.03 (-0.23-0.43) |
| Morocco | 88 (57-122) | 231 (154-314) | 1.61 (0.91-2.62) | 0.79 (0.51-1.07) | 0.88 (0.6-1.19) | 0.12 (-0.18-0.53) |
| Mozambique | 22 (13-35) | 61 (36-90) | 1.77 (0.52-3.35) | 0.48 (0.29-0.76) | 0.72 (0.45-1.07) | 0.51 (-0.16-1.35) |
| Myanmar | 197 (122-305) | 594 (409-794) | 2.01 (1.11-3.27) | 1.02 (0.66-1.51) | 1.46 (1.02-1.93) | 0.43 (0.02-0.99) |
| Namibia | 4 (3-7) | 13 (9-19) | 2.04 (0.88-3.88) | 0.67 (0.42-1.09) | 1.05 (0.71-1.44) | 0.56 (-0.03-1.45) |
| Nauru | 0 (0-0) | 0 (0-0) | -0.09 (-0.35-0.28) | 2.53 (1.67-3.58) | 2.18 (1.38-3.28) | -0.14 (-0.36-0.14) |
| Nepal | 53 (34-76) | 164 (109-253) | 2.12 (1.12-3.35) | 0.69 (0.46-0.97) | 0.87 (0.58-1.3) | 0.25 (-0.13-0.71) |
| Netherlands | 94 (70-120) | 330 (244-418) | 2.52 (2.16-2.93) | 0.46 (0.35-0.59) | 0.92 (0.69-1.17) | 0.99 (0.8-1.21) |
| New Zealand | 23 (20-26) | 88 (76-101) | 2.86 (2.44-3.34) | 0.59 (0.51-0.68) | 1.11 (0.95-1.27) | 0.89 (0.7-1.11) |
| Nicaragua | 19 (14-24) | 68 (49-90) | 2.6 (1.81-3.52) | 1.34 (0.99-1.71) | 1.72 (1.27-2.25) | 0.29 (0.01-0.61) |
| Niger | 3 (2-5) | 9 (6-13) | 1.72 (1.02-2.63) | 0.15 (0.1-0.22) | 0.14 (0.09-0.2) | -0.03 (-0.27-0.27) |
| Nigeria | 334 (241-449) | 608 (459-781) | 0.82 (0.3-1.58) | 0.88 (0.64-1.18) | 0.86 (0.66-1.07) | -0.03 (-0.29-0.39) |
| Niue | 0 (0-0) | 0 (0-0) | -0.22 (-0.43-0.06) | 1.99 (1.33-2.81) | 1.66 (1.09-2.4) | -0.17 (-0.39-0.12) |
| North Macedonia | 44 (31-60) | 78 (51-112) | 0.76 (0.35-1.28) | 2.52 (1.77-3.34) | 2.55 (1.73-3.55) | 0.01 (-0.22-0.29) |
| Northern Mariana Islands | 0 (0-0) | 1 (0-1) | 2.24 (1.42-3.32) | 1.64 (1.08-2.25) | 1.54 (1-2.13) | -0.06 (-0.26-0.18) |
| Norway | 44 (39-49) | 98 (84-113) | 1.23 (1.02-1.49) | 0.62 (0.56-0.7) | 0.99 (0.85-1.13) | 0.58 (0.43-0.77) |
| Oman | 10 (6-14) | 23 (16-32) | 1.44 (0.71-2.58) | 1.73 (1.1-2.47) | 1.76 (1.23-2.3) | 0.02 (-0.29-0.53) |
| Pakistan | 1094 (705-1511) | 1941 (1486-2483) | 0.77 (0.27-1.66) | 2.01 (1.28-2.79) | 1.94 (1.48-2.5) | -0.03 (-0.29-0.42) |
| Palau | 0 (0-0) | 0 (0-0) | 1.12 (0.43-2.18) | 1.74 (1.06-2.6) | 1.69 (1.11-2.38) | -0.03 (-0.33-0.43) |
| Palestine | 31 (21-43) | 55 (39-71) | 0.79 (0.23-1.51) | 3.99 (2.75-5.47) | 2.89 (2.13-3.75) | -0.28 (-0.5-0.02) |
| Panama | 19 (14-24) | 47 (32-65) | 1.43 (0.83-2.18) | 1.34 (1.01-1.69) | 1.13 (0.77-1.59) | -0.16 (-0.37-0.09) |
| Papua New Guinea | 6 (4-9) | 16 (10-24) | 1.67 (0.94-2.66) | 0.47 (0.3-0.66) | 0.51 (0.34-0.73) | 0.09 (-0.19-0.46) |
| Paraguay | 20 (15-26) | 44 (30-61) | 1.22 (0.63-2) | 0.96 (0.71-1.24) | 0.85 (0.58-1.17) | -0.12 (-0.35-0.19) |
| Peru | 50 (30-74) | 69 (42-107) | 0.4 (-0.03-0.98) | 0.46 (0.28-0.68) | 0.22 (0.13-0.34) | -0.53 (-0.67--0.34) |
| Philippines | 580 (424-735) | 1079 (858-1341) | 0.86 (0.4-1.66) | 2.33 (1.74-2.92) | 1.56 (1.26-1.92) | -0.33 (-0.49--0.07) |
| Poland | 965 (839-1089) | 376 (297-460) | -0.61 (-0.67--0.54) | 2.31 (2.03-2.59) | 0.51 (0.41-0.62) | -0.78 (-0.81--0.74) |
| Portugal | 99 (75-126) | 404 (302-512) | 3.08 (2.64-3.55) | 0.72 (0.56-0.91) | 1.63 (1.21-2.09) | 1.27 (1.04-1.52) |
| Puerto Rico | 60 (41-80) | 48 (31-69) | -0.2 (-0.39-0.01) | 1.66 (1.15-2.2) | 0.62 (0.4-0.91) | -0.63 (-0.72--0.52) |
| Qatar | 4 (3-6) | 26 (16-38) | 4.93 (2.96-7.77) | 6.94 (4.47-9.83) | 6.48 (4.41-9.04) | -0.07 (-0.35-0.3) |
| Republic of Korea | 487 (326-693) | 2707 (1879-3574) | 4.55 (3.16-6.41) | 1.98 (1.35-2.75) | 3.08 (2.18-4.04) | 0.56 (0.19-1.02) |
| Republic of Moldova | 15 (11-20) | 33 (23-46) | 1.21 (0.82-1.63) | 0.36 (0.26-0.49) | 0.57 (0.39-0.79) | 0.56 (0.3-0.82) |
| Romania | 128 (90-172) | 284 (193-394) | 1.22 (0.78-1.73) | 0.48 (0.35-0.64) | 0.74 (0.5-1.03) | 0.52 (0.23-0.86) |
| Russian Federation | 823 (708-937) | 1913 (1585-2318) | 1.32 (1.01-1.65) | 0.47 (0.41-0.53) | 0.8 (0.66-0.97) | 0.7 (0.48-0.94) |
| Rwanda | 32 (19-51) | 59 (40-84) | 0.87 (0.24-1.81) | 1.28 (0.79-1.93) | 1.19 (0.82-1.67) | -0.07 (-0.35-0.34) |
| Saint Kitts and Nevis | 1 (1-2) | 1 (0-1) | -0.59 (-0.67--0.49) | 3.43 (2.46-4.5) | 0.93 (0.62-1.28) | -0.73 (-0.77--0.67) |
| Saint Lucia | 1 (1-2) | 1 (1-1) | -0.18 (-0.32--0.01) | 1.55 (1.11-2.05) | 0.51 (0.35-0.72) | -0.67 (-0.73--0.6) |
| Saint Vincent and the Grenadines | 1 (1-2) | 1 (1-1) | -0.34 (-0.45--0.23) | 1.9 (1.36-2.5) | 0.67 (0.46-0.93) | -0.65 (-0.7--0.59) |
| Samoa | 1 (1-2) | 1 (1-2) | 0.39 (0.04-0.84) | 1.29 (0.84-1.86) | 1.07 (0.69-1.55) | -0.17 (-0.37-0.07) |
| San Marino | 0 (0-0) | 1 (0-1) | 1.68 (0.72-2.95) | 0.77 (0.54-1.02) | 1 (0.61-1.53) | 0.31 (-0.16-0.96) |
| Sao Tome and Principe | 0 (0-0) | 0 (0-1) | 0.53 (0.06-1.18) | 0.53 (0.36-0.73) | 0.53 (0.32-0.82) | 0 (-0.31-0.4) |
| Saudi Arabia | 113 (72-160) | 246 (163-345) | 1.18 (0.39-2.35) | 2.31 (1.48-3.26) | 1.97 (1.36-2.71) | -0.15 (-0.44-0.29) |
| Senegal | 7 (4-10) | 15 (9-23) | 1.21 (0.56-2.06) | 0.24 (0.15-0.35) | 0.23 (0.14-0.35) | -0.05 (-0.32-0.3) |
| Serbia | 224 (155-309) | 305 (209-427) | 0.36 (0-0.81) | 2.09 (1.48-2.84) | 1.87 (1.3-2.56) | -0.11 (-0.33-0.17) |
| Seychelles | 1 (1-2) | 1 (1-2) | 0.22 (0-0.49) | 2.1 (1.48-2.83) | 1.44 (1-1.93) | -0.32 (-0.44--0.17) |
| Sierra Leone | 22 (14-31) | 32 (20-47) | 0.49 (0.06-1.07) | 1.23 (0.79-1.8) | 1.05 (0.66-1.51) | -0.15 (-0.39-0.19) |
| Singapore | 41 (27-55) | 193 (129-260) | 3.76 (3.1-4.54) | 2.08 (1.43-2.8) | 2.65 (1.77-3.55) | 0.27 (0.1-0.47) |
| Slovakia | 64 (45-86) | 77 (50-111) | 0.2 (-0.08-0.53) | 1.07 (0.76-1.44) | 0.82 (0.54-1.18) | -0.23 (-0.41--0.02) |
| Slovenia | 19 (13-29) | 61 (40-87) | 2.16 (1.18-3.36) | 0.79 (0.52-1.16) | 1.33 (0.86-1.9) | 0.68 (0.16-1.34) |
| Solomon Islands | 2 (1-3) | 3 (2-5) | 0.86 (0.3-1.71) | 1.44 (0.87-2.08) | 1.27 (0.86-1.75) | -0.12 (-0.35-0.25) |
| Somalia | 19 (10-34) | 51 (29-89) | 1.61 (0.81-2.8) | 0.96 (0.55-1.58) | 0.99 (0.59-1.76) | 0.03 (-0.26-0.45) |
| South Africa | 343 (222-533) | 684 (580-824) | 0.99 (0.23-2.01) | 1.8 (1.15-2.79) | 1.7 (1.45-2.04) | -0.06 (-0.42-0.44) |
| South Sudan | 17 (10-29) | 24 (13-42) | 0.41 (-0.01-0.97) | 0.83 (0.49-1.41) | 0.78 (0.44-1.3) | -0.07 (-0.33-0.27) |
| Spain | 1102 (880-1341) | 2735 (2182-3275) | 1.48 (1.21-1.79) | 1.99 (1.6-2.42) | 2.7 (2.1-3.3) | 0.36 (0.22-0.51) |
| Sri Lanka | 54 (37-73) | 181 (116-266) | 2.33 (1.35-3.7) | 0.61 (0.43-0.82) | 0.77 (0.5-1.12) | 0.27 (-0.09-0.76) |
| Sudan | 102 (56-159) | 194 (111-313) | 0.89 (0.32-1.75) | 1.26 (0.69-1.94) | 1.24 (0.72-1.96) | -0.02 (-0.3-0.43) |
| Suriname | 4 (3-5) | 4 (2-5) | -0.08 (-0.28-0.15) | 1.61 (1.14-2.17) | 0.63 (0.43-0.9) | -0.61 (-0.69--0.51) |
| Sweden | 157 (134-182) | 237 (198-277) | 0.5 (0.38-0.64) | 1 (0.86-1.16) | 1.08 (0.91-1.27) | 0.08 (-0.01-0.17) |
| Switzerland | 84 (62-109) | 289 (211-377) | 2.42 (2.04-2.89) | 0.78 (0.57-1.02) | 1.58 (1.15-2.08) | 1.02 (0.79-1.28) |
| Syrian Arab Republic | 89 (59-126) | 179 (121-252) | 1 (0.34-2.08) | 2.02 (1.31-2.82) | 1.75 (1.22-2.4) | -0.13 (-0.41-0.34) |
| Taiwan (Province of China) | 502 (363-642) | 1043 (721-1425) | 1.08 (0.6-1.69) | 3.26 (2.4-4.08) | 2.6 (1.81-3.56) | -0.2 (-0.38-0.02) |
| Tajikistan | 14 (11-17) | 79 (58-105) | 4.77 (3.44-6.44) | 0.52 (0.41-0.63) | 2.1 (1.57-2.75) | 3.05 (2.15-4.17) |
| Thailand | 1296 (874-1802) | 4319 (2729-6416) | 2.33 (1.41-3.62) | 4.2 (2.91-5.7) | 4.3 (2.73-6.36) | 0.03 (-0.25-0.39) |
| Timor-Leste | 5 (3-8) | 14 (9-21) | 1.67 (0.83-2.88) | 2.3 (1.44-3.46) | 1.94 (1.27-2.81) | -0.15 (-0.39-0.19) |
| Togo | 15 (9-21) | 33 (21-47) | 1.26 (0.63-2.13) | 1.4 (0.94-1.95) | 1.13 (0.74-1.6) | -0.19 (-0.4-0.11) |
| Tokelau | 0 (0-0) | 0 (0-0) | -0.26 (-0.48-0.04) | 2.24 (1.44-3.15) | 1.78 (1.18-2.53) | -0.21 (-0.43-0.11) |
| Tonga | 3 (2-4) | 4 (3-6) | 0.54 (0.13-1.03) | 5.07 (3.22-7.5) | 5.15 (3.28-7.5) | 0.02 (-0.24-0.34) |
| Trinidad and Tobago | 14 (10-18) | 11 (7-17) | -0.18 (-0.38-0.07) | 1.79 (1.27-2.34) | 0.63 (0.4-0.92) | -0.65 (-0.73--0.54) |
| Tunisia | 43 (30-59) | 106 (71-155) | 1.45 (0.61-2.84) | 0.97 (0.67-1.28) | 0.89 (0.6-1.3) | -0.07 (-0.39-0.44) |
| Turkey | 332 (216-466) | 707 (477-947) | 1.13 (0.56-2.09) | 1.05 (0.69-1.44) | 0.86 (0.58-1.14) | -0.18 (-0.4-0.19) |
| Turkmenistan | 8 (6-10) | 68 (45-95) | 7.22 (5.24-9.66) | 0.49 (0.38-0.6) | 1.79 (1.19-2.44) | 2.63 (1.79-3.69) |
| Tuvalu | 0 (0-0) | 0 (0-0) | 0.18 (-0.14-0.65) | 2.68 (1.72-3.98) | 2.07 (1.3-3) | -0.23 (-0.44-0.07) |
| Uganda | 57 (37-81) | 176 (116-250) | 2.09 (1.28-3.14) | 0.99 (0.66-1.38) | 1.46 (0.98-2.04) | 0.47 (0.12-0.92) |
| Ukraine | 225 (196-256) | 573 (467-690) | 1.55 (1.16-1.98) | 0.32 (0.28-0.36) | 0.73 (0.6-0.89) | 1.27 (0.93-1.67) |
| United Arab Emirates | 5 (2-9) | 43 (16-107) | 8.23 (4.14-13.82) | 1.7 (0.79-3.45) | 1.7 (0.67-4.26) | 0 (-0.41-0.52) |
| United Kingdom | 664 (590-739) | 2087 (1831-2335) | 2.14 (1.95-2.32) | 0.72 (0.64-0.79) | 1.55 (1.36-1.73) | 1.16 (1.05-1.27) |
| United Republic of Tanzania | 49 (33-68) | 126 (84-172) | 1.56 (1-2.31) | 0.55 (0.38-0.74) | 0.62 (0.42-0.84) | 0.11 (-0.12-0.4) |
| United States of America | 2436 (2192-2666) | 9231 (7939-10567) | 2.79 (2.4-3.13) | 0.76 (0.68-0.83) | 1.63 (1.4-1.87) | 1.16 (0.94-1.35) |
| United States Virgin Islands | 1 (1-1) | 1 (1-1) | 0.35 (0.02-0.76) | 1.05 (0.72-1.46) | 0.58 (0.39-0.79) | -0.45 (-0.58--0.29) |
| Uruguay | 21 (15-27) | 49 (35-62) | 1.35 (0.98-1.75) | 0.52 (0.38-0.67) | 0.85 (0.61-1.1) | 0.62 (0.37-0.88) |
| Uzbekistan | 39 (30-48) | 417 (280-573) | 9.74 (7.55-12.41) | 0.38 (0.29-0.47) | 2.48 (1.8-3.26) | 5.6 (4.5-6.86) |
| Vanuatu | 1 (1-2) | 3 (2-5) | 1.61 (0.81-2.88) | 2.22 (1.28-3.66) | 2.12 (1.27-3.15) | -0.05 (-0.32-0.38) |
| Venezuela (Bolivarian Republic of) | 231 (173-288) | 249 (169-346) | 0.08 (-0.17-0.38) | 2.58 (1.95-3.23) | 0.9 (0.62-1.25) | -0.65 (-0.73--0.55) |
| Viet Nam | 383 (213-595) | 579 (333-897) | 0.51 (0.05-1.38) | 1.03 (0.6-1.6) | 0.72 (0.43-1.11) | -0.3 (-0.51-0.1) |
| Yemen | 68 (40-108) | 184 (123-264) | 1.71 (0.91-2.85) | 1.66 (0.98-2.6) | 1.66 (1.13-2.33) | 0 (-0.29-0.41) |
| Zambia | 15 (9-22) | 39 (25-55) | 1.68 (0.81-2.8) | 0.64 (0.41-0.97) | 0.75 (0.51-1.04) | 0.17 (-0.22-0.63) |
| Zimbabwe | 145 (99-209) | 333 (220-473) | 1.3 (0.6-2.23) | 3.98 (2.82-5.67) | 5.36 (3.65-7.57) | 0.35 (-0.06-0.91) |
| ASMR: Age-standardized mortality rate | | | | | | |

| **Table S3. The disability-adjusted life years (DALYs) of liver cancer due to hepatitis C between 1990 and 2019 at national level, both sexes** | | | | | | |
| --- | --- | --- | --- | --- | --- | --- |
| **Region** | **Case in 1990** | **Case in 2019** | **Change in**  **absolute number**  **(95% UI)** | **ASDR in 1990** | **ASDR in 2019** | **change in**  **ASDR per 100 000**  **population**  **(95% UI)** |
| Afghanistan | 8136 (5302-11578) | 12474 (8130-17989) | 0.53 (0.05-1.24) | 110.39 (74.53-154.58) | 92.07 (61.13-130.59) | -0.17 (-0.4-0.17) |
| Albania | 1440 (995-1931) | 1567 (977-2348) | 0.09 (-0.23-0.48) | 70.84 (49.72-94.13) | 36.02 (22.59-54.01) | -0.49 (-0.63--0.31) |
| Algeria | 1674 (1125-2354) | 5421 (3680-7536) | 2.24 (1.31-3.41) | 14.15 (9.84-19.16) | 16.56 (11.5-22.91) | 0.17 (-0.15-0.55) |
| American Samoa | 6 (4-9) | 15 (10-22) | 1.45 (0.87-2.3) | 27.95 (18.06-39.74) | 31.67 (20.68-44.94) | 0.13 (-0.13-0.5) |
| Andorra | 36 (22-55) | 95 (61-136) | 1.66 (0.71-2.93) | 64.44 (40.61-97.73) | 67.87 (43.96-97.65) | 0.05 (-0.31-0.55) |
| Angola | 1211 (823-1664) | 2991 (2033-4130) | 1.47 (0.7-2.54) | 30.2 (21.16-40.94) | 26.12 (18.64-35.18) | -0.14 (-0.38-0.21) |
| Antigua and Barbuda | 20 (14-28) | 13 (9-19) | -0.36 (-0.48--0.22) | 38.61 (25.8-53.09) | 13.03 (8.77-18.75) | -0.66 (-0.72--0.59) |
| Argentina | 3327 (2362-4513) | 7923 (5755-10189) | 1.38 (1.04-1.86) | 10.27 (7.35-13.8) | 14.69 (10.64-18.95) | 0.43 (0.22-0.7) |
| Armenia | 234 (169-299) | 2215 (1549-3004) | 8.45 (6.69-10.53) | 8.86 (6.57-11.13) | 53.05 (37.48-70.79) | 4.99 (3.92-6.25) |
| Australia | 2438 (1757-3225) | 11236 (8049-14778) | 3.61 (3.16-4.15) | 12.54 (9.04-16.63) | 28.2 (20.05-37.4) | 1.25 (1.03-1.52) |
| Austria | 2377 (1716-3120) | 4659 (3306-6390) | 0.96 (0.73-1.21) | 20.63 (14.86-27.24) | 27.43 (19.17-37.62) | 0.33 (0.17-0.51) |
| Azerbaijan | 392 (284-509) | 3003 (1981-4347) | 6.65 (4.66-9.42) | 8.06 (5.94-10.3) | 32.96 (21.89-47.85) | 3.09 (2-4.68) |
| Bahamas | 57 (39-79) | 58 (37-83) | 0.02 (-0.2-0.3) | 37.09 (25.24-51.53) | 14.77 (9.55-20.8) | -0.6 (-0.69--0.49) |
| Bahrain | 70 (49-99) | 308 (199-445) | 3.4 (2.26-4.94) | 45.15 (31.45-60.63) | 37.16 (25.53-50.26) | -0.18 (-0.39-0.1) |
| Bangladesh | 14420 (10002-19949) | 33128 (23055-44348) | 1.3 (0.65-2.19) | 29.21 (20.45-39.91) | 25.21 (17.73-33.63) | -0.14 (-0.37-0.19) |
| Barbados | 39 (25-58) | 60 (39-85) | 0.54 (0.13-1.14) | 13.3 (8.28-20.21) | 12.01 (8.02-17.08) | -0.1 (-0.33-0.26) |
| Belarus | 961 (672-1274) | 1974 (1236-2979) | 1.05 (0.52-1.69) | 7.31 (5.18-9.6) | 12.35 (7.8-18.42) | 0.69 (0.25-1.22) |
| Belgium | 3373 (2514-4263) | 6027 (4514-7722) | 0.79 (0.6-1) | 21.88 (16.49-27.82) | 27.67 (20.67-35.93) | 0.26 (0.13-0.42) |
| Belize | 30 (21-41) | 40 (26-57) | 0.32 (0.08-0.59) | 33.01 (23.14-44.59) | 14.65 (9.73-20.85) | -0.56 (-0.64--0.47) |
| Benin | 573 (363-824) | 1012 (622-1500) | 0.77 (0.26-1.54) | 29.03 (18.27-41.63) | 21.18 (13.03-30.24) | -0.27 (-0.47-0.03) |
| Bermuda | 20 (13-27) | 10 (7-15) | -0.47 (-0.58--0.33) | 31.87 (21.57-43.93) | 7.92 (4.96-11.68) | -0.75 (-0.8--0.68) |
| Bhutan | 41 (23-65) | 123 (76-190) | 2.03 (1.03-3.62) | 15.94 (9.22-24.73) | 22.15 (13.64-34.04) | 0.39 (-0.03-1.05) |
| Bolivia (Plurinational State of) | 280 (149-484) | 692 (373-1161) | 1.47 (0.75-2.53) | 9.14 (4.97-15.46) | 8.2 (4.55-13.7) | -0.1 (-0.36-0.27) |
| Bosnia and Herzegovina | 1480 (998-2030) | 2873 (1882-4129) | 0.94 (0.47-1.54) | 35.46 (24.41-47.63) | 47.44 (31.33-67.96) | 0.34 (0.03-0.71) |
| Botswana | 41 (20-83) | 137 (83-204) | 2.34 (0.68-5.48) | 7.18 (3.47-14.25) | 9.64 (5.97-14.02) | 0.34 (-0.31-1.59) |
| Brazil | 15888 (14002-17854) | 49167 (42836-55644) | 2.09 (1.91-2.3) | 17.75 (15.76-19.82) | 20.7 (18.06-23.36) | 0.17 (0.1-0.24) |
| Brunei Darussalam | 67 (43-97) | 271 (181-382) | 3.04 (2.17-4.21) | 73.53 (49.01-103.93) | 86.49 (60.49-117.48) | 0.18 (-0.06-0.5) |
| Bulgaria | 4301 (2893-5972) | 3063 (1976-4523) | -0.29 (-0.45--0.09) | 33.45 (22.7-45.54) | 22.13 (14.38-32.6) | -0.34 (-0.48--0.15) |
| Burkina Faso | 834 (548-1169) | 1217 (790-1704) | 0.46 (0.04-1.05) | 19.45 (13.06-26.75) | 13.4 (8.89-18.44) | -0.31 (-0.5--0.06) |
| Burundi | 450 (260-706) | 732 (428-1157) | 0.63 (0.03-1.52) | 19.26 (11.25-29.85) | 16.2 (9.65-25.12) | -0.16 (-0.46-0.27) |
| Cabo Verde | 12 (8-17) | 191 (126-270) | 15.07 (11.28-20.62) | 5.13 (3.5-7.11) | 46.32 (30.13-65.76) | 8.03 (5.88-11.12) |
| Cambodia | 4972 (3353-6699) | 10002 (6703-13664) | 1.01 (0.4-1.92) | 105.43 (73.51-141.11) | 82.43 (56.92-109.92) | -0.22 (-0.44-0.14) |
| Cameroon | 116 (69-184) | 288 (172-444) | 1.48 (0.63-2.72) | 2.66 (1.57-4.18) | 2.45 (1.49-3.67) | -0.08 (-0.38-0.32) |
| Canada | 2371 (1602-3285) | 9885 (6656-13756) | 3.17 (2.71-3.73) | 7.42 (5.02-10.24) | 14.81 (10.09-20.52) | 1 (0.78-1.26) |
| Central African Republic | 475 (290-728) | 790 (457-1258) | 0.66 (0.19-1.3) | 38.91 (24.42-57.9) | 35.53 (21.46-53.92) | -0.09 (-0.34-0.25) |
| Chad | 835 (521-1208) | 1328 (818-1976) | 0.59 (0.19-1.16) | 29.68 (18.66-42.77) | 24.13 (14.75-35.63) | -0.19 (-0.38-0.08) |
| Chile | 1638 (1171-2114) | 5196 (3716-6836) | 2.17 (1.78-2.64) | 16.32 (11.66-20.91) | 21.43 (15.35-28.13) | 0.31 (0.16-0.5) |
| China | 909455 (744826-1114928) | 714427 (580678-856767) | -0.21 (-0.38--0.01) | 105.93 (87.73-128.71) | 35.11 (28.85-41.74) | -0.67 (-0.74--0.58) |
| Colombia | 4311 (3218-5395) | 10190 (6882-14502) | 1.36 (0.79-2.09) | 24.72 (18.67-30.87) | 19.33 (13.05-27.46) | -0.22 (-0.41-0.02) |
| Comoros | 40 (20-66) | 82 (50-126) | 1.04 (0.38-2.71) | 18.16 (9.65-29.72) | 16.93 (10.3-26.17) | -0.07 (-0.36-0.61) |
| Congo | 506 (330-748) | 854 (546-1289) | 0.69 (0.18-1.36) | 46.07 (31.1-66.24) | 32.01 (21.22-46.72) | -0.31 (-0.49--0.05) |
| Cook Islands | 8 (5-12) | 12 (8-18) | 0.44 (0.1-0.9) | 66.92 (43.31-93.94) | 48.56 (31.75-70.03) | -0.27 (-0.44--0.06) |
| Costa Rica | 769 (573-991) | 1986 (1317-2809) | 1.58 (0.98-2.37) | 44.46 (33.2-57.51) | 38.77 (25.67-54.88) | -0.13 (-0.33-0.14) |
| Croatia | 862 (566-1203) | 1112 (702-1649) | 0.29 (0-0.69) | 13.28 (8.85-18.35) | 12.77 (7.98-19.04) | -0.04 (-0.26-0.25) |
| Cuba | 3413 (2368-4494) | 1983 (1269-2902) | -0.42 (-0.54--0.29) | 33.06 (22.95-43.88) | 10.36 (6.61-15.19) | -0.69 (-0.75--0.61) |
| Cyprus | 204 (141-276) | 470 (335-609) | 1.3 (0.84-1.96) | 25.58 (18.17-33.98) | 24.6 (17.9-31.74) | -0.04 (-0.23-0.22) |
| Czechia | 2966 (2011-4033) | 2772 (1764-4113) | -0.07 (-0.26-0.18) | 21.33 (14.62-28.66) | 13.33 (8.64-19.49) | -0.38 (-0.5--0.21) |
| Democratic People's Republic of Korea | 12518 (7563-18529) | 17232 (10872-24805) | 0.38 (-0.02-0.88) | 76.12 (48-109.78) | 52.83 (33.78-75.72) | -0.31 (-0.5--0.07) |
| Democratic Republic of the Congo | 4740 (3388-6324) | 9730 (6709-13644) | 1.05 (0.42-1.99) | 28.75 (21.23-37.43) | 25.71 (17.91-35.19) | -0.11 (-0.36-0.27) |
| Denmark | 1108 (808-1435) | 2575 (1861-3423) | 1.32 (1.05-1.61) | 14.15 (10.27-18.34) | 23.58 (16.9-31.67) | 0.67 (0.47-0.87) |
| Djibouti | 25 (14-43) | 105 (57-183) | 3.15 (1.84-5.15) | 18.04 (10.57-30.17) | 17.91 (10.4-30.29) | -0.01 (-0.29-0.39) |
| Dominica | 32 (22-44) | 13 (9-20) | -0.58 (-0.67--0.45) | 44.64 (30.69-61.78) | 14.83 (9.59-21.8) | -0.67 (-0.74--0.58) |
| Dominican Republic | 732 (499-1005) | 1982 (1189-3227) | 1.71 (0.78-3.07) | 19.73 (13.68-27.11) | 21.5 (13.11-35.01) | 0.09 (-0.29-0.62) |
| Ecuador | 250 (156-368) | 826 (494-1273) | 2.31 (1.43-3.38) | 4.87 (3.05-7.17) | 5.68 (3.4-8.65) | 0.16 (-0.14-0.54) |
| Egypt | 90846 (67951-114018) | 231262 (151517-333273) | 1.55 (0.64-3) | 288.88 (218.04-357.98) | 333.1 (224.95-473.1) | 0.15 (-0.25-0.82) |
| El Salvador | 968 (718-1222) | 960 (634-1350) | -0.01 (-0.27-0.32) | 32.77 (24.51-41.45) | 16.36 (10.79-22.94) | -0.5 (-0.63--0.33) |
| Equatorial Guinea | 75 (48-109) | 151 (82-242) | 1.01 (0.08-2.33) | 36.55 (24.42-52.31) | 31.74 (17.71-49.79) | -0.13 (-0.54-0.4) |
| Eritrea | 196 (103-341) | 509 (305-786) | 1.6 (0.75-3.1) | 19.75 (10.66-34.11) | 19.54 (11.94-29.19) | -0.01 (-0.33-0.52) |
| Estonia | 253 (174-340) | 456 (291-660) | 0.8 (0.36-1.34) | 12.21 (8.46-16.36) | 17.66 (11.25-25.67) | 0.45 (0.09-0.86) |
| Eswatini | 116 (68-206) | 585 (189-1127) | 4.02 (0.22-10.52) | 39.23 (23.32-68.87) | 97.57 (32.34-187.53) | 1.49 (-0.36-4.55) |
| Ethiopia | 5183 (3386-7577) | 9282 (7144-12015) | 0.79 (0.06-1.83) | 25.78 (17.49-36.53) | 23.29 (17.94-29.7) | -0.1 (-0.44-0.38) |
| Fiji | 112 (69-168) | 237 (147-354) | 1.11 (0.5-2.15) | 31.07 (19.77-45.07) | 31.14 (20.08-45.61) | 0 (-0.28-0.46) |
| Finland | 1581 (1177-2025) | 3372 (2458-4414) | 1.13 (0.88-1.44) | 22.24 (16.41-28.8) | 28.19 (20.69-36.83) | 0.27 (0.12-0.44) |
| France | 33141 (24172-43784) | 63013 (46686-80864) | 0.9 (0.62-1.23) | 41.86 (30.36-54.9) | 50.31 (36.98-65.29) | 0.2 (0.03-0.41) |
| Gabon | 232 (157-316) | 388 (237-581) | 0.67 (0.06-1.59) | 40.53 (27.94-54.43) | 36.57 (22.81-54.51) | -0.1 (-0.43-0.39) |
| Gambia | 345 (204-523) | 1274 (765-1936) | 2.69 (1.39-4.48) | 95.67 (58-142.41) | 130.23 (79.25-195.59) | 0.36 (-0.09-0.99) |
| Georgia | 556 (403-726) | 1475 (1020-2055) | 1.65 (1.13-2.28) | 9 (6.66-11.56) | 25.52 (17.58-35.59) | 1.83 (1.24-2.54) |
| Germany | 18364 (14040-23462) | 41231 (30215-53410) | 1.25 (0.95-1.53) | 14.64 (11.17-18.71) | 22.56 (16.38-30.02) | 0.54 (0.34-0.74) |
| Ghana | 1465 (898-2216) | 3232 (1929-4849) | 1.21 (0.48-2.14) | 23.62 (14.89-34.51) | 20.29 (12.26-30.24) | -0.14 (-0.41-0.21) |
| Greece | 1169 (795-1575) | 2425 (1671-3367) | 1.07 (0.83-1.38) | 7.58 (5.22-10.11) | 10.11 (6.93-14.17) | 0.33 (0.18-0.5) |
| Greenland | 11 (7-16) | 29 (18-43) | 1.54 (0.83-2.42) | 30.18 (20.1-42.38) | 38.62 (24.74-56.22) | 0.28 (-0.04-0.68) |
| Grenada | 34 (23-45) | 16 (10-22) | -0.54 (-0.62--0.44) | 47.16 (32.17-64.27) | 13.95 (9.41-19.36) | -0.7 (-0.75--0.65) |
| Guam | 13 (8-19) | 41 (25-62) | 2.2 (1.43-3.14) | 17.57 (11.46-24.86) | 21.31 (13.42-31.68) | 0.21 (-0.07-0.56) |
| Guatemala | 3361 (2478-4293) | 4683 (3287-6520) | 0.39 (0.07-0.85) | 92.15 (69.6-116.04) | 42.78 (30.09-59.09) | -0.54 (-0.64--0.4) |
| Guinea | 4067 (2667-5831) | 6723 (3943-9987) | 0.65 (0.15-1.27) | 123.21 (81.36-174.15) | 122.86 (72.99-181.41) | 0 (-0.31-0.37) |
| Guinea-Bissau | 143 (82-235) | 199 (112-317) | 0.39 (-0.01-0.95) | 34.95 (20.32-56.03) | 27.82 (16.39-43.68) | -0.2 (-0.42-0.11) |
| Guyana | 127 (84-183) | 95 (58-142) | -0.25 (-0.45--0.01) | 33.71 (22.61-48.41) | 15.17 (9.46-22.28) | -0.55 (-0.66--0.41) |
| Haiti | 1219 (649-2016) | 1507 (793-2465) | 0.24 (-0.12-0.74) | 38.4 (21.18-62.68) | 22.11 (11.88-35.89) | -0.42 (-0.59--0.2) |
| Honduras | 2115 (737-3383) | 8085 (3260-13327) | 2.82 (1.88-4.59) | 103.3 (35.02-165.42) | 135.41 (54.48-221.81) | 0.31 (0-0.93) |
| Hungary | 4897 (3405-6615) | 2354 (1499-3393) | -0.52 (-0.61--0.4) | 32.96 (23.08-44.24) | 12.37 (7.92-17.96) | -0.62 (-0.7--0.54) |
| Iceland | 43 (32-54) | 109 (78-146) | 1.56 (1.19-1.99) | 15.25 (11.44-19.56) | 20.24 (14.4-27.11) | 0.33 (0.13-0.55) |
| India | 50299 (39315-62768) | 140423 (109064-179042) | 1.79 (1.22-2.38) | 11.48 (9.07-14.24) | 12.38 (9.62-15.67) | 0.08 (-0.15-0.3) |
| Indonesia | 26458 (22133-31825) | 47858 (39063-56927) | 0.81 (0.41-1.29) | 26.89 (22.54-32.36) | 23.15 (19.11-27.1) | -0.14 (-0.32-0.09) |
| Iran (Islamic Republic of) | 6141 (4760-8011) | 14287 (12024-16932) | 1.33 (0.74-2.11) | 25.82 (19.9-33.62) | 20.4 (17.12-23.9) | -0.21 (-0.42-0.07) |
| Iraq | 2946 (1970-4153) | 11410 (7499-16381) | 2.87 (1.69-4.64) | 38.01 (25.69-53.03) | 49.73 (33.18-70.11) | 0.31 (-0.08-0.88) |
| Ireland | 459 (343-583) | 1822 (1335-2363) | 2.97 (2.43-3.57) | 11.24 (8.43-14.18) | 24.53 (17.95-31.62) | 1.18 (0.9-1.51) |
| Israel | 1190 (891-1504) | 2761 (2072-3448) | 1.32 (1.02-1.69) | 24.46 (18.26-30.98) | 24.46 (18.36-30.74) | 0 (-0.12-0.16) |
| Italy | 64501 (58354-70834) | 71136 (62707-78795) | 0.1 (0.02-0.18) | 72.91 (66.05-79.9) | 53.9 (47.26-60.26) | -0.26 (-0.31--0.21) |
| Jamaica | 354 (245-474) | 377 (240-559) | 0.06 (-0.2-0.38) | 20 (13.74-26.99) | 12.71 (8.12-18.9) | -0.36 (-0.52--0.17) |
| Japan | 311963 (292281-330173) | 383625 (337521-418060) | 0.23 (0.1-0.32) | 178.83 (167.59-189.22) | 112.76 (101.23-122.52) | -0.37 (-0.41--0.33) |
| Jordan | 295 (193-415) | 1004 (665-1384) | 2.4 (1.38-3.82) | 23.46 (15.4-32.56) | 16.3 (11-22.39) | -0.31 (-0.51--0.01) |
| Kazakhstan | 3548 (2553-4654) | 8778 (6009-11957) | 1.47 (1.08-1.92) | 27.66 (20.11-35.83) | 49.61 (34.5-67.45) | 0.79 (0.51-1.1) |
| Kenya | 1094 (738-1906) | 3698 (2410-5547) | 2.38 (1.63-3.31) | 13.44 (9.13-23.35) | 17.02 (11.3-25.19) | 0.27 (0.01-0.59) |
| Kiribati | 30 (19-44) | 47 (29-70) | 0.6 (0.12-1.29) | 77.16 (50.7-111.92) | 65.89 (41.98-95.04) | -0.15 (-0.39-0.19) |
| Kuwait | 122 (83-164) | 392 (262-554) | 2.22 (1.43-3.17) | 20.19 (14.13-26.46) | 16.55 (11.2-22.95) | -0.18 (-0.38-0.06) |
| Kyrgyzstan | 324 (234-419) | 953 (663-1257) | 1.95 (1.38-2.57) | 10.77 (7.9-13.81) | 21.22 (15.02-27.69) | 0.97 (0.61-1.36) |
| Lao People's Democratic Republic | 1389 (760-2192) | 1743 (1069-2526) | 0.25 (-0.18-0.89) | 65.63 (37.61-101.68) | 40.16 (25.24-57.22) | -0.39 (-0.59--0.09) |
| Latvia | 355 (247-479) | 533 (354-750) | 0.5 (0.2-0.85) | 9.81 (6.87-13.13) | 13.75 (8.93-19.52) | 0.4 (0.12-0.74) |
| Lebanon | 376 (226-562) | 701 (421-1105) | 0.86 (0.22-1.93) | 16.76 (10.59-24.77) | 13.48 (8.06-21.3) | -0.2 (-0.47-0.21) |
| Lesotho | 358 (200-682) | 1107 (464-1901) | 2.09 (-0.03-5.56) | 36.5 (20.99-68.27) | 85.9 (38.24-146.39) | 1.35 (-0.23-3.95) |
| Liberia | 315 (197-459) | 422 (249-664) | 0.34 (-0.07-0.93) | 28.45 (18.01-40.65) | 21.08 (12.47-32.22) | -0.26 (-0.47-0.04) |
| Libya | 754 (483-1110) | 1978 (1238-2995) | 1.62 (0.62-3.01) | 40.81 (26.43-60.1) | 38.59 (24.74-57.72) | -0.05 (-0.41-0.43) |
| Lithuania | 441 (310-581) | 830 (540-1144) | 0.88 (0.47-1.35) | 9.7 (6.85-12.75) | 15.02 (9.68-20.86) | 0.55 (0.21-0.92) |
| Luxembourg | 116 (84-152) | 256 (178-354) | 1.21 (0.82-1.73) | 21.17 (15.51-27.91) | 25.97 (17.93-36.26) | 0.23 (0.01-0.51) |
| Madagascar | 791 (484-1283) | 1523 (911-2377) | 0.92 (0.34-1.78) | 15.32 (9.47-24.85) | 13.96 (8.59-21.39) | -0.09 (-0.36-0.29) |
| Malawi | 663 (407-1021) | 997 (634-1400) | 0.5 (0.08-1.12) | 17.09 (10.83-25.97) | 14.49 (9.32-20.16) | -0.15 (-0.38-0.18) |
| Malaysia | 1924 (1205-2766) | 5632 (3564-8674) | 1.93 (1.16-3.03) | 21.58 (13.62-30.82) | 21.42 (13.62-31.89) | -0.01 (-0.27-0.37) |
| Maldives | 33 (17-60) | 80 (52-114) | 1.47 (0.48-2.96) | 38.32 (21.22-67.1) | 28.06 (18.39-39.44) | -0.27 (-0.53-0.15) |
| Mali | 5768 (3818-8183) | 11061 (6965-16327) | 0.92 (0.39-1.58) | 131.08 (88.05-181.99) | 122.21 (78.52-176.53) | -0.07 (-0.32-0.24) |
| Malta | 58 (43-75) | 150 (108-200) | 1.58 (1.16-2.09) | 13.53 (10.03-17.27) | 16.79 (12.15-22.52) | 0.24 (0.04-0.47) |
| Marshall Islands | 10 (6-15) | 18 (10-29) | 0.89 (0.32-1.66) | 60.97 (37.88-93.76) | 52.22 (31.08-78.59) | -0.14 (-0.38-0.18) |
| Mauritania | 311 (188-452) | 364 (219-538) | 0.17 (-0.17-0.61) | 31.17 (19.21-45.2) | 17.93 (11.09-26.04) | -0.42 (-0.58--0.21) |
| Mauritius | 80 (55-105) | 189 (121-282) | 1.37 (0.8-2.09) | 11.01 (7.65-14.34) | 10.86 (7.1-15.9) | -0.01 (-0.24-0.28) |
| Mexico | 9017 (8075-9948) | 35486 (29513-42435) | 2.94 (2.39-3.58) | 21.48 (19.3-23.66) | 30.54 (25.48-36.54) | 0.42 (0.23-0.65) |
| Micronesia (Federated States of) | 27 (16-42) | 38 (20-63) | 0.4 (-0.12-1.07) | 59.03 (35.81-90.37) | 52.72 (29.74-83.22) | -0.11 (-0.41-0.32) |
| Monaco | 17 (11-23) | 52 (35-70) | 2.11 (1.29-3.29) | 24.99 (16.38-35.13) | 57.84 (39.39-79.22) | 1.31 (0.67-2.23) |
| Mongolia | 5292 (3483-7497) | 16530 (10580-23937) | 2.12 (1.26-3.33) | 501.73 (334.19-709.19) | 752.55 (511.89-1044.07) | 0.5 (0.11-1.08) |
| Montenegro | 200 (132-284) | 313 (203-454) | 0.57 (0.18-1.16) | 31.78 (21.15-44.64) | 31.65 (20.82-45.25) | 0 (-0.25-0.37) |
| Morocco | 1837 (1168-2593) | 4687 (3051-6524) | 1.55 (0.83-2.59) | 14.39 (9.34-19.92) | 15.91 (10.5-21.72) | 0.11 (-0.2-0.54) |
| Mozambique | 500 (293-816) | 1364 (771-2053) | 1.73 (0.47-3.28) | 8.88 (5.3-14.08) | 13.32 (7.95-19.87) | 0.5 (-0.19-1.35) |
| Myanmar | 4489 (2648-7169) | 13040 (8653-18080) | 1.9 (0.98-3.2) | 20.14 (12.34-31.34) | 28.65 (19.55-38.88) | 0.42 (-0.01-1.01) |
| Namibia | 102 (61-171) | 305 (192-447) | 1.98 (0.79-3.95) | 14.14 (8.56-23.34) | 21.65 (13.9-31.12) | 0.53 (-0.07-1.51) |
| Nauru | 2 (1-3) | 2 (1-3) | -0.01 (-0.3-0.37) | 53.31 (33.74-79.37) | 45.67 (27.09-70.21) | -0.14 (-0.37-0.15) |
| Nepal | 1261 (793-1901) | 3428 (2246-5425) | 1.72 (0.81-2.77) | 13.84 (8.96-20.06) | 15.95 (10.64-24.86) | 0.15 (-0.23-0.59) |
| Netherlands | 1765 (1302-2273) | 5712 (4178-7424) | 2.24 (1.91-2.62) | 9 (6.69-11.67) | 17.34 (12.61-22.47) | 0.93 (0.73-1.15) |
| New Zealand | 499 (427-581) | 1764 (1507-2045) | 2.54 (2.16-2.96) | 13.04 (11.16-15.2) | 23.79 (20.32-27.58) | 0.82 (0.63-1.04) |
| Nicaragua | 446 (318-577) | 1446 (1004-1961) | 2.24 (1.47-3.13) | 28.89 (20.74-37.54) | 33.34 (23.66-45.01) | 0.15 (-0.11-0.45) |
| Niger | 84 (53-124) | 220 (132-328) | 1.62 (0.92-2.59) | 3.04 (1.95-4.44) | 2.87 (1.77-4.21) | -0.06 (-0.3-0.26) |
| Nigeria | 7392 (5222-10036) | 13574 (9918-18079) | 0.84 (0.31-1.59) | 17.26 (12.3-23.22) | 16.42 (12.25-21.37) | -0.05 (-0.32-0.35) |
| Niue | 1 (1-1) | 1 (0-1) | -0.17 (-0.39-0.16) | 41.52 (27.34-60.09) | 34.29 (21.61-50.4) | -0.17 (-0.4-0.13) |
| North Macedonia | 1006 (678-1373) | 1631 (1035-2390) | 0.62 (0.24-1.11) | 53.05 (36.28-72.1) | 49.98 (32.7-72.05) | -0.06 (-0.27-0.22) |
| Northern Mariana Islands | 6 (4-9) | 18 (10-27) | 1.93 (1.09-3.06) | 33.72 (21.75-47.94) | 31.58 (20.18-44.85) | -0.06 (-0.27-0.2) |
| Norway | 835 (741-935) | 1793 (1536-2083) | 1.15 (0.94-1.41) | 13.01 (11.49-14.58) | 19.63 (16.72-22.86) | 0.51 (0.36-0.7) |
| Oman | 248 (152-369) | 615 (400-877) | 1.49 (0.72-2.72) | 37.07 (23.17-54.66) | 35.17 (23.43-47.33) | -0.05 (-0.33-0.4) |
| Pakistan | 27260 (18021-37356) | 51440 (39685-66366) | 0.89 (0.35-1.86) | 46.2 (30.14-63.65) | 43.65 (33.54-55.86) | -0.06 (-0.31-0.42) |
| Palau | 4 (2-6) | 8 (5-13) | 1.28 (0.49-2.5) | 37.32 (21.76-57.49) | 36.74 (22.92-53.75) | -0.02 (-0.34-0.48) |
| Palestine | 645 (416-923) | 1213 (833-1614) | 0.88 (0.28-1.7) | 76.89 (50.78-109.77) | 54.13 (38.48-71.23) | -0.3 (-0.52-0.01) |
| Panama | 421 (310-540) | 921 (617-1324) | 1.19 (0.65-1.9) | 28.19 (20.89-36.31) | 22.34 (14.92-32.18) | -0.21 (-0.4-0.05) |
| Papua New Guinea | 138 (86-204) | 357 (221-550) | 1.59 (0.85-2.64) | 8.52 (5.4-12.24) | 9.05 (5.8-13.35) | 0.06 (-0.24-0.45) |
| Paraguay | 429 (308-556) | 891 (589-1274) | 1.08 (0.51-1.84) | 19.56 (13.95-25.38) | 16.36 (10.85-22.99) | -0.16 (-0.39-0.13) |
| Peru | 1026 (628-1575) | 1216 (719-1996) | 0.19 (-0.18-0.69) | 8.86 (5.36-13.62) | 3.83 (2.24-6.36) | -0.57 (-0.7--0.39) |
| Philippines | 14129 (10381-18361) | 25798 (20152-32607) | 0.83 (0.37-1.61) | 47.29 (34.55-60.51) | 32.64 (25.89-41.24) | -0.31 (-0.48--0.02) |
| Poland | 18509 (15952-21154) | 6657 (5241-8148) | -0.64 (-0.7--0.57) | 42.45 (36.67-48.16) | 9.45 (7.47-11.56) | -0.78 (-0.81--0.74) |
| Portugal | 2047 (1522-2668) | 7536 (5439-9828) | 2.68 (2.29-3.1) | 14.78 (11.12-19.07) | 34.82 (24.71-46.34) | 1.36 (1.1-1.64) |
| Puerto Rico | 1134 (759-1536) | 826 (519-1233) | -0.27 (-0.45--0.06) | 30.99 (20.79-41.68) | 11.83 (7.37-17.87) | -0.62 (-0.71--0.51) |
| Qatar | 108 (69-163) | 668 (394-1005) | 5.17 (3.03-8.36) | 126.48 (80.99-181.1) | 102.35 (66.43-145.97) | -0.19 (-0.44-0.16) |
| Republic of Korea | 11716 (7555-16935) | 50729 (34099-68972) | 3.33 (2.2-4.83) | 39.3 (26.15-55.55) | 56 (38.15-75.45) | 0.43 (0.09-0.85) |
| Republic of Moldova | 345 (234-478) | 649 (432-939) | 0.88 (0.55-1.23) | 7.69 (5.3-10.49) | 11.13 (7.44-15.86) | 0.45 (0.2-0.72) |
| Romania | 2743 (1883-3779) | 5575 (3620-7901) | 1.03 (0.61-1.51) | 9.74 (6.83-13.2) | 15.59 (9.99-22.1) | 0.6 (0.27-0.99) |
| Russian Federation | 18353 (15501-21424) | 38784 (31503-47723) | 1.11 (0.82-1.44) | 10.04 (8.57-11.58) | 16.51 (13.38-20.2) | 0.64 (0.42-0.9) |
| Rwanda | 785 (447-1281) | 1414 (900-2074) | 0.8 (0.13-1.88) | 27.1 (15.93-42.78) | 23.73 (15.81-33.81) | -0.12 (-0.42-0.33) |
| Saint Kitts and Nevis | 25 (17-34) | 12 (7-18) | -0.53 (-0.64--0.41) | 67.1 (46.55-90.06) | 17.87 (11.67-25.93) | -0.73 (-0.78--0.67) |
| Saint Lucia | 26 (18-36) | 20 (13-30) | -0.22 (-0.36--0.05) | 29.93 (20.76-40.79) | 9.47 (6.18-13.68) | -0.68 (-0.74--0.61) |
| Saint Vincent and the Grenadines | 26 (18-36) | 18 (12-25) | -0.32 (-0.43--0.2) | 36.86 (25.41-49.86) | 13.17 (8.83-18.49) | -0.64 (-0.7--0.58) |
| Samoa | 25 (15-38) | 34 (21-52) | 0.38 (0-0.86) | 28.1 (17.64-42.75) | 23.09 (14.38-34.36) | -0.18 (-0.39-0.1) |
| San Marino | 5 (3-7) | 12 (7-18) | 1.43 (0.53-2.78) | 14.8 (10.16-20.03) | 19.65 (11.4-30.97) | 0.33 (-0.18-1.08) |
| Sao Tome and Principe | 7 (4-9) | 11 (6-16) | 0.6 (0.09-1.31) | 10.37 (6.86-14.4) | 10.21 (6.12-15.91) | -0.02 (-0.33-0.4) |
| Saudi Arabia | 2621 (1654-3861) | 6173 (3945-8845) | 1.36 (0.46-2.65) | 46.46 (29.37-66.88) | 38.37 (25.54-53.55) | -0.17 (-0.46-0.26) |
| Senegal | 149 (87-223) | 320 (188-486) | 1.15 (0.51-2.08) | 4.72 (2.83-7.01) | 4.39 (2.56-6.81) | -0.07 (-0.35-0.29) |
| Serbia | 5101 (3419-7153) | 5931 (3937-8402) | 0.16 (-0.15-0.59) | 44.21 (30.78-60.56) | 37.09 (24.75-52.77) | -0.16 (-0.37-0.11) |
| Seychelles | 25 (17-34) | 31 (21-45) | 0.27 (0.04-0.57) | 44 (29.61-60.56) | 28.45 (19.08-39.69) | -0.35 (-0.47--0.21) |
| Sierra Leone | 471 (291-716) | 727 (426-1058) | 0.54 (0.07-1.26) | 24.86 (15.53-37.5) | 20.6 (12.53-29.58) | -0.17 (-0.41-0.19) |
| Singapore | 895 (595-1235) | 3511 (2299-4892) | 2.93 (2.39-3.5) | 41.33 (27.81-56.94) | 45.52 (30.07-63.06) | 0.1 (-0.04-0.26) |
| Slovakia | 1384 (941-1898) | 1527 (974-2284) | 0.1 (-0.17-0.44) | 23 (15.65-31.52) | 16.53 (10.6-24.48) | -0.28 (-0.45--0.06) |
| Slovenia | 398 (255-594) | 1104 (698-1618) | 1.77 (0.87-2.93) | 16.12 (10.37-24.01) | 26.14 (16.27-38.9) | 0.62 (0.1-1.34) |
| Solomon Islands | 49 (28-77) | 89 (55-129) | 0.82 (0.24-1.69) | 33.3 (19.53-50.23) | 27.6 (17.86-39.21) | -0.17 (-0.41-0.19) |
| Somalia | 516 (264-915) | 1304 (735-2361) | 1.53 (0.68-2.76) | 20.38 (11.01-34.72) | 20.25 (11.69-35.59) | -0.01 (-0.31-0.45) |
| South Africa | 7961 (5274-12429) | 15418 (12906-18502) | 0.94 (0.2-1.87) | 37.15 (24.23-58.17) | 34.19 (28.75-40.91) | -0.08 (-0.43-0.38) |
| South Sudan | 399 (226-700) | 576 (301-1007) | 0.44 (-0.03-1.1) | 16.98 (9.69-29.42) | 15.32 (8.26-26.69) | -0.1 (-0.38-0.26) |
| Spain | 22297 (17564-27645) | 48902 (37655-60373) | 1.19 (0.94-1.46) | 40.94 (32.43-50.46) | 54.97 (41.91-68.27) | 0.34 (0.19-0.5) |
| Sri Lanka | 1172 (787-1621) | 3686 (2262-5591) | 2.15 (1.2-3.46) | 11.35 (7.69-15.56) | 14.42 (8.98-21.36) | 0.27 (-0.11-0.79) |
| Sudan | 2261 (1245-3509) | 4171 (2334-6769) | 0.84 (0.25-1.68) | 24.95 (13.77-38.35) | 23.67 (13.39-38.58) | -0.05 (-0.36-0.36) |
| Suriname | 85 (57-119) | 75 (48-109) | -0.12 (-0.32-0.11) | 32.99 (22.39-45.06) | 12.44 (8.17-18.12) | -0.62 (-0.71--0.53) |
| Sweden | 2916 (2476-3408) | 4225 (3500-4954) | 0.45 (0.33-0.58) | 20.5 (17.3-24.08) | 21.96 (18.13-26.2) | 0.07 (-0.02-0.17) |
| Switzerland | 1628 (1165-2140) | 5193 (3698-6882) | 2.19 (1.82-2.62) | 16.18 (11.67-21.34) | 31.38 (22.07-42.23) | 0.94 (0.71-1.21) |
| Syrian Arab Republic | 1980 (1272-2845) | 3975 (2579-5807) | 1.01 (0.33-2.11) | 38.95 (25.35-55.7) | 32.95 (22-46.75) | -0.15 (-0.43-0.3) |
| Taiwan (Province of China) | 12546 (9046-16496) | 19755 (13235-27598) | 0.57 (0.18-1.05) | 74.93 (54.94-97.01) | 49.46 (33.22-69.09) | -0.34 (-0.5--0.15) |
| Tajikistan | 308 (237-384) | 2002 (1402-2712) | 5.5 (3.95-7.48) | 11.17 (8.67-13.89) | 41.44 (30.42-54.53) | 2.71 (1.88-3.76) |
| Thailand | 30527 (20095-43126) | 87757 (53600-134944) | 1.87 (1.04-3) | 85.83 (57.65-120.05) | 84.85 (52.91-128.56) | -0.01 (-0.29-0.36) |
| Timor-Leste | 139 (78-218) | 312 (188-471) | 1.24 (0.49-2.46) | 49.09 (29.56-75.44) | 38.69 (24.15-57.31) | -0.21 (-0.46-0.14) |
| Togo | 356 (227-515) | 794 (472-1159) | 1.23 (0.59-2.13) | 28.74 (18.49-40.96) | 22.24 (13.76-31.88) | -0.23 (-0.44-0.07) |
| Tokelau | 1 (0-1) | 0 (0-1) | -0.25 (-0.49-0.08) | 48.25 (29.89-70.62) | 36.39 (23.37-52.91) | -0.25 (-0.48-0.07) |
| Tonga | 63 (39-95) | 89 (54-135) | 0.42 (0.04-0.93) | 110.25 (68.36-166.73) | 112.23 (67.93-169.45) | 0.02 (-0.25-0.38) |
| Trinidad and Tobago | 292 (202-392) | 229 (141-349) | -0.22 (-0.42-0.04) | 35.13 (24.28-47.04) | 12.28 (7.7-18.35) | -0.65 (-0.74--0.54) |
| Tunisia | 968 (665-1331) | 2252 (1465-3363) | 1.33 (0.52-2.71) | 19.26 (13.35-26.07) | 17.84 (11.66-26.56) | -0.07 (-0.39-0.45) |
| Turkey | 7295 (4611-10570) | 13087 (8616-17915) | 0.79 (0.29-1.63) | 20.77 (13.34-29.58) | 15.15 (10.03-20.62) | -0.27 (-0.47-0.06) |
| Turkmenistan | 185 (135-236) | 1832 (1174-2568) | 8.88 (6.44-11.82) | 9.98 (7.47-12.49) | 43.36 (28.6-60.59) | 3.34 (2.31-4.57) |
| Tuvalu | 4 (2-6) | 4 (3-7) | 0.1 (-0.22-0.57) | 57.84 (35.82-88.32) | 42.89 (26.04-64.21) | -0.26 (-0.47-0.04) |
| Uganda | 1373 (867-1988) | 4307 (2709-6186) | 2.14 (1.25-3.37) | 21.11 (13.5-30.07) | 30.26 (19.47-43.3) | 0.43 (0.05-0.94) |
| Ukraine | 4433 (3798-5097) | 11146 (8958-13728) | 1.51 (1.11-1.97) | 6.12 (5.28-6.98) | 14.68 (11.86-18.11) | 1.4 (1.01-1.85) |
| United Arab Emirates | 135 (68-264) | 1409 (521-3537) | 9.43 (4.73-16.25) | 32.85 (15.32-66.29) | 32.73 (12.46-79.78) | 0 (-0.42-0.53) |
| United Kingdom | 12531 (11123-14099) | 35044 (30906-39630) | 1.8 (1.65-1.93) | 14.27 (12.56-16.08) | 28.67 (25.18-32.52) | 1.01 (0.91-1.1) |
| United Republic of Tanzania | 1145 (743-1631) | 2779 (1826-3927) | 1.43 (0.84-2.12) | 10.77 (7.2-14.86) | 11.67 (7.8-16.34) | 0.08 (-0.16-0.38) |
| United States of America | 51425 (46179-56454) | 199612 (169775-231284) | 2.88 (2.46-3.25) | 16.86 (15.12-18.49) | 37.02 (31.37-42.8) | 1.2 (0.96-1.4) |
| United States Virgin Islands | 17 (11-25) | 21 (14-30) | 0.21 (-0.09-0.62) | 20.63 (13.63-29.06) | 11.15 (7.26-15.73) | -0.46 (-0.59--0.29) |
| Uruguay | 411 (290-541) | 881 (635-1156) | 1.14 (0.81-1.53) | 10.47 (7.45-13.76) | 16.76 (12.02-22.24) | 0.6 (0.34-0.89) |
| Uzbekistan | 823 (608-1045) | 11163 (7358-15385) | 12.56 (9.76-15.61) | 7.48 (5.62-9.43) | 52.05 (36.46-69.77) | 5.96 (4.71-7.37) |
| Vanuatu | 30 (17-50) | 78 (43-122) | 1.6 (0.78-2.91) | 46.26 (26.19-77.93) | 44.81 (25.47-68.44) | -0.03 (-0.33-0.47) |
| Venezuela (Bolivarian Republic of) | 5228 (3852-6642) | 5033 (3251-7189) | -0.04 (-0.28-0.26) | 54.55 (40.26-69.01) | 17.4 (11.39-24.74) | -0.68 (-0.76--0.58) |
| Viet Nam | 8035 (4467-12743) | 11612 (6250-18674) | 0.45 (-0.01-1.23) | 20.28 (11.36-31.85) | 13.16 (7.39-20.87) | -0.35 (-0.56-0) |
| Yemen | 1622 (934-2570) | 4243 (2744-6224) | 1.62 (0.79-2.9) | 34 (20.07-53.39) | 33.05 (21.76-47.94) | -0.03 (-0.32-0.41) |
| Zambia | 348 (214-538) | 913 (578-1324) | 1.62 (0.74-2.86) | 12.5 (7.9-19.1) | 14.21 (9.17-20.08) | 0.14 (-0.24-0.61) |
| Zimbabwe | 3518 (2359-5189) | 8450 (5545-12020) | 1.4 (0.63-2.41) | 84.92 (58.31-122.91) | 117.34 (77.64-166.82) | 0.38 (-0.05-0.94) |
| ASDR: age-standardized DALYs rate | | | | | | |
